# Item Response Model Adaptation for Analyzing Data from Different Versions of Parkinson’s Disease Rating Scales

**DOI:** 10.1007/s11095-019-2668-6

**Published:** 2019-07-17

**Authors:** Gopichand Gottipati, Alienor C. Berges, Shuying Yang, Chao Chen, Mats O. Karlsson, Elodie L. Plan

**Affiliations:** 1 Department of Pharmaceutical Biosciences, Uppsala University, Box 591, 75124 Uppsala, Sweden; 2 GlaxoSmithKline, London, UK; 3 Present Address: Quantitative Clinical Pharmacology, Early Clinical Development, IMED Biotech Unit, AstraZeneca, Cambridge, UK

**Keywords:** Data integration, disease progression, item response theory, movement disorder society (sponsored revision) – Unified parkinson’s disease rating scale, parkinson’s disease

## Abstract

**Purpose:**

The aim of this work was to allow combination of information from recent and historical trials in Parkinson’s Disease (PD) by developing bridging methodology between two versions of the clinical endpoint.

**Methods:**

A previously developed Item Response Model (IRM), that described longitudinal changes in Movement Disorder Society (MDS) sponsored revision of Unified Parkinson’s Disease Rating Scale (UPDRS) [MDS–UPDRS] data from the De Novo PD cohort in Parkinson’s Progression Markers Initiative, was first adapted to describe baseline UPDRS data from two clinical trials, one in subjects with early PD and another in subjects with advanced PD. Assuming similar IRM structure, items of the UPDRS version were mapped to those in the MDS-UPDRS version. Subsequently, the longitudinal changes in the placebo arm of the advanced PD study were characterized.

**Results:**

The parameters reflecting differences in the shared items between endpoints were successfully estimated, and the model diagnostics indicated that mapping was better for early PD subjects (closer to De Novo cohort) than for advanced PD subjects. Disease progression for placebo in advanced PD patients was relatively shallow.

**Conclusion:**

An IRM able to handle two variants of clinical PD endpoints was developed; it can improve the utilization of data from diverse sources and diverse disease populations.

**Electronic supplementary material:**

The online version of this article (10.1007/s11095-019-2668-6) contains supplementary material, which is available to authorized users.

## Introduction

Parkinson’s disease (PD) is a chronic, progressive neurological disorder and is manifested with the symptoms of bradykinesia, tremor, rigidity and postural instability. The underlying etiology is believed to be the loss of dopaminergic neurons in the substantia nigra located in the midbrain. It is associated with a significant disease burden. In the US, it affects about 1.5 million people with about 60,000 newly diagnosed patients each year (1). Furthermore, it costs the US economy directly (e.g. medical treatments, hospitalizations and nursing home care) or indirectly (e.g. reduced employment and loss in productivity) about $14 billion a year (2). The disease burden is expected to increase substantially in the next few decades due to the growing size of the elderly population. Therefore, the need to develop innovative new approaches for the early detection, the prevention, the delaying of the onset of the disease, and the alleviation of the symptoms, is more important now than ever. Yet, there are several challenges in the development of newer therapies, such as, unclear understanding of the pathophysiology of the disease, and paucity of objective biomarkers that can quantify and track the underlying disease progression (1,3). In the absence of sensitive and predictive biomarkers, clinical trials for drugs aiming to slow down the disease progression require large sample size and long duration.

The original Unified Parkinson’s Disease Rating Scale (UPDRS) has been used to assess the severity of PD for over three decades. The original UPDRS consists of six parts: Mentation (I), Activities of Daily Living (II), Motor Examination (III), Complication of Therapy (IV), Modified Hoehn and Yahr Staging (V), and Schwab and England Activities of Daily Living Scale (VI). Each part assesses different aspects of the disease (4), with higher scores typically reflecting a more severe disease status. The regulatory criteria for the therapies that seek approval for the treatments offering either symptomatic and/or disease modifying effect is based on the change in the “total” UPDRS score (sum of parts I, II and III) or in individual part(s), e.g., Part III - motor symptoms, relative to the baseline visit. More recently, a newer version of the UPDRS endpoint, the Movement Disorder Society – sponsored revision of UPDRS (MDS-UPDRS), was developed (5,6), as an attempt to, among other reasons, address the inconsistencies and resolve ambiguities in the UPDRS scale. More importantly, it also aims to detect the smaller changes in the disease early and measure the milder deficits, thereby making early prognosis of the disease and aiding in the development of the therapies for early intervention more likely. MDS-UPDRS is structurally similar to the UPDRS with four parts. In MDS-UPDRS the conceptual divisions in UPDRS (first four parts) are generally honored, but the titles and described items differ: Non-Motor Experiences of Daily Living (I), Motor Experiences of Daily Living (II), Motor Examination (III), and Motor Complications (IV). While Part I and IV of MDS-UPDRS differs substantially from UPDRS, Parts II and III of both the rating scales are very similar. This newer version hasn’t been extensively used in the PD trials yet. For both versions, traditional analyses of PD trials relied on the recorded scores and the use of a “total” score as sum of individual parts (either only certain subparts or all of them) (7,8).

It may be of merit to revisit the use of the total score because of the underlying assumptions it entails: (i) equal importance of each sub-score relative to the total score, (ii) equal discrimination of each item in the questionnaire, (iii) occurrence of missing data at random (when in fact the response to a specific item by a specific subject may be missing because he/she found it was difficult and intentionally refused to respond), and (iv) imputation of missing data with e.g., 0 or mean value (potentially incorporating a bias). Such underlying assumptions may not always be valid when conducting traditional analyses using the total scores. Furthermore, the other disadvantage of using the “total” score for analyses include the lack of flexibility to leverage/integrate knowledge from (i) multiple data sources because of the variable range in the score depending on various ways of summing scores (e.g., sum of parts I, II and III *vs.* sum of items based on part III only), and (ii) multiple/different (e.g., newer) versions of the endpoint categories, e.g., MDS-UPDRS *vs.* UPDRS in which there are qualitative/quantitative differences in the nature of the items in each scale. In summary, it is crucial to explore more innovative and model-based methodologies, which can address the shortcomings listed above and that can potentially improve the efficiencies in the analyses of PD trials in the future.

Item Response Theory (IRT) originated from the field of psychometrics and has been reported to be extensively used in the development and validation of questionnaires in patient-reported outcomes research (9,10). It is a statistical framework, which relates an individual’s underlying latent (or hidden) variable to the pattern of responses to the items on the assessment scale, and such a relationship is described by the Item Characteristic Curves (ICC). Following the first and seminal pharmacometrics application of the IRT methodology in Alzheimer’s disease (11), it was later applied in other therapeutic areas such as multiple sclerosis (12) and schizophrenia (13). Previous work in the PD area involved developing a longitudinal IRT model with three latent variables to adequately describe disease progression using MDS–UPDRS data in 430 De Novo subjects followed during 48 months (14). This model-based approach offered improved utilization of the MDS–UPDRS reported ratings, not only at the total score level but also at the individual item level. Although a few formulae have been reported in the literature (15–17) for converting either total score or sum of certain parts of the scale between UPDRS and MDS-UPDRS versions, potential for improved efficiency in clinical trial analyses formed the motivation for exploring IRT methodology for bridging between the two versions of the clinical endpoint.

Our objectives for the current manuscript were (i) to adapt the IRT model previously developed based on MDS-UPDRS (the newer version of the PD clinical endpoint) so that it can handle and describe baseline UPDRS (classical version of the PD clinical endpoint) from historical clinical trials, and subsequently (ii) to explore the utility of such an integrated framework to characterize disease progression in placebo PD subjects.

## Material and Methods

### Data and Patients

This work was performed using UPDRS data (parts I-III, i.e. items 1–31) from two clinical trials (Studies 168 and 169). Baseline observations from all arms of the two studies were included, as well as the longitudinal data from the placebo arm of Study 169.

Study 168 (18) was a multicenter, randomized, double-blind, three-period, two-treatment, non-inferiority crossover study of immediate-release (IR) and extended-release (ER) formulations of ropinirole in early PD patients (*N* = 161). Eligible subjects entered a 7-day placebo run-in period, and those who completed it, entered a 12-week dose-titration phase. In this phase, they were randomly assigned to one of the four three-period sequences for the subsequent maintenance phase: IR-IR-ER, IR-ER-ER, ER-ER-IR or ER-IR-IR.

Study 169 (19) was a multicenter, multinational, double-blind, parallel-group and placebo-controlled study of ropinirole in advanced PD patients (*N* = 393) that were inadequately controlled by levadopa. Eligible subjects entered a 14-day placebo run-in period and those who completed it were randomized 1:1 to receive once-daily, adjunctive treatment with ropinirole or placebo for 24 weeks. Subjects were evaluated at baseline and weeks 1, 2, 3, 4, 6, 8, 10, 12, 16, 20 and 24.

Seven of the 31 assessments in parts I, II and III of UPDRS included multiple evaluations for different parts and/or sides (e.g., left, right) of the body, resulting in a total of 44 items for each patient in both studies. For instance, tremor at rest was assessed in the head region (face, lips and chin), upper and lower extremities (hands and feet respectively), of which the latter two were also assessed on the left and right sides of the body. The other six such items include: action/postural tremor (right and left hands), rigidity (neck, upper and lower extremities on both sides), and each of finger taps, hand movements, hand pronation and supination and leg agility assessed on both sides of the body.

The data, including longitudinal and item-level information, were formatted in a way that allowed for an IRT analysis using the software NONMEM (20).

### MDS-UPDRS Item Response Model (IRM)

The model developed previously (14) to characterize the longitudinal changes in MDS – UPDRS data in subjects belonging to De Novo PD cohort from the Parkinson’s Progression Markers Initiative (PPMI) database was an item response probabilistic model with multiple latent variables. The subjects’ responses to each item were described by an ordered categorical model (using cumulative probabilities), depending on the nature of the item, as described below. The structural (non-longitudinal) components of the IRM were (1) item-specific fixed-effect parameters: *a*_*j*_, the slope or discrimination parameter (for an item *j*), and *b*_*jk*_, the difficulty parameter, representing the disability at which there is 50% probability of obtaining a positive response (denoted by *k*) for that item, and (2) subject (denoted by *i*)-specific random-effect parameter: *D*_*i*_, ‘disability’, a latent variable.

The probability that the subjects’ response was *k* (values between 0 and a maximum of *K* [i.e., either 4 or 5]) was characterized as a function of disability:

1 $$ {\displaystyle \begin{array}{l}P\left({Y}_{ij}\ge k\right)=\frac{e^{a_j\left({D}_i-{b}_{jk}\right)}}{1+{e}^{a_j\left({D}_i-{b}_{jk}\right)}}\\ {}\Big\{\begin{array}{l}P\left({Y}_{ij}=0\right)=1-P\left({Y}_{ij}\ge 1\right)\\ {}P\left({Y}_{ij}=k\right)=P\left({Y}_{ij}\ge k\right)-P\left({Y}_{ij}\ge k+1\right)\\ {}\ \end{array}\end{array}} $$

*where Y*_*ij*_ is the subjects’ observed response to *j*^*th*^ item.

The final IRM had the following characteristics: (i) Three latent variables describing Patient-reported responses (PR), Non-Sided Responses (NSR) and Sided-Responses (SR) – a latent variable for items that evaluated the right and left sides; (ii) A mixture component estimating the proportion of the two sub-populations depending on their most disabled (dominant) side at baseline (i.e., one whose more disabled side at baseline was the right side, and the other whose more disabled side at baseline was the left side). This mixture proportion was estimated to be 0.58, suggesting that there 58% probability that a subject belongs to subpopulation whose more disabled side was the right side at baseline. (iii) Progression rates of PR and NSR were similar (50 months for a typical subject to progress linearly by 1 standard deviation relative to the disability at baseline), and it varied for SR depending on if the items evaluated the side that was the more/less disabled side at baseline (4) A shift parameter (associated with a random effect) used to reflect the lower disability of the initially better side, since it was noted that the initially better side deteriorated quicker, and that its difference in disability with the initially more disabled side became smaller with time. The shift parameter was estimated to be 2.11 (SD = 0.60).

### Model Adaptation Workflow

As a first step, the two versions of the clinical endpoint were compared. We noticed that there were both (1) quantitative differences, i.e., there are 9 items in the UPDRS scale (item 13: *Falling,* 14: *Freezing*, 20: *Tremor at rest* – evaluated for face/lips/chin, hands and feet on left and right sides, 21:*Action/Postural Tremor* – evaluated for hands on left and right sides) that are not in the MDS-UPDRS version, as well as, (2) qualitative differences in the construct and interpretation of the items and the response categories between the two scales. An instance where the items differed qualitatively in the clinical descriptors for the responses is MDS-UPDRS item 3.8, which assesses the subject’s *leg agility*, rated between 0 and 4: 0-Normal, 1-Slight, 2-Mild, 3-Moderate, 4-Severe; while UPDRS item 26 *leg agility* is rated between response categories of 0-Normal, 1-Mild, 2-Moderate, 3-Severe. 4-Can barely perform task. This indicates that there is a contrast in the perceived severity between the two assessment scales.

While it may be of interest to probe into the rationale for the revision of the scale, we refer to Goetz *et al.* (6) work, where they proposed a comprehensive, well-defined mapping mechanism as a standard set of criteria to facilitate consistency between the different scales. This mapping strategy formed the basis for the current work on model adaptation between the two versions of the clinical endpoint in PD.

Broadly, the mappings of the items were categorized into: *(I)* Direct-mapping, *(II)* Indirect-mapping and *(III)* items that are exclusive to a particular version of the clinical endpoint. The procedure followed for each of mapping category was as follows. For items that are directly mapped, a similar model structure (excluding the longitudinal aspects) from the previously developed IRM based on the MDS-UPDRS data was assumed. The item-specific parameters were fixed to their final estimates in the previously developed IRM, i.e. no additional parameters were estimated unless there were responses in the more severe responses categories (since the current population is subjects with early and advanced PD unlike the data from De Novo cohort from the PPMI database which included mostly early-stage patients) for any of the items that are directly mapped. In contrast, for items that were categorized under indirect-mapping, new parameters reflecting only the reassignment of the probabilities were estimated to account for the qualitative differences between the two endpoints, while fixing all the Item Characteristic Curve (ICC) parameters to values obtained in the previously developed IRM. For instance, the mapping scheme (UPDRS ➔ MDS-UPDRS) for the item *leg agility* was described as:

- 0 (Normal) ➔ 0 (Normal)
- 1 (Mild) ➔ 1 (Slight) or 2 (Mild)
- 2 (Moderate) ➔ 2 (Mild) or 3 (Moderate)
- 3 (Severe) ➔3 (Moderate)
- 4 (Can barely perform the tasks) ➔4 (Severe)

Only the new parameters (FR2 and FR3) were estimated based on the reassignment of probabilities in the final adapted IRM:

2 $$ {\displaystyle \begin{array}{c}P\left({Y}_{ij}=0\right)=P0\\ {}P\left({Y}_{ij}=1\right)=P1+P2\bullet FR2\\ {}P\left({Y}_{ij}=2\right)=P2\bullet \left(1- FR2\right)+P3\bullet FR3\\ {}\begin{array}{l}P\left({Y}_{ij}=3\right)=P3\bullet \left(1- FR3\right)\\ {}P\left({Y}_{ij}=4\right)=P4\end{array}\end{array}} $$

The equations for the cumulative probabilities described in the previous section were also revised to incorporate the reassigned probabilities.

Lastly, for items that are exclusive to UPDRS (i.e. not part of MDS-UPDRS), ICC parameters were estimated in the same way as for the previous IRM (14).

### Baseline Modeling and Evaluation

The baseline (i.e. pre-dose) UPDRS data from early and advanced PD patients were used for adapting the previously developed IRM based on the workflow described above. This required estimating IRM parameters for items indirectly-mapped or unique to UPDRS and parameters linked to the patient populations (early *vs.* advanced PD), while fixing the other IRM parameters to the previously reported values. Therefore, in the first step, the new parameters for the indirect mapping items as well as all the ICC parameters for the items exclusive to UPDRS data were estimated, along with (1) the mean and variances of each of the latent variable’s distribution and (2) the *shift* - *S1* parameter for the SR latent variable, which reflects the lower disability for the *different* set combination of more disabled side and evaluated items (discussed in detail below).

At the end of the first step, the newly estimated parameters characterizing the reassignments for the items in the indirect mapping and ICCs for items exclusive to UPDRS data categories were fixed in all the subsequent estimation steps. Subsequently, in the second step, the mean and variance of each of the latent variable’s distribution was re-estimated using early PD patients as the reference population and implementing additional *shifts* - *S2, S3* for PR and NSR respectively; *S4* and *S5* for SR for advanced PD patient population. While the interpretation for *S2* and *S3* is relatively straightforward for PR and NSR respectively, it is a bit more nuanced for *S4/S5* for SR. The characterization of SR is unique in that it depends on (1) which side are the items being evaluated (e.g., right/left side) and (2) which subpopulation does the subject belong to: one whose more disabled side is the right/left side, estimated based on the mixture. Therefore, this leads to two sets of combinations:

(A) One whose right side is the more disabled side and the items evaluate the right side or one whose left side is the more disabled side and the items evaluate the left side – characterized as the *same* set combination of more disabled side and evaluated side;
(B) One whose right side is more disabled side and the items evaluate the left side or one whose right side is the more disabled side and the items evaluate the right side – characterized as *different* set combination of the more disabled side and evaluated side.

Expectedly so, the *same* set combination will have more disability (because they are the evaluated items for the side which is more disabled) than the *different* set combination. A shift parameter *S1*, is implemented to characterize the lower disability for the *different* set combination (constrained to be a negative number). It is worth noting that *S1* parameter exists for subjects in both the studies.

*S4* and *S5* represent the mean shifts in disability for the *same* and *different* sets of combinations respectively (described above) for subjects in the advanced PD study (169) using subjects in the early PD study (168) as reference. The shift parameters *S2, S3* (representing PR and NSR respectively) also account for mean shift in the disability for subjects in the advanced PD study using subjects in the early PD study as reference. Therefore, *S2-S5* parameters were constrained to be positive values because the subjects in study 169 have a higher disease severity (or higher values of the latent variable) than subjects in study 168. Lastly, correlation between the distributions of each of the latent variables at baseline within each study was implemented separately.

Simulation based diagnostics at each individual item level were explored as tools to evaluate the model performance. More specifically, the distributions of the mean of the simulated observed scores (based on 200 simulations from the final baseline model) were compared to the mean of all the observed scores for each item. Additionally, the correlations between the item responses were also explored by calculating the residuals the correlation matrix of the residuals as described previously (14).

### Placebo Modeling and Evaluation

The baseline data from in early and advanced PD patients, and the longitudinal data from the placebo arm in advanced PD patients were used to characterize the disease progression. The disease progression for each of the three latent variables was implemented as a linear model (21) with a function of time (in weeks) since baseline visit

3 $$ {D}_i(t)={D}_{i,0}+ Shift+{Slope}_i\ast t $$

*where D*_*i,0*_ is a subject-specific parameter comprised of a fixed effect (*θ*^*baseline*^*)* representing the mean of latent variable distribution and an additive random effect (*η*_*baseline*_) at baseline for early PD patients (reference population). Additionally, a *shift* term was implemented for subjects in advanced PD patients to reflect the higher disability (more severe disease status) of the subjects compared to the ones in the early PD study. The rate of disease progression was modeled as a fixed effect parameter (*θ*^*slope*^) associated with an additive random effect (*η*_*slope*_) resulting in a subject-specific *slope*_*i*_. Furthermore, the correlation structure between the random effects of the latent variable distribution at baseline and that of the slope for each latent variable was also explored, provided the model was stable and the estimation feasible.

Simulation based diagnostics at each individual item level were explored as tools to evaluate the model performance. More specifically, the distributions of the responses for each item over time were simulated (based on 200 simulations from the final placebo model) and compared with the observed distribution of responses for each item. Further simulation-based diagnostics were also performed by visual predictive checks (VPCs) using PsN tools (22). Monte Carlo simulations of 500 datasets were generated using the final models, and 95% prediction intervals were obtained around the median, the 2.5th and the 97.5th percentiles at the total score level and within each latent variable level, and compared with the same metrics calculated from the original data.

### Estimation Methods

All the analyses were performed using the software NONMEM version 7.3 (23). The parameter estimation was carried out using second-order conditional estimation with Laplacian approximation. Model selection between alternative nested models was based on likelihood ratio test of the obtained OFV at a significance level of *p* < 0.05 and Akaike Information Criteria (AIC) was used for evaluating non-nested models.

## Results

The first step to the model adaptation was categorization of the items based on the mapping mechanisms and this classification was based on the strategy outlined by Goetz *et al.* (6) and is shown in Table I along with the items assigned to each mechanism. About half of the items (55%) were mapped indirectly, while the items mapped directly (25%) and those unique to UPDRS (20%) comprised, in roughly equal proportions, the rest half. The adaptation workflow, conducted using the baseline UPDRS data of items 1–31 (parts I-III) included 24011 observations in a total of 550 patients with early and advanced PD.

**Table I:** Mapping Mechanisms Between UPDRS and MDS-UPDRS Scales

Mapping Mechanism	N^a^ (%)	Item number in UPDRS scale
Indirect	24 (55%)	1, 2, 6–8, 12, 15, 18, 22(5)^b^, 23(2)^b^, 24(2)^b^, 25(2)^b^, 26(2)^b^ 28–30,
Direct	11 (25%)	3–5, 9–11, 16, 17, 19, 27, 31
Only in UPDRS	9 (20%)	13, 14, 20(5)^b^, 21(2)^b^

^a^N represents the number of UPDRS items, and the percentage of items mapped in each category of mapping mechanism is shown in the parenthesis

^b^items evaluated for different parts and/or sides (e.g., left, right) of the body with the number of evaluations shown in parentheses

### Model Adaptation and Baseline Modeling

**Fig. 1:**
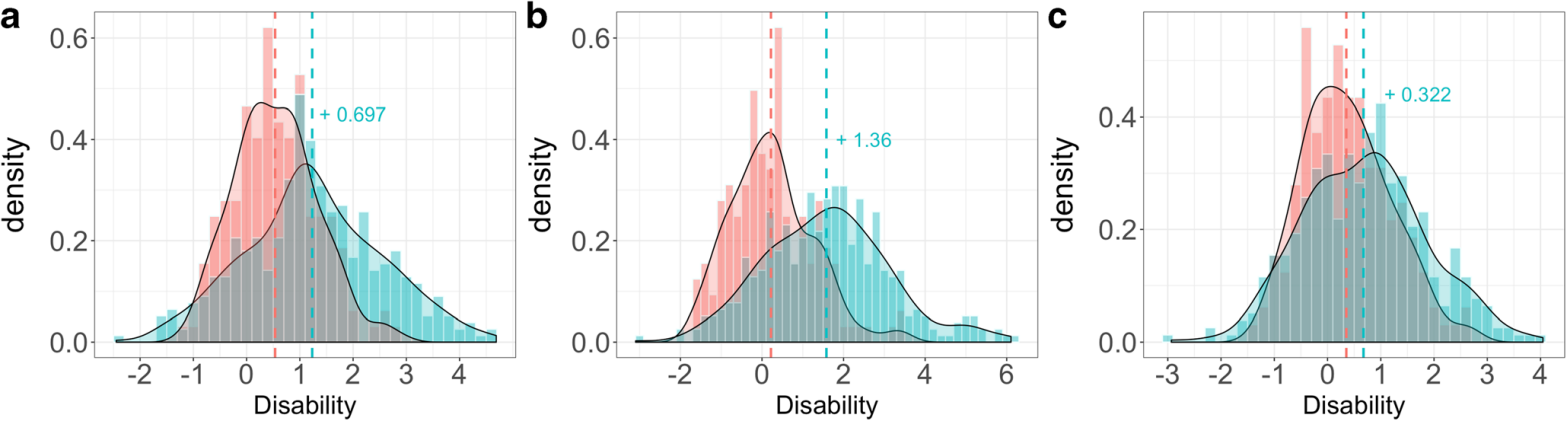
Graphical representation of latent variable distributions. The orange and blue colors represent the distribution in early and advanced subjects, respectively, for the latent variables accounting for the patient reported (PR), non-sided reported (NSR) and sided-reported (SR) items, respectively (panels **a, b**, and **c**) for same set combination of more disabled and evaluated items. The density curves (whose smoothness assumes kernel density estimation) are overlaid with the histograms of the respective studies, and the dashed lines represent the mean values for the subjects.

For the items that were mapped indirectly, the additional ICC parameters, which represent the reassignment of the probabilities for each response, were estimated successfully. Likewise, the IRM model parameters for the items that are unique to UPDRS were also estimated successfully. These parameters (shown in Appendix - I) were then fixed, and the mean and variance of each of the latent variable distributions at baseline were estimated using early PD patients as the reference population, along with the *shift* parameters for advanced PD patients and are shown in Table II and presented graphically in Fig. 1. The *shift* parameter was higher for NSR than PR. The variance in the distribution of the latent variables for advanced PD patients also followed similar trends and in general was higher than that estimated for early PD patients for all the three latent variables.

**Table II:** Mean and Variances of Latent Variable Distributions of Baseline Model

Parameter	Value for Early PD Patients Mean (Variance)	+ *Shift* = Value for Advanced PD Patients Mean (Variance)
Latent variable 1: *Patient reported items (1–17)*	0.535 (0.774)	+ 0.697 (*S2*^*a*^) = 1.23 (1.88)
Latent variable 2: *Non-sided items (18, 19, 20* ^*c*^ *, 22* ^*c*^ *, 27–31)*	0.219 (1.23)	+ 1.36 (*S3*^*a*^) = 1.58 (2.63)
Latent variable 3: *Sided items (20* ^*c*^ *, 21, 22* ^*c*^ *, 23–26)*	0.353 (0.821)	
*Shift* parameter reflecting the lower disability for the *different* set combination of more disabled side and evaluated items (*S1*^*b*^)		−1.94 (0.105)
*Shift* parameter for the *same* combination: items evaluated on the more disabled side (*S4*)		+ 0.322
*Shift* parameter for the *different* combination: items evaluated on the lower disabled side (*S5*)		+1.15

Number following ‘+’ is the shift parameter:

^a^*S2*, *S3*: Shift parameters for PR and NSR respectively for advanced PD patients, using early PD patients as reference

^b^*S1*: Shift parameter reflecting the lower disability for the different set combination of more disabled side and evaluated items (pertinent only to SR, and retained from the previously developed IRM model with multiple latent variables)

^c^Items 20 and 22 were evaluated for tremor at rest of face, lips, chin and rigidity of neck respectively, among the right and left sides of the body and therefore they appear in both the non-sided as well as sided latent variable

**Fig. 2:**
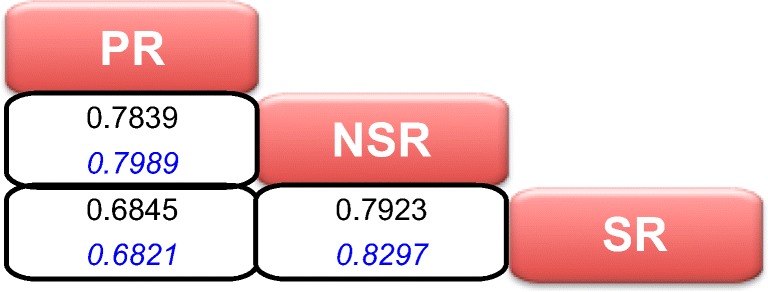
Correlation matrix of different latent variables. PR stands for patient reported, NSR for non-sided reported and SR for sided-reported. The values in black are the correlations for early PD and the values in blue are for advanced PD subjects.

Lastly, the correlation between each of the latent variables at baseline within each study is shown in Fig. 2. In general, correlation between PR-NSR and PR-SR was slightly higher than that estimated between NSR-SR. Additionally, the correlation structure of the latent variables at baseline seems to be consistent across the studies.

**Fig. 3:**
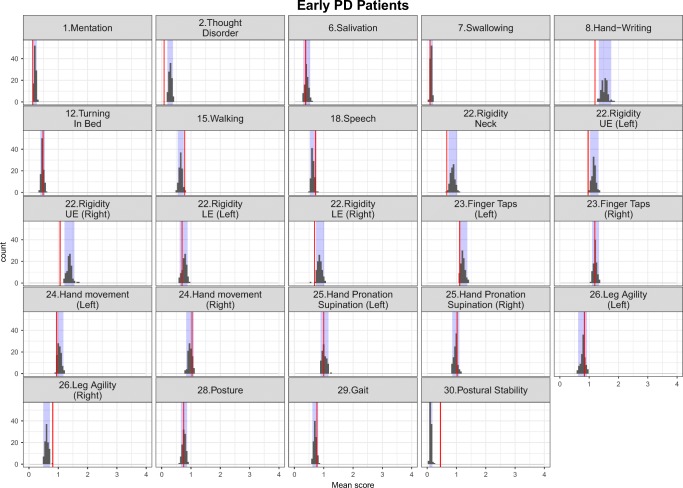

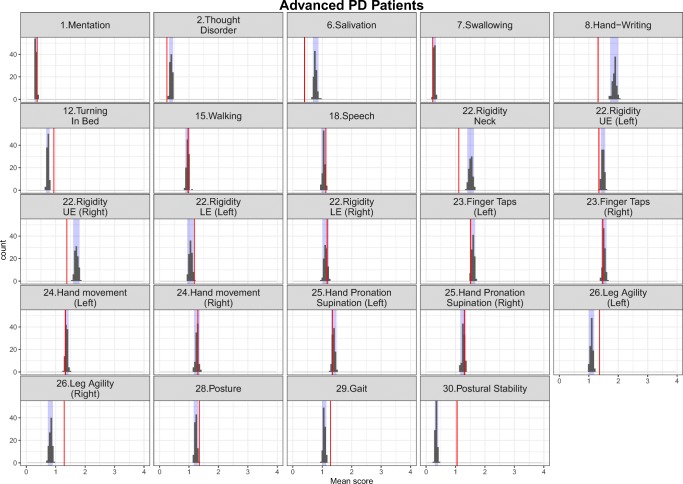
Simulation-based diagnostics for items mapped indirectly during the model adaptation using baseline data in early (top panel) and Advanced (bottom panel) PD patients. The red line is the calculated mean of all the observed scores for each item; black histograms represent the distribution of simulated mean scores (based on 200 simulations of the final baseline model) and purple shaded area shows 2.5th and 97.5th percentiles for each item based on the simulation. U/LE - Upper/Lower Extremity.

**Fig. 4:**
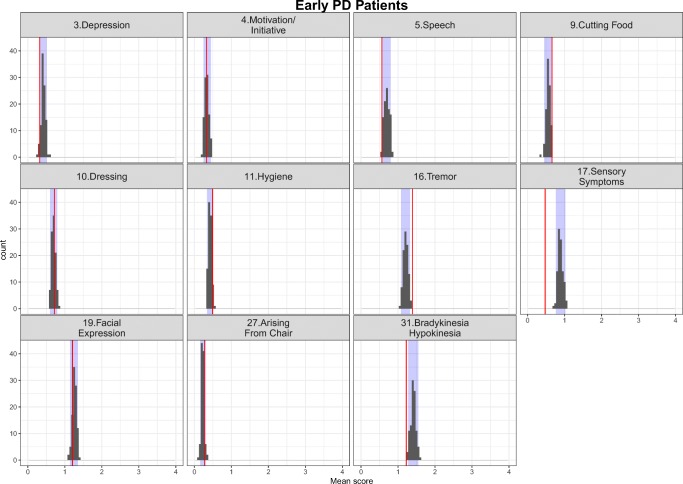

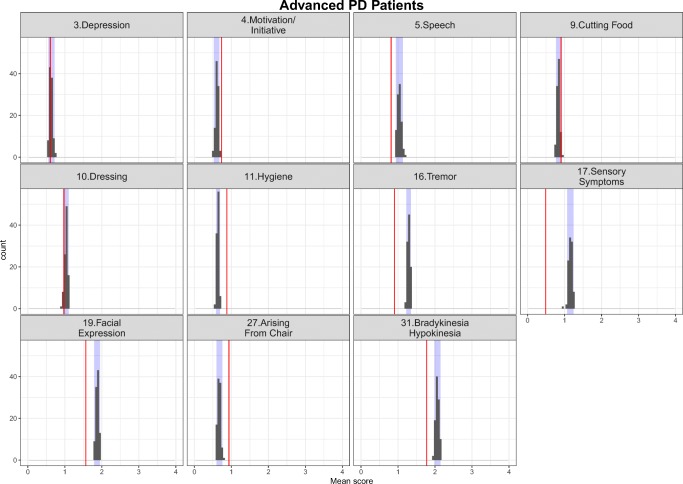
Simulation-based diagnostics for items mapped directly - model adaptation using baseline data in early (top panel) and Advanced (bottom panel) PD patients. The red line is the calculated mean of all the observed scores for each item; black histograms represent the distribution of simulated mean scores (based on 200 simulations of the final baseline model) and purple shaded area shows 2.5th and 97.5th percentiles for each item based on the simulation.

**Fig. 5:**
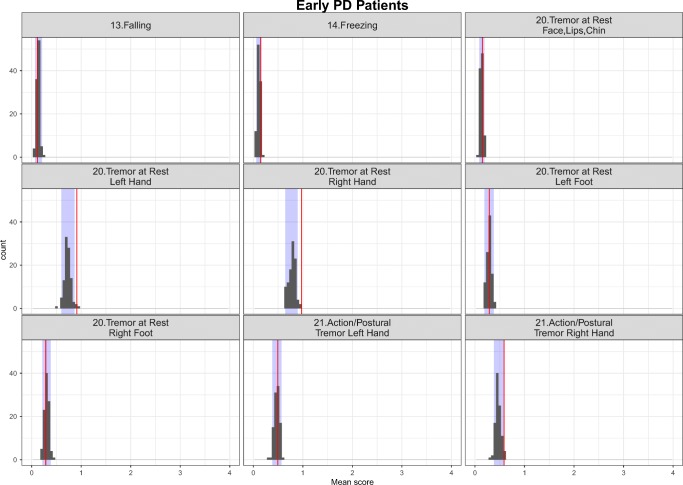

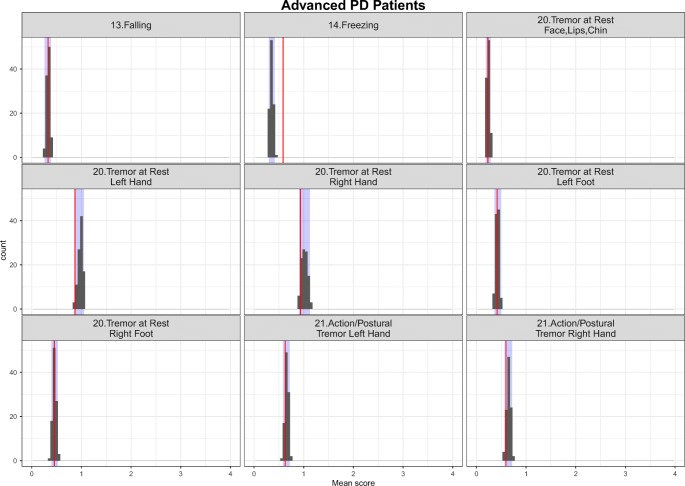
Simulation-based diagnostics for items exclusive to UPDRS scale - model adaptation using baseline data in early (top panel) and Advanced (bottom panel) PD patients. The red line is the calculated mean of all the observed scores for each item; black histograms represent the distribution of simulated mean scores (based on 200 simulations of the final baseline model) and purple shaded area shows 2.5th and 97.5th percentiles for each item based on the simulations.

The simulation-based diagnostics focusing on adequate replication of the mean score and based on 200 simulations for each category of the mapping mechanisms, indirect, direct and estimated exclusively based on UPDRS data are shown in Figs. 3, 4 and 5 stratified based on early and advanced PD patients respectively. While the agreement between the simulations and the observations was, as expected, reasonable for items whose IRM parameters were estimated exclusively based on UPDRS data, it varied from good to acceptable for directly and indirectly mapped items. All in all, although not all the IRM parameters were re-estimated, which allowed using prior knowledge and gained run-times, adequacy between the observed data and simulations was satisfactory. The correlation matrix of the residuals is provided together with the results for the simulation based diagnostics for each latent variable and total score stratified based on early and advanced PD patients in Appendix II. The syntax of the code used for the model adaptation and generating simulation-based diagnostics is provided in Appendix III.

### Placebo Modeling and Evaluation

**Fig. 6:**
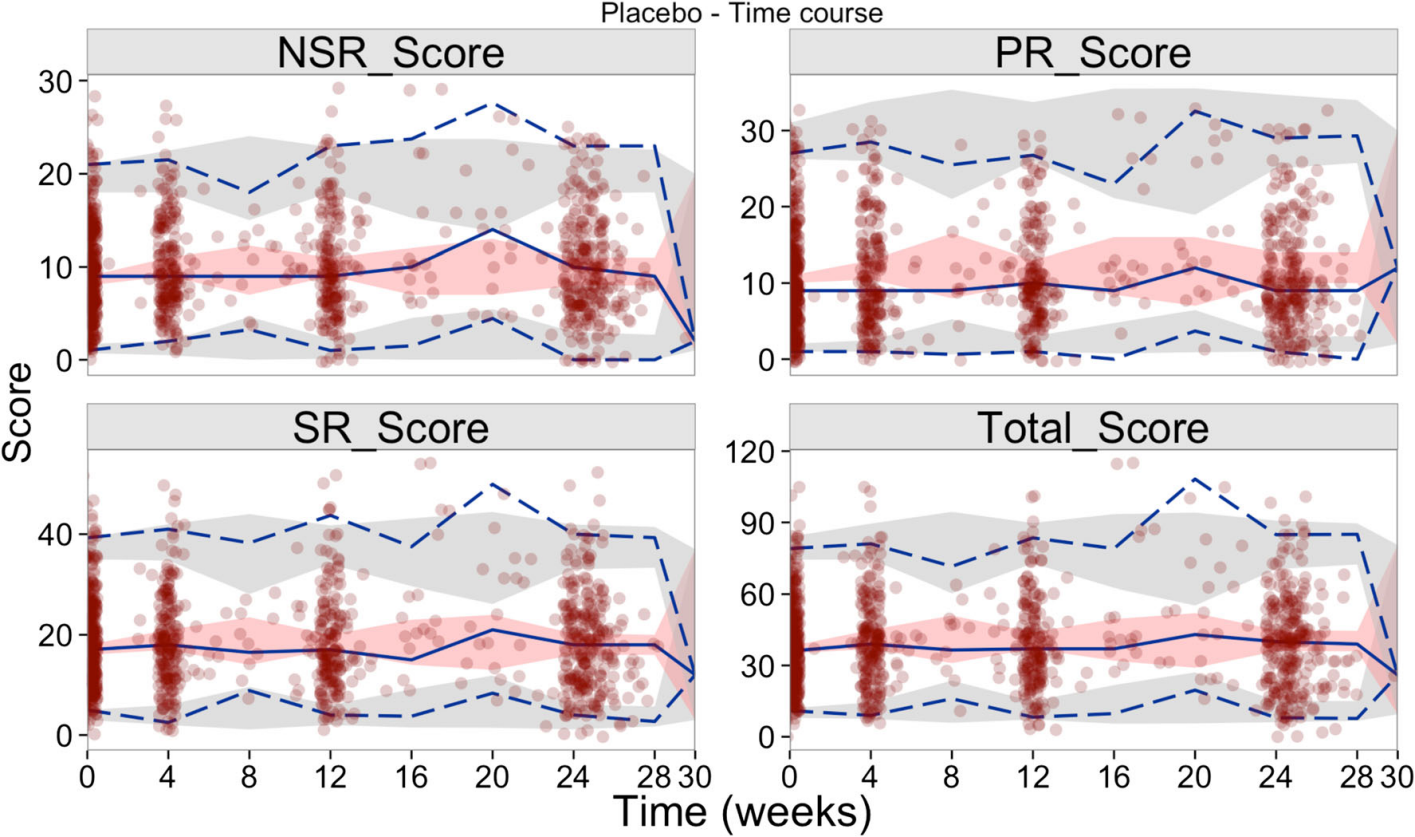
Simulation-based diagnostics for each of the three latent variables and the total score - model adaptation using longitudinal placebo data from Study 169. PR_Score, NSR_Score and SR_Score represent the sum of the scores of items characterized by the latent variables PR, NSR, SR latent variables respectively. The blue lines represent the median (solid), 2.5th and 97.5th quantiles (dashed) of the observed data (points) with the respective 95% confidence intervals (shaded areas) based on the final longitudinal model.

The longitudinal changes in the disease progression of each of the latent variables was explored based on the placebo arm in advanced PD population, which included 54783 observations in 551 individuals (also including baseline data). Of the three latent variables, only the SR for the same combination of the affected and evaluated side seemed to show a significant slope, −0.0094 per week, suggesting a placebo effect for this specific latent variable, i.e. an improvement in the disability over time. The slope for the other latent variables was not significant. Lastly, the VPCs for the final model, shown in Fig. 6 reflect the relatively flat nature of the disease progression when stratified based on the latent variables.

## Discussion

This work aimed at developing a quantitative framework, aiding to apply an existing item response model for the analysis of data from a different variant of the clinical endpoint and therefore, having the potential to improve the utilization of data. A previously developed longitudinal IRT model with multiple latent variables using MDS-UPDRS data from a de novo cohort of PD subjects (14) laid the foundation for the current work. The utility of such a framework was further explored using UPDRS clinical data at baseline and used to characterize the longitudinal changes of disease progression of advanced PD subjects in the placebo arm.

The previously developed model (14) was the first of its kind, quickly followed by another interesting characterization of the same data developed in parallel by Buatois *et al.* (24) While comparing the two MDS-UPDRS IRT models is beyond the scope of this paper, we note that the latter work grouped the items to support latent variables (tremor, motor and non-motor) that are different from ours. Traditional PD trial analyses are performed on the recorded total score, treated as a continuous variable, which shows some drawbacks when analyzing responses to items composed of categorical information. The IRT methodology appeases some of the shortcomings of the conventional analyses of PD trial data, since the biggest strength of this methodology lays in the context of integrating and leveraging knowledge from multiple data sources and different variants of a clinical endpoint as long as they are mapped on to the same latent variable(s) continuum. This can be achieved by collating UPDRS clinical data that had been collected in historical PD trials along with the more recent MDS-UPDRS version of the endpoint. Additionally, it should be noted that such a framework allows for inclusion of diverse patient populations, e.g., early *vs.* advanced PD subjects to explore for various features like disease progression, drug effects etc., which may not have been feasible when analyzed separately. Interestingly, according to the EMA: “The definitions […] on Parkinson’s disease e.g. ‘de novo Parkinson’s disease’, ‘early Parkinson’s disease’, ‘advanced Parkinson’s disease patient’ are considered working definitions based on clinical practice and are not intended to define exact and mutually exclusive patient populations.” (25)

Goetz *et al.* (6), compared in detail UPDRS and MDS-UPDRS based on a single large scale PD population and proposed a conceptual mapping of items between the two versions of the clinical PD endpoint. This formed the basis for categorizing the items in the questionnaire. While the directly mapped items did not require any additional estimation of parameters, for the indirectly mapped items, the ICCs generated from the previously developed IRT model were used as prior knowledge and additional parameters reflecting the reassignment of the response categories were successfully estimated. The degree of agreement varied depending on the nature of mapping, but overall, in the context of the noted differences in the mapping mechanisms (especially items under indirectly mapping) the model adaptation with the baseline appears to be generally adequate. It is also worth noting that, in general, for both directly and indirectly mapped items, there seems to be relatively higher degree of agreement between the observed and simulated data from in early and advanced PD patients. This is likely because the patients with early PD and the De Novo subjects (previously developed IRM) have similar disease severity than subjects with advanced PD. Although a high degree of agreement between the observed and predicted data is preferred for all items regardless of the mapping mechanisms, conceivably the construct of not only the two endpoint scales but also the description of the response categories mean that a perfectly coherent and unified scale may not be possible.

While re-estimating the IRM parameters is of interest, it is associated with significant costs in the run times, and ignores prior knowledge. The shift parameter *S1* reflecting the lower disability for the *different* set combination of the more disabled side and evaluated items estimated in the final baseline model was consistent (mean [variance]: 1.94 [0.105]) with the shift parameter characterizing the lower disability of the initially better side from the previous IRM (2.11 [0.60]), reflecting the consistency of the structural model, especially in terms of the SR latent variable, in handling populations with different disease severity (early and advanced PD subjects in this work *vs.* De Novo subjects in previous work).

The shifts in the mean were positive for all the latent variables in study 169, which is expected knowing the more advanced PD status of the patients enrolled in this study compared to the other one. Additionally, the variances for these latent variable distributions were higher in the advanced PD study suggesting that the patient population is also more heterogeneous than in the early PD study. Interestingly, the correlations between the different latent variables within each study population still seemed to be rather consistent. Most of the values are 0.7 or above suggesting consistency of the contribution of each variable towards overall disease severity, which also seemed consistent in across early and advanced PD populations.

It is worth noting that the correlations between the latent variables in early and advanced PD populations seem to be higher than those observed in the previous IRM based on De Novo patient population based on the PPMI database. This is likely because the more severe the disease in the population, i.e. advanced PD subjects *vs.* early *vs.* De Novo, the more correlated the symptoms are expected, especially according to a scale originally developed based on patients with a more severe disease status.

The time course of the placebo treatment in advanced PD patients, which conceivably is a composite of natural disease progression and placebo treatment effects, showed a significant slope for only one of the latent variables, SR with the *same* set combination of the more disabled and evaluated side. The negative slope for SR suggests that for the items being evaluated on the more disabled side at baseline (either right/left side based on the mixture proportion) will progress slower. The plausible explanation for such an occurrence could be because the placebo arm was not truly placebo, as the patients had advanced PD and were allowed to have a background therapy of levadopa to help alleviate the symptoms. Consequently, the background therapy could have masked the disease progression for the other latent variables potentially. More importantly, longer trial duration is likely needed to allow reliable estimation of a significant slope over time.

The lack of detectable slope change for the other latent variables was likely due to the combination of short treatment trial duration and small sample size, in the context of the slow progression. Drug effects can be added on the base IRT model, and would typically be entered on the latent variable, as per the philosophy of the theory. In the case of several latent variables, several drug effects can be added, with different shapes, sizes and interpretations. They would commonly consist in mixed effects and can potentially share parameters including random effects. Examples of drug effect implementations on IRT models exist for a variety of diseases: Alzheimer’s disease, schizophrenia, multiple sclerosis, (11–13,26,27). Drug effects can also be tested on the item specific parameters if relevant.

Others have reported bridging the two versions of the instrument at total-score level, Goetz *et al.* (6) also carried out a validation program for the MDS-UPDRS by collecting both UPDRS and MDS-UPDRS data in about 875 patients with PD (majority with mild to moderate severity of PD). As noted earlier, given the structural and conceptual similarities of Parts II and III, Goetz *et al.* hypothesized to be able to calculate formulae for conversion of UPDRS total scores to MDS-UPDRS total scores for Parts II and III with high reliability (15). The validation data was used for this exercise and the authors showed that there was a high degree of concordance between the actual MDS-UPDRS total scores and those estimated from the UPDRS in Parts II and III (predicted score is expected to be within 9 points 95% of the time), suggesting the high accuracy of the conversion methodology. Merello *et al.* (17) also showed excellent correlation between Part III total score of UPDRS and MDS-UPDRS following an acute dose of levadopa/carbidopa 250/25 mg in total of 64 subjects (clarification and/or characterization of response to levadopa in 25 patients, and De Novo diagnosis in 39 patients). Additionally, they also noted that the prediction of long-term levodopa response using a 24% change in MDS-UPDRS Part III total score was equivalent to predication of long-term levadopa response using a 30% change in UPDRS Part III total score. However, their study did not explore conversion methodology between the two rating scales. Lastly, Hentz *et al.* (16) explored a simplified method for converting UPDRS Part III total to MDS-UPDRS Part III total score in a total of 38 patients with PD. They reported that simply adding 7 points to UPDRS Part III total score provided a good approximation of the MDS-UPDRS Part III total score. The authors noted that while their simplified conversion method may be more useful in a routine clinical setting to monitor and track the progression of motor signs with PD patients, the formulae reported by Goetz *et al.* (15) had the best available accuracy.

In contrast, IRT offers a model-based framework with the potential for a joint analysis of clinical studies by exploring the mapping mechanisms to bridge between different versions of the clinical endpoint as proposed in the current work. This can result in improved utilization of the data, not only at the total score level but also at the individual item level, representing more informative use of trial data.

In summary, we applied the model that was developed using data of MDS-UPDRS from one trial in early-stage patients to data of UPDRS (a different version of the instrument) from two other trials, one of which was in advanced-stage patients. To a certain extent, this resembled an external model validation with high hurdles - using a somewhat different endpoint from a partly different population. Nonetheless, we tested the approach due to the IRT methodology’s ability of more informative use of data by drawing information directly from item level, hence the potential for better bridging between endpoints and trial population. In this report, we focused on modelling baseline and placebo data. Modelling of drug effect is ongoing and will be reported separately. We hope that the addition of these findings to the emerging experience in applying IRT to clinical trial analysis would encourage broader exploration of the utility of this methodology.

## Conclusion

A model-based quantitative framework was developed through the adaptation of an Item Response Model between different variants of the clinical endpoint. This work allows effective use of historical knowledge and enables data integration. The model adapted in this work was used to integrate clinical endpoint data from Parkinson’s disease patients in early and advanced severity and to describe the time course of placebo treatment in a clinical trial.

## Electronic supplementary material

ESM 1 (PDF 268 kb)

ESM 2 (PDF 796 kb)

ESM 3 (PDF 565 kb)
