# Supplementary material for: Item Response Model Adaptation for Analyzing Data from Different Versions of Parkinson’s Disease Rating Scales
Source: Pharm Res. 2019 Jul 17;36(9):135. doi: 10.1007/s11095-019-2668-6 (PMC6647468; doi:10.1007/s11095-019-2668-6)
Supplement: Supplementary file 1 — (PDF 268 kb) [file 11095_2019_2668_MOESM1_ESM.pdf]

Appendix I – Model adaptation workflow

This appendix contains all the ICC parameter estimates for model adaptation based on baseline data from study 168 and study 169:

1 Intellectual Impairment in UPDRS: Cognitive Impairment in MDS-UPDRS:

Indirect mapping mechanism: UPDRS ➔MDS-UPDRS
0 ➔0, 1 ➔2, 2 ➔3, 3 ➔4, 4 ➔4

$$P(Y_{ij} = 0) = P0 + P1*FR1$$
$$P(Y_{ij} = 1) = P2 + P1*(1 - FR1)$$
$$P(Y_{ij} = 2) = P3$$
$$P(Y_{ij} = 3) = P4*FR4$$
$$P(Y_{ij} = 4) = P4*(1 - FR4)$$

**Item -*j***	**Category**	a_j	$b_{j,k}$	***FR***
**1**	> 1	0.80	1.4	*FR1 = 0.49*
	> 2		4.2	*FR4 = 1*
	> 3		6.7	
	= 4		57	

2 Thought Disorder in UPDRS: Hallucinations and Psychosis in MDS-UPDRS

Indirect mapping mechanism: UPDRS ➔MDS-UPDRS
0➔0, 1➔0, 2➔1|2, 3➔3, 4➔4

$$P(Y_{ij} = 0) = P0*FR0$$
$$P(Y_{ij} = 1) = P0*(1 - FR0)$$
$$P(Y_{ij} = 2) = P1 + P2$$
$$P(Y_{ij} = 3) = P3$$
$$P(Y_{ij} = 4) = P4$$

**Item -*j***	**Category**	a_j	$b_{j,k}$	***FR***
**2**	> 1	0.72	4.4	*FR0 = 0.84*
	> 2		8.3	
	> 3		58	
	= 4		108	

3 Depression in UPDRS: Depressed mood in MDS-UPDRS

Direct mapping

**Item -*j***	**Category**	a_j	$b_{j,k}$
**3**	> 1	0.76	1.6
	> 2		4.0
	> 3		6.2
	= 4		56

4 Motivation and Initiative in UPDRS: Apathy in MDS – UPDRS

Direct mapping

**Item -*j***	**Category**	a_j	$b_{j,k}$
**4**	> 1	1.1	1.7
	> 2		3.3
	> 3		5.5
	= 4		55

5 Speech in UPDRS: Speech in MDS-UPDRS

Direct Mapping

**Item -*j***	**Category**	a_j	$b_{j,k}$
**5**	> 1	1.1	0.65
	> 2		2.1
	> 3		4.2
	= 4		54

6 Salivation in UPDRS: Saliva and Drooling in MDS-UPDRS

Indirect mapping mechanism: UPDRS ➔MDS-UPDRS
0➔0, 1➔2, 2➔3, 3➔3, 4➔4

$$P(Y_{ij} = 0) = P0 + P1*FR1$$
$$P(Y_{ij} = 1) = P1*(1 - FR1) + P2$$
$$P(Y_{ij} = 2) = P3*FR3$$
$$P(Y_{ij} = 3) = P3*(1 - FR3)$$
$$P(Y_{ij} = 4) = P4$$

**Item -*j***	**Category**	***a*$_j$**	***b*$_{j,k}$**	***FR***
**6**	> 1	1.0	0.70	*FR1 = 1*
	> 2		1.7	*FR3 = 0.76*
	> 3		3.0	
	= 4		5.2	

7 Swallowing in UPDRS: Chewing and Swallowing in MDS-UPDRS

Indirect mapping mechanism: UPDRS ➔MDS-UPDRS
0 ➔0, 1➔3, 2➔3, 3➔2, 4➔4

$$P(Y_{ij} = 0) = P0 + P1*FR1$$
$$P(Y_{ij} = 1) = P1*(1 - FR1) + P3*FR3$$
$$P(Y_{ij} = 2) = P3*(1 - FR3)$$
$$P(Y_{ij} = 3) = P2$$
$$P(Y_{ij} = 4) = P4$$

**Item -*j***	**Category**	***a*$_j$**	***b*$_{j,k}$**	***FR***
**7**	> 1	1.0	1.9	*FR1 = 0.57*
	> 2		4.8	*FR3 = 0*
	> 3		5.6	
	= 4		56	

8 Handwriting in UPDRS: Handwriting in MDS-UPDRS

Indirect mapping mechanism: UPDRS ➔MDS-UPDRS
0➔0, 1➔1, 2➔1, 3➔2|3, 4➔4

$$P(Y_{ij} = 0) = P0$$
$$P(Y_{ij} = 1) = P1*FR1$$
$$P(Y_{ij} = 2) = P1*(1 - FR1)$$
$$P(Y_{ij} = 3) = P2 + P3$$
$$P(Y_{ij} = 4) = P4$$

Item -*j*	Category	a_j	$b_{j,k}$	*FR*
**8**	> 1	0.90	-0.65	*FR1 = 0.64*
	> 2		1.5	
	> 3		3.4	
	= 4		5.7	

9 Cutting Food and Handling Utensils in UPDRS: Eating Tasks in MDS-UPDRS

Direct mapping mechanism

Item -*j*	Category	a_j	$b_{j,k}$
**10**	> 1	1.3	0.63
	> 2		2.8
	> 3		6.5
	= 4		8.5

10 Dressing in UPDRS: Dressing in MDS-UPDRS

Direct mapping mechanism

Item -*j*	Category	a_j	$b_{j,k}$
10	> 1	1.7	0.25
	> 2		2.2
	> 3		4.7
	= 4		6.6

11 Hygiene in UPDRS: Hygiene in MDS-UPDRS

Direct mapping mechanism

Item -*j*	Category	a_j	$b_{j,k}$
12	> 1	1.3	0.88
	> 2		4.5
	> 3		5.9
	= 4		56

12 Turning in Bed and Adjusting Clothes in UPDRS: Turning in Bed in MDS-UPDRS

Indirect mapping mechanism: UPDRS ➔MDS-UPDRS
0➔0, 1➔1, 2➔2|3, 3➔3|4, 4➔4

$$P(Y_{ij} = 0) = P0$$
$$P(Y_{ij} = 1) = P1$$
$$P(Y_{ij} = 2) = P2 + P3*FR3$$
$$P(Y_{ij} = 3) = P3*(1 - FR3) + P4*FR4$$
$$P(Y_{ij} = 4) = P4*(1- FR4)$$

Item -*j*	Category	a_j	$b_{j,k}$	*FR*
12	> 1	1.5	0.78	*FR3 = 0*
	> 2		3.4	*FR4 = 0*
	> 3		5.6	
	= 4		7.5	

13 Falling in UPDRS

[In UPDRS only]

Item -*j*	Category	a_j	$b_{j,k}$
**14**	> 1	1.3	2.5
	> 2		4.2
	> 3		5.9
	= 4		56

14 Freezing When Walking in UPDRS: Freezing in MDS-UPDRS

*Unique parameterization: for probabilities of 2, 3, 4

$$P(Y_{ij} = 0) = P0$$
$$P(Y_{ij} = 1) = P1$$
$$P(Y_{ij} = 2) = NP2$$
$$P(Y_{ij} = 3) = NP3$$
$$P(Y_{ij} = 4) = NP4$$

Item -*j*	Category	a_j	$b_{j,k}$
**15**	> 1	1.5	2.4
	> 2		3.8
	> 3		5.3
	= 4		7.1

15 <u>Walking in UPDRS: Walking and Balance in MDS-UPDRS</u>

Indirect mapping mechanism: UPDRS ➔MDS-UPDRS
0➔0, 1➔1, 2➔1|2, 3➔3|4, 4➔4

$$P(Y_{ij} = 0) = P0$$
$$P(Y_{ij} = 1) = P1*FR1$$
$$P(Y_{ij} = 2) = P2 + P1*(1 - FR1)$$
$$P(Y_{ij} = 3) = P3 + P4*FR4$$
$$P(Y_{ij} = 4) = P4*(1 - FR4)$$

Item -*j*	Category	a_j	$b_{j,k}$	*FR*
15	> 1	1.5	0.38	*FR1 = 0.94*
	> 2		3.0	*FR4 = 1*
	> 3		3.9	
	= 4		54	

16 <u>Tremor in UPDRS: Tremor in MDS-UPDRS</u>

Direct mapping mechanism

Item -*j*	Category	a_j	$b_{j,k}$
16	> 1	0.29	-6.2
	> 2		3.7
	> 3		11
	= 4		19

17 <u>Sensory Complaints related to Parkinson's Disease in UPDRS: Pain and Other Sensations in MDS-UPRDS</u>

Direct mapping mechanism

Item -*j*	Category	a_j	$b_{j,k}$
17	> 1	0.85	-0.11
	> 2		2.4
	> 3		3.9
	= 4		54

18 Speech (Motor Examination) in UPDRS: Speech (Motor Examination) in MDS-UPDRS

Indirect mapping mechanism: UPDRS ➔MDS-UPDRS
0➔0, 1➔1, 2➔2, 3➔3|4, 4➔4

$$P(Y_{ij} = 0) = P0$$
$$P(Y_{ij} = 1) = P1$$
$$P(Y_{ij} = 2) = P2$$
$$P(Y_{ij} = 3) = P3 + P4*FR4$$
$$P(Y_{ij} = 4) = P4*(1- FR4)$$

**Item -*j***	**Category**	a_j	$b_{j,k}$	***FR***
**19**	> 1	1.1	0.040	*FR4 = 0*
	> 2		3.0	
	> 3		6.0	
	= 4		9.0	

19 Facial Expression in UPDRS: Facial Expression in MDS-UPDRS

Direct mapping mechanism

**Item -*j***	**Category**	a_j	$b_{j,k}$
**19**	> 1	1.6	-1.6
	> 2		0.84
	> 3		3.0
	= 4		4.9

20 Tremor at Rest in UPDRS

Unique to UPDRS: As there is no parallelism to Rest Tremor Amplitude in MDS-UPDRS

(1) Face, Lips and Chin

Item -*j*	Category	a_j	$b_{j,k}$
20 (1)	> 1	0.42	5.2
	> 2		9.8
	> 3		17
	= 4		67

(2) Left Upper Extremity

Item -*j*	Category	a_j	$b_{j,k}$
20 (2)	> 1	0.72	-0.62
	> 2		1.5
	> 3		4.0
	= 4		9.2

(3) Right Upper Extremity

Item -*j*	Category	a_j	$b_{j,k}$
20 (3)	> 1	0.76	-0.49
	> 2		1.4
	> 3		3.7
	= 4		7.6

(4) Left Lower Extremity

Item -*j*	Category	a_j	$b_{j,k}$
20 (4)	> 1	0.80	1.3
	> 2		3.4
	> 3		6.2
	= 4		56

(5) Right Lower Extremity

Item -*j*	Category	a_j	$b_{j,k}$
20 (5)	> 1	0.90	1.1
	> 2		3.1
	> 3		5.8
	= 4		56

21 Action/Postural Tremor in UPDRS

Unique to UPDRS: As there is no parallelism to 'Postural Tremor' and 'Kinetic Tremor' in MDS-UPDRS

(1) Left Hand

Item -j	Category	a_j	$b_{j,k}$
21 (1)	> 1	0.77	0.12
	> 2		2.9
	> 3		5.4
	= 4		55

(2) Right Hand

Item -j	Category	a_j	$b_{j,k}$
21 (2)	> 1	0.84	0.31
	> 2		2.8
	> 3		4.9
	= 4		7.5

22 <u>Rigidity in UPDRS: Rigidity in MDS-UPDRS</u>

Indirect mapping mechanism: UPDRS ➔MDS-UPDRS
0➔0, 1➔1, 2➔2, 3➔2, 4➔3|4

$$P(Y_{ij} = 0) = P0$$
$$P(Y_{ij} = 1) = P1$$
$$P(Y_{ij} = 2) = P2*FR2$$
$$P(Y_{ij} = 3) = P2*(1- FR2) + P4*FR4$$
$$P(Y_{ij} = 4) = P3 + P4*(1- FR4)$$

(1) Neck

Item -*j*	Category	a_j	$b_{j,k}$	*FR*
22 (1)	> 1	1.0	-0.023	*FR2 = 0.84*
	> 2		1.6	*FR4 = 1*
	> 3		4.6	
	= 4		7.9	

(2) Left Upper Extremity

Item -*j*	Category	a_j	$b_{j,k}$	*FR*
22 (2)	> 1	1.2	-2.0	*FR2 = 1*
	> 2		-0.14	*FR4 = 0.95*
	> 3		2.6	
	= 4		2.6	

Indirect mapping mechanism: UPDRS ➔MDS-UPDRS
0➔0, 1➔1, 2➔2, 3➔2, 4➔3|4

$$P(Y_{ij} = 0) = P0$$
$$P(Y_{ij} = 1) = P1$$
$$P(Y_{ij} = 2) = P2*FR2$$
$$P(Y_{ij} = 3) = P2*(1- FR2) + P4*FR4$$
$$P(Y_{ij} = 4) = P3 + P4*(1- FR4)$$

(3) Right Upper Extremity

Item -*j*	Category	a_j	$b_{j,k}$	*FR*
22 (3)	> 1	1.2	-2.1	*FR2 = 0.82*
	> 2		-0.22	*FR4 = 1*
	> 3		2.8	
	= 4		6.1	

(4) Left Lower Extremity

Item -*j*	Category	a_j	$b_{j,k}$	*FR*
22 (4)	> 1	0.93	-0.73	*FR2 = 1*
	> 2		0.93	*FR4 = 0.87*
	> 3		3.6	
	= 4		3.6	

(5) Right Lower Extremity

Item -*j*	Category	a_j	$b_{j,k}$	*FR*
22 (5)	> 1	0.85	-0.70	*FR2 = 0.79*
	> 2		1.2	*FR4 = 1*
	> 3		4.4	
	= 4		9.4	

23 Finger Taps in UPDRS: Finger Taps in MDS-UPDRS

Indirect mapping mechanism: UPDRS ➔MDS-UPDRS
0➔0, 1➔1|2, 2➔2|3, 3➔3, 4➔4

$$P(Y_{ij} = 0) = P0$$
$$P(Y_{ij} = 1) = P1 + P2*FR2$$
$$P(Y_{ij} = 2) = P2*(1- FR2) + P3*FR3$$
$$P(Y_{ij} = 3) = P3*(1- FR3)$$
$$P(Y_{ij} = 4) = P4$$

(1) Left Hand

Item -*j*	Category	a_j	$b_{j,k}$	*FR*
**23 (1)**	> 1	1.8	-2.1	*FR2 = 0.20*
	> 2		-0.39	*FR3 = 0.45*
	> 3		1.3	
	= 4		3.3	

(2) Right Hand

Item -*j*	Category	a_j	$b_{j,k}$	*FR*
**23 (2)**	> 1	1.8	-1.8	*FR2 = 0.099*
	> 2		0.013	*FR3 = 0.41*
	> 3		1.6	
	= 4		3.6	

24 Hand Movements in UPDRS: Hand Movements in MDS-UPDRS

Indirect mapping mechanism: UPDRS ➔MDS-UPDRS
0➔0, 1➔1|2, 2➔2|3, 3➔3, 4➔4

$$P(Y_{ij} = 0) = P0$$
$$P(Y_{ij} = 1) = P1 + P2*FR2$$
$$P(Y_{ij} = 2) = P2*(1- FR2) + P3*FR3$$
$$P(Y_{ij} = 3) = P3*(1- FR3)$$
$$P(Y_{ij} = 4) = P4$$

(1) Left Hand

Item -*j*	Category	a_j	$b_{j,k}$	*FR*
**24 (1)**	> 1	1.7	-1.7	*FR2 = 0.23*
	> 2		-0.034	*FR3 = 0.52*
	> 3		1.7	
	= 4		52	

(2) Right Hand

Item -*j*	Category	a_j	$b_{j,k}$	*FR*
**24 (2)**	> 1	1.9	-1.3	*FR2 = 0.066*
	> 2		0.45	*FR3 = 0.55*
	> 3		2.0	
	= 4		52	

25 Hand Pronation and Supination in UPDRS: Hand Pronation and Supination in MDS-UPDRS

Indirect mapping mechanism: UPDRS ➔MDS-UPDRS
0➔0, 1➔1|2, 2➔2|3, 3➔3, 4➔4

$$P(Y_{ij} = 0) = P0$$
$$P(Y_{ij} = 1) = P1 + P2*FR2$$
$$P(Y_{ij} = 2) = P2*(1- FR2) + P3*FR3$$
$$P(Y_{ij} = 3) = P3*(1- FR3)$$
$$P(Y_{ij} = 4) = P4$$

(1) Left Hand

Item -*j*	Category	a_j	$b_{j,k}$	*FR*
**25 (1)**	> 1	1.7	-1.6	*FR2 = 0.23*
	> 2		0.052	*FR3 = 0.44*
	> 3		1.6	
	= 4		3.5	

(2) Right Hand

Item -*j*	Category	a_j	$b_{j,k}$	*FR*
**25 (2)**	> 1	1.6	-1.3	*FR2 = 0.085*
	> 2		0.53	*FR3 = 0.28*
	> 3		2.4	
	= 4		4.5	

26 Leg Agility in UPDRS: Leg Agility in MDS-UPDRS

Indirect mapping mechanism: UPDRS ➔MDS-UPDRS
0➔0, 1➔1|2, 2➔2|3, 3➔3, 4➔4

$$P(Y_{ij} = 0) = P0$$
$$P(Y_{ij} = 1) = P1 + P2*FR2$$
$$P(Y_{ij} = 2) = P2*(1- FR2) + P3*FR3$$
$$P(Y_{ij} = 3) = P3*(1- FR3)$$
$$P(Y_{ij} = 4) = P4$$

(1) Left Leg

Item -*j*	Category	a_j	$b_{j,k}$	*FR*
26 (1)	> 1	1.3	-0.97	*FR2 = 0*
	> 2		0.92	*FR3 = 0*
	> 3		3.0	
	= 4		6.4	

(2) Right Leg

Item -*j*	Category	a_j	$b_{j,k}$	*FR*
26 (2)	> 1	1.0	-0.43	*FR2 = 0*
	> 2		2.1	*FR3 = 0*
	> 3		4.5	
	= 4		6.6	

27 Arising From Chair in UPDRS: Arising From Chair in MDS-UPDRS

Direct mapping mechanism:

Item -*j*	Category	a_j	$b_{j,k}$
**27**	> 1	1.1	1.9
	> 2		3.9
	> 3		5.3
	= 4		6.7

28 Posture in UPDRS: Posture in MDS-UPDRS

Indirect mapping mechanism: UPDRS ➔MDS-UPDRS
0➔0, 1➔1, 2➔2|3, 3➔4, 4➔4

$$P(Y_{ij} = 0) = P0$$
$$P(Y_{ij} = 1) = P1$$
$$P(Y_{ij} = 2) = P2 + P3$$
$$P(Y_{ij} = 3) = P4*FR4$$
$$P(Y_{ij} = 4) = P4*(1- FR4)$$

Item -*j*	Category	a_j	$b_{j,k}$	*FR*
**28**	> 1	1.3	-0.27	*FR4 = 0.92*
	> 2		2.1	
	> 3		4.2	
	= 4		6.6	

29 Gait in UPDRS: Gait in MDS-UPDRS

Indirect mapping mechanism: UPDRS ➔MDS-UPDRS
0➔0, 1➔1, 2➔2, 3➔3|4, 4➔4

$$P(Y_{ij} = 0) = P0$$
$$P(Y_{ij} = 1) = P1$$
$$P(Y_{ij} = 2) = P2$$
$$P(Y_{ij} = 3) = P3 + P4*FR4$$
$$P(Y_{ij} = 4) = P4*(1- FR4)$$

**Item -*j***	**Category**	a_j	$b_{j,k}$	***FR***
**29**	> 1	0.92	-0.53	*FR4 = 0*
	> 2		3.5	
	> 3		6.0	
	= 4		8.4	

30 Postural Stability in UPDRS: Postural Stability in MDS-UPDRS

Indirect mapping mechanism: UPDRS ➔MDS-UPDRS
0➔0, 1➔1|2, 2➔3, 3➔4, 4➔4

$$P(Y_{ij} = 0) = P0$$
$$P(Y_{ij} = 1) = P1 + P2$$
$$P(Y_{ij} = 2) = P3$$
$$P(Y_{ij} = 3) = P4*FR4$$
$$P(Y_{ij} = 4) = P4*(1- FR4)$$

**Item -*j***	**Category**	a_j	$b_{j,k}$	***FR***
**29**	> 1	0.80	3.2	*FR4 = 0.93*
	> 2		4.8	
	> 3		5.8	
	= 4		8.9	

31 Body Bradykinesia and Hypokinesia in UPDRS: Global Spontaneity of Movement in MDS-UPDRS

Direct mapping mechanism

Item -*j*	Category	a_j	$b_{j,k}$
32	> 1	1.8	-1.5
	> 2		0.41
	> 3		2.2
	= 4		5.8
