# Supplementary material for: Item Response Model Adaptation for Analyzing Data from Different Versions of Parkinson’s Disease Rating Scales
Source: Pharm Res. 2019 Jul 17;36(9):135. doi: 10.1007/s11095-019-2668-6 (PMC6647468; doi:10.1007/s11095-019-2668-6)

Appendix – II Additional Graphs and Diagnostics

This appendix contains additional graphs for simulation-based diagnostics

Figure 1Correlation between the items: Correlation matrix of residuals in early PDsubjects

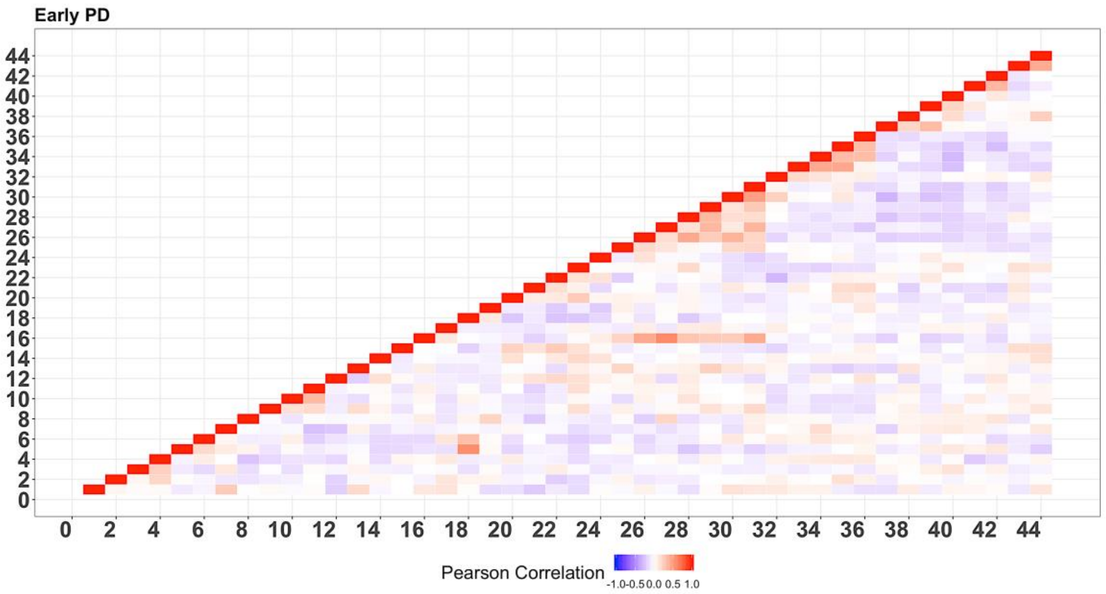

Figure 2Correlation between the items: Correlation matrix of residuals in advanced PDsubjects

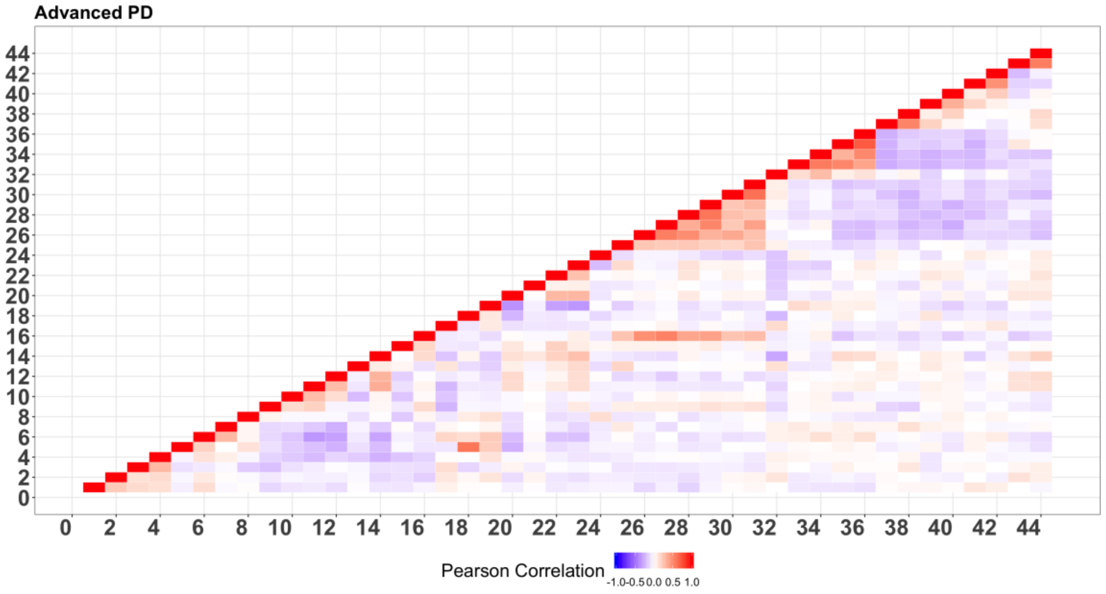

Note 1: Please refer to our earlier work for more information on how the residuals were calculated [1]

[1] *Gottipati, G., Karlsson, M. O., & Plan, E. L. (2017). Modeling a composite score in Parkinson's disease using item response theory. The AAPS journal, 19(3), 837-845*

Note 2: Please refer to the model adaptation code for the specific item number and what they denote: While items 1-19 as listed in the model adaptation code represent 1-19 items in this plot, items 27-30 represent 20-24 respectively and items 200-219 represent 25-44 respectively. This notation was adapted to facilitate the continuity in plotting the correlation matrix

Figure 3Posterior predictive checkfor the total mean score in early PDsubjects, as well as stratified by latent variable(PR stands for patient reported, NSR for non-sided reported and SR for sided-reported)

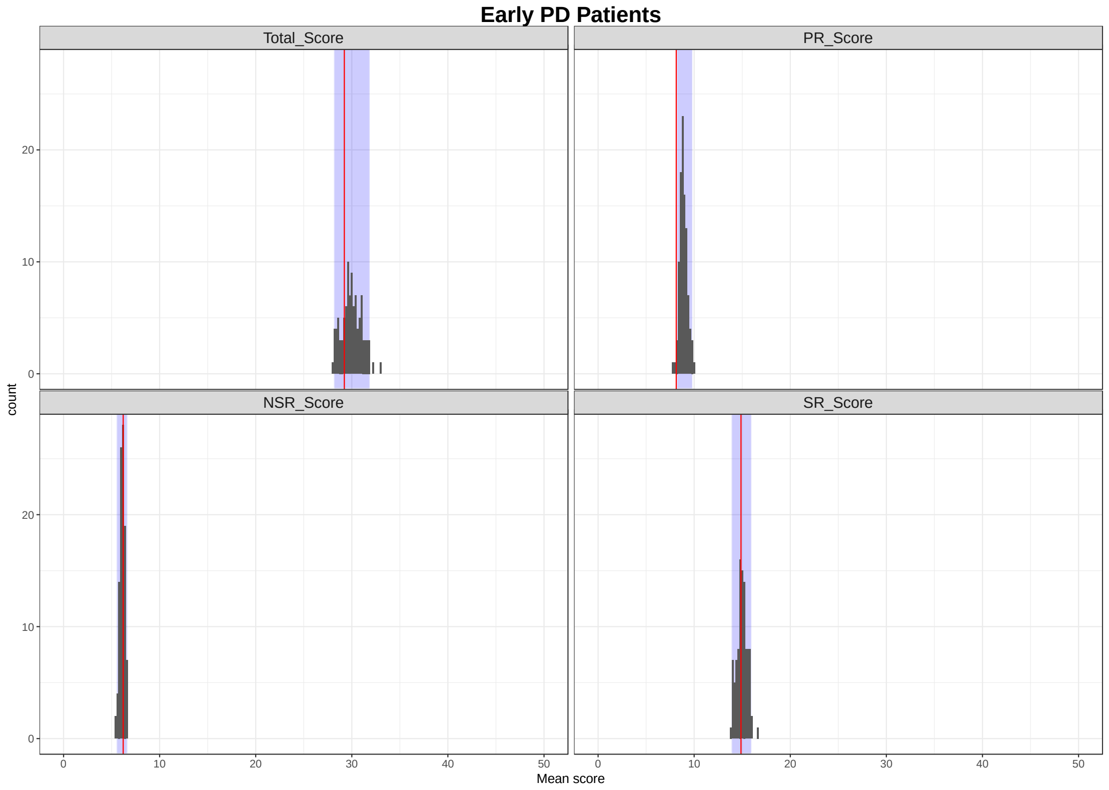

Figure 4Posterior predictive checkfor the total mean score in advanced PDsubjects, as well as stratified by latent variable (PR stands for patient reported, NSR for non-sided reported and SR for sided-reported)

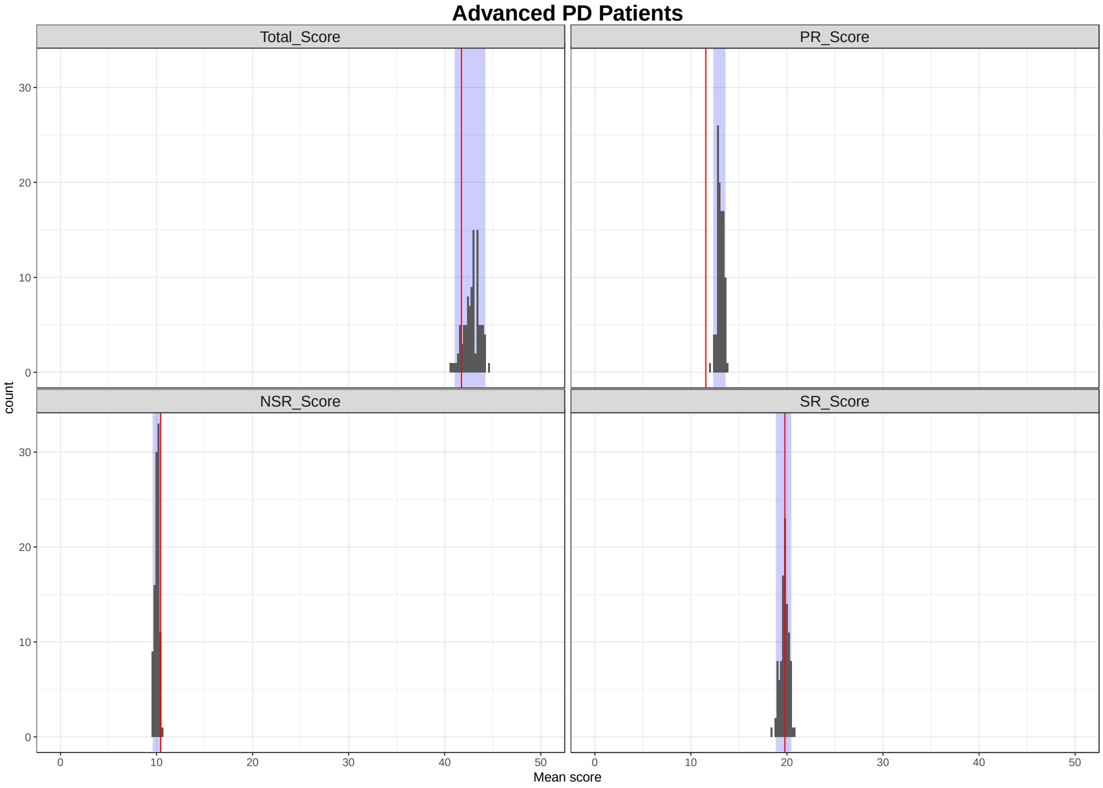

Figure 5Visual predictive checkfor the change from baseline for each of the three latent variables and the total score - Model Adaptation Using Longitudinal Placebo Data from Study 169.
PR_Score, NSR_Score and SR_Score represent the sum of the scores of items characterized by the latent variables PR, NSR, SR latent variables respectively (PR stands for patient reported, NSR for non-sided reported and SR for sided-reported). The blue lines represent the median (solid), 2.5th and 97.5th quantiles (dashed) of the observed data (points) with the respective 95% confidence intervals (shaded areas) based on the final longitudinal model.

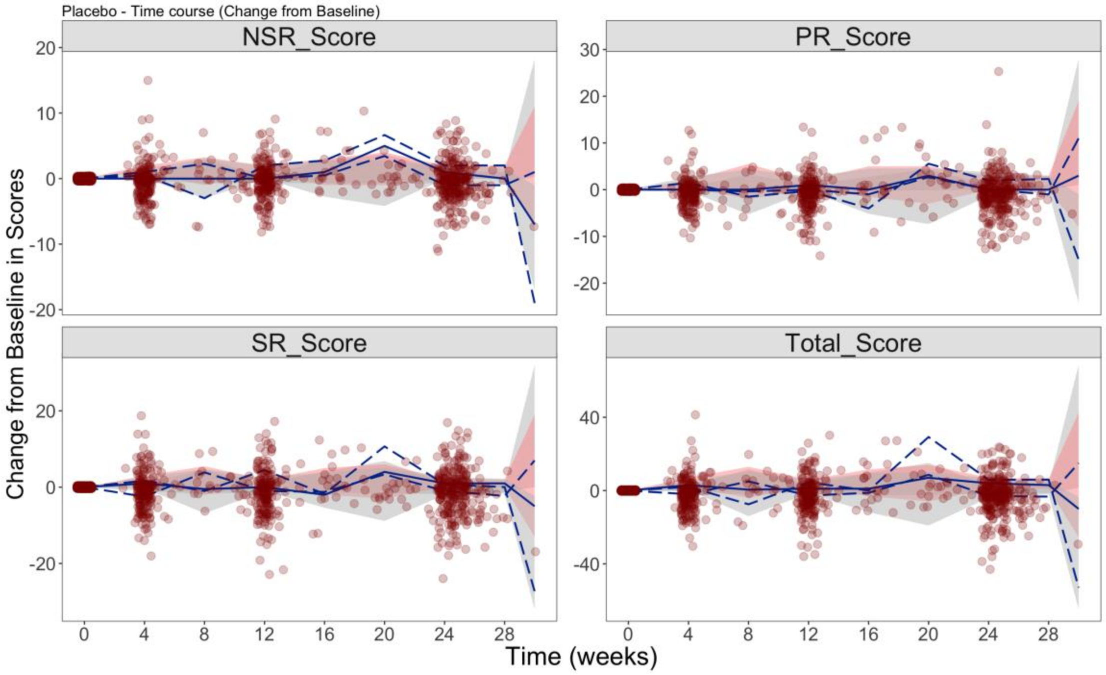

Supplement: Supplementary file 2 — (PDF 796 kb) [file 11095_2019_2668_MOESM2_ESM.pdf]
