# Supplementary material for: Item Response Model Adaptation for Analyzing Data from Different Versions of Parkinson’s Disease Rating Scales
Source: Pharm Res. 2019 Jul 17;36(9):135. doi: 10.1007/s11095-019-2668-6 (PMC6647468; doi:10.1007/s11095-019-2668-6)
Supplement: Supplementary file 3 — (PDF 565 kb) [file 11095_2019_2668_MOESM3_ESM.pdf]

Appendix – IIIModel and (excerpt) data files

```
;; 1. Based on:
;; 2. Description:
;; x1. Author: user
;*****************************************************************************
;      IRT in Parkinson's - combined model for UPDRS and MDS - UPDRS
;
; NOTE1:         If reading in UPDRS data, in $INPUT have ITEMU = ITEM
;         and If reading in MDSUPDRS data, in $INPUT have ITEMM = ITEM
;
; NOTE2:  Create another column in dataset itself:
;        ITEMM or ITEMU (respective to NOTE1) with 0 values
;*****************************************************************************
$SIZES      LTH=500 MAXIDS=50000  DIMNEW=7000
$PROBLEM    IRT in Parkinson's Disease (GSK data) - Combined model for
;          UPDRS and MDS-UPDRS
$INPUT    STID ; Clinical study identifier
          ID             ; Subject identifier
          ITEMU          ; Item #: Name of the efficacy item in UPDRS scale
          DV             ; Value for the efficacy item
          TIME           ; Time (in weeks) from the baseline visit -  accounting for item DO
          PRD  ; Study period prior to follow up period
          DOSE           ; Daily dose (mg/day)
          AGE  ; Age at randomization (years)
          WT             ; Weight at randomization (kg)
          HT             ; Height at randomization (cm)
          ETHNICITY      ; Race of subject
          SEX            ; Gender
       CMSE    ; Prior concomitant selegiline
       CMAM    ; Prior concomitant amantadine
       CMAC    ; Prior concomitant anti-cholinergics
       CMDP    ; Prior concomitant L-dopa.
       FORM    ; Formulation
       TSDW    ; Time since diagnosis of PD (in weeks)
       EXCL    ; Variable to exclude SUBJ 4850 who did not have demographic data
       ENDPTCAT        ; Endpoint category
       SEQ     ; Sequence for study 168
       TMT             ; Treatment for study 169
       DROPOUT         ; Subject dropped
       RDO     ; Reason for drop out
       VISITW  ; Time (in weeks) from the baseline visit - not accounting for item DO
       VISITD  ; Time (in days) from the baseline visit - not accounting for item DO
       TDOW    ; Time at which a subject dropped out (in weeks)
       ITEMM   ; Item #: Name of the efficacy item in MDS-UPDRS scale
       PBO             ; Flag for Placebo arm
$DATA      Beta5.csv IGNORE=@ IGNORE=(EXCL.EQ.1)
       IGNORE=(ENDPTCAT.NE.1) IGNORE=(ITEMU.EQ.32)
       IGNORE=(ITEMU.EQ.33) IGNORE=(ITEMU.EQ.34)
       IGNORE=(ITEMU.EQ.35) IGNORE=(ITEMU.EQ.36)
       IGNORE=(ITEMU.EQ.37) IGNORE=(ITEMU.EQ.38)
       IGNORE=(ITEMU.EQ.39) IGNORE=(ITEMU.EQ.40)
       IGNORE=(ITEMU.EQ.41) IGNORE=(ITEMU.EQ.42)
       IGNORE=(ITEMU.EQ.43) IGNORE=(ITEMU.EQ.44)
```

```
        IGNORE=(ITEMU.EQ.50) IGNORE=(TIME.GT.0)
        IGNORE=(DOSE.EQ.-99) IGNORE=(DV.EQ.-99)

$PRED

;------------------------------ Constant selection ------------------------------
; Constants to select the model type
GR4=1           ; Graded response 0-4 model

; Values of constants for reference
MODEL=0
ENDPT=0
ITEM=0
;-------------------------------- Item selection --------------------------------
IF(ITEMU.EQ.0)  ENDPT = 1     ; For MDS: If the dataset in $INPUT has ITEMM - MDSUPDRS data (and ITEMU=0)

IF(ITEMM.EQ.0)  ENDPT = 2     ; For UPDRS: If the dataset in $INPUT has ITEMU - UPDRS data

;*******************************************************************************
; Item re-ordered based on UPDRS
; - easy for handling the GSK data and potentially later on for plotting
;*******************************************************************************
; Item 1: Cognitive impairment in M (#1, 1.1) and Intellectual impairment in U (#1) respectively

IF(ITEMM.EQ.1.AND.ENDPT.EQ.1) ITEM = 1
IF(ITEMU.EQ.1.AND.ENDPT.EQ.2) ITEM = 1

; Mapping (U->M): 0->0, 1->2, 2->3, 3->4, 4->4
IF(ITEM.EQ.1) THEN
MODEL=GR4
DIS=THETA(1)          ;I1DISGR4
DIF1=THETA(2)         ;I1DIF1GR4
DIF2=THETA(3)         ;I1DIF2GR4
DIF3=THETA(4)         ;I1DIF3GR4
DIF4=THETA(5)         ;I1DIF4GR4
FR1=THETA(6)          ;I1: Fraction based on mapping of 1 (in U) -> 2 (in M)
FR4=THETA(7)          ;I1: Fraction based on mapping 3,4 (in U) -> 4 (in M)
ENDIF
;*******************************************************************************

; Item 2: Hallucinations and psychosis in M (#2, 1.2) and Thought disorder in U (#2) respectively
IF(ITEMM.EQ.2.AND.ENDPT.EQ.1) ITEM = 2
IF(ITEMU.EQ.2.AND.ENDPT.EQ.2) ITEM = 2

; Mapping (U->M): 0->0, 1->0, 2->1|2, 3->3, 4->4
IF(ITEM.EQ.2) THEN
MODEL=GR4
DIS=THETA(8)                ;I2DISGR4
DIF1=THETA(9)               ;I2DIF1GR4
DIF2=THETA(10)              ;I2DIF2GR4
DIF3=THETA(11)              ;I2DIF3GR4
DIF4=THETA(12)              ;I2DIF4GR4
FR0=THETA(13)               ;I2: Fraction based on mapping 0,1 (in U) -> 1 (in M)
ENDIF
;*******************************************************************************
```

```
;Item 3: Depressed mood in M (#3, 1.3) and Depression in U (#3) respectively
IF(ITEMM.EQ.3.AND.ENDPT.EQ.1) ITEM = 3
IF(ITEMU.EQ.3.AND.ENDPT.EQ.2) ITEM = 3

;Mapping (U->M): 0->0, 1->1, 2->2, 3->3, 4->4
IF(ITEM.EQ.3) THEN
MODEL=GR4
DIS=THETA(14)            ;I3DISGR4
DIF1=THETA(15)           ;I3DIF1GR4
DIF2=THETA(16)           ;I3DIF2GR4
DIF3=THETA(17)           ;I3DIF3GR4
DIF4=THETA(18)           ;I3DIF4GR4
ENDIF
;********************************************************************************

; Item 4: Apathy in M (#5, 1.5) and Motivation/Initiative in U (#4) respectively

IF(ITEMM.EQ.5.AND.ENDPT.EQ.1) ITEM = 4
IF(ITEMU.EQ.4.AND.ENDPT.EQ.2) ITEM = 4

;Mapping (U->M): 0->0, 1->1, 2->2, 3->3, 4->4
IF(ITEM.EQ.4) THEN
MODEL=GR4
DIS=THETA(19)            ;I4DISGR4
DIF1=THETA(20)           ;I4DIF1GR4
DIF2=THETA(21)           ;I4DIF2GR4
DIF3=THETA(22)           ;I4DIF3GR4
DIF4=THETA(23)           ;I4DIF4GR4
ENDIF
;********************************************************************************

; Item 5: Speech (ADL) in M (#14, 2.1) and Speech (ADL) in U (#5) respectively

IF(ITEMM.EQ.14.AND.ENDPT.EQ.1) ITEM = 5
IF(ITEMU.EQ.5.AND.ENDPT.EQ.2) ITEM = 5

;Mapping (U->M): 0->0, 1->1, 2->2, 3->3, 4->4
IF(ITEM.EQ.5) THEN
MODEL=GR4
DIS=THETA(24)            ;I5DISGR4
DIF1=THETA(25)           ;I5DIF1GR4
DIF2=THETA(26)           ;I5DIF2GR4
DIF3=THETA(27)           ;I5DIF3GR4
DIF4=THETA(28)           ;I5DIF4GR4
ENDIF
;******************************************************************************* **

; Item 6: Saliva and drooling in M (#15, 2.2) and Salivation in U (#6) respectively

IF(ITEMM.EQ.15.AND.ENDPT.EQ.1) ITEM = 6
IF(ITEMU.EQ.6.AND.ENDPT.EQ.2) ITEM = 6
```

```
; Mapping (U->M): 0->0, 1->2, 2->3, 3->3, 4->4
IF(ITEM.EQ.6) THEN
MODEL=GR4
DIS=THETA(29)                  ;I6DISGR4
DIF1=THETA(30)                 ;I6DIF1GR4
DIF2=THETA(31)                 ;I6DIF2GR4
DIF3=THETA(32)                 ;I6DIF3GR4
DIF4=THETA(33)                 ;I6DIF4GR4
FR1 = THETA(34)                ; FR1 split
FR3 = THETA(35)                ; FR3 split
ENDIF
;*****************************************************************************

; Item 7: Chewing and swallowing in M (#16, 2.3) and Swallowing in U (#7) respectively

IF(ITEMM.EQ.16.AND.ENDPT.EQ.1) ITEM = 7
IF(ITEMU.EQ.7.AND.ENDPT.EQ.2) ITEM = 7

;Mapping (U->M): 0->0, 1->3, 2->3, 3->2, 4->4
IF(ITEM.EQ.7) THEN
MODEL=GR4
DIS=THETA(36)                  ;I7DISGR4
DIF1=THETA(37)                 ;I7DIF1GR4
DIF2=THETA(38)                 ;I7DIF2GR4
DIF3=THETA(39)                 ;I7DIF3GR4
DIF4=THETA(40)                 ;I7DIF4GR4
FR1=THETA(41)                  ;Fraction based on mapping
FR3=THETA(42)                  ;Fraction based on mapping
ENDIF
;*****************************************************************************

; Item 8: Handwriting in M (#20, 2.7) and Handwriting in U (#8) respectively

IF(ITEMM.EQ.20.AND.ENDPT.EQ.1) ITEM = 8
IF(ITEMU.EQ.8.AND.ENDPT.EQ.2) ITEM = 8

;Mapping (U->M): 0->0, 1->1, 2->1, 3->2|3, 4->4
IF(ITEM.EQ.8) THEN
MODEL=GR4
DIS=THETA(43)                  ;I8DISGR4
DIF1=THETA(44)                 ;I8DIF1GR4
DIF2=THETA(45)                 ;I8DIF2GR4
DIF3=THETA(46)                 ;I8DIF3GR4
DIF4=THETA(47)                 ;I8DIF4GR4
FR1=THETA(48)                  ;Fraction based on mapping
ENDIF
;*****************************************************************************

; Item 9: Eating tasks in M (#17, 2.4) and Cutting food and handling utensils in U (#9) respectively

IF(ITEMM.EQ.17.AND.ENDPT.EQ.1) ITEM = 9
IF(ITEMU.EQ.9.AND.ENDPT.EQ.2) ITEM = 9
```

```
; Mapping (U->M): 0->0, 1->2, 2->3, 3->3, 4->4
IF(ITEM.EQ.9) THEN
MODEL=GR4
DIS=THETA(49)                ;I9DISGR4
DIF1=THETA(50)               ;I9DIF1GR4
DIF2=THETA(51)               ;I9DIF2GR4
DIF3=THETA(52)               ;I9DIF3GR4
DIF4=THETA(53)               ;I9DIF4GR4
ENDIF
;*******************************************************************************

; Item 10: Dressing in M (#18, 2.5) and Dressing in U (#10) respectively

IF(ITEMM.EQ.18.AND.ENDPT.EQ.1) ITEM = 10
IF(ITEMU.EQ.10.AND.ENDPT.EQ.2) ITEM = 10

; Mapping (U->M): 0->0, 1->2, 2->3, 3->3, 4->4
IF(ITEM.EQ.10) THEN
MODEL=GR4
DIS=THETA(54)                ;I10DISGR4
DIF1=THETA(55)               ;I10DIF1GR4
DIF2=THETA(56)               ;I10DIF2GR4
DIF3=THETA(57)               ;I10DIF3GR4
DIF4=THETA(58)               ;I10DIF4GR4
ENDIF
;*******************************************************************************

; Item 11: Hygiene in M (#19, 2.6) and Hygiene in U (#11) respectively

IF(ITEMM.EQ.19.AND.ENDPT.EQ.1) ITEM = 11
IF(ITEMU.EQ.11.AND.ENDPT.EQ.2) ITEM = 11

; Mapping (U->M): 0->0, 1->2, 2->3, 3->3, 4->4
IF(ITEM.EQ.11) THEN
MODEL=GR4
DIS=THETA(59)                ;I11DISGR4
DIF1=THETA(60)               ;I11DIF1GR4
DIF2=THETA(61)               ;I11DIF2GR4
DIF3=THETA(62)               ;I11DIF3GR4
DIF4=THETA(63)               ;I11DIF4GR4
ENDIF
;*******************************************************************************

; Item 12: Turning in bed in M (#22, 2.9) and Turning in bed and adjusting clothes in U (#12) respectively

IF(ITEMM.EQ.22.AND.ENDPT.EQ.1) ITEM = 12
IF(ITEMU.EQ.12.AND.ENDPT.EQ.2) ITEM = 12

;Mapping (U->M): 0->0, 1->1, 2->2|3, 3->3|4, 4->4
IF(ITEM.EQ.12) THEN
MODEL=GR4
DIS=THETA(64)                ;I12DISGR4
DIF1=THETA(65)               ;I12DIF1GR4
DIF2=THETA(66)               ;I12DIF2GR4
```

```
DIF3=THETA(67)                  ;I12DIF3GR4
DIF4=THETA(68)                  ;I12DIF4GR4
FR3=THETA(69)                   ;Fraction based on mapping
FR4=THETA(70)                   ;Fraction based on mapping
ENDIF
;*********************************************************************************

; Item 13: Falling in U (#13) - ONLY in U

IF(ITEMU.EQ.13.AND.ENDPT.EQ.2) ITEM = 13
IF(ITEM.EQ.13) THEN

MODEL=GR4
DIS=THETA(71)                   ;I13DISGR4
DIF1=THETA(72)                  ;I13DIF1GR4
DIF2=THETA(73)                  ;I13DIF2GR4
DIF3=THETA(74)                  ;I13DIF3GR4
DIF4=THETA(75)                  ;I13DIF4GR4
ENDIF
;*********************************************************************************

; Item 14: Freezing in M (#26, 2.13) and Freezing when walking in U (#14) - PARTIAL PARALLELISM
; MDS part coded as Item 45 (after all the U code)

IF(ITEMM.EQ.26.AND.ENDPT.EQ.1) ITEM = 14
IF(ITEMU.EQ.14.AND.ENDPT.EQ.2) ITEM = 14

;Mapping (U->M): 0->0, 1->1, 2->2|3|4, 3->2|3|4, 4->2|3|4 - IRRELEVANT - Cuz estimates as new item
IF(ITEM.EQ.14) THEN
MODEL=GR4
DIS=THETA(76)                   ;I14DISGR4
DIF1=THETA(77)                  ;I14DIF1GR4
DIF2=THETA(78)                  ;I14DIF2GR4
DIF3=THETA(79)                  ;I14DIF3GR4
DIF4=THETA(80)                  ;I14DIF4GR4
ENDIF
;*********************************************************************************

; Item 15: Walking and balance in M (#25, 2.12) and Walking in U (#15) respectively

IF(ITEMM.EQ.25.AND.ENDPT.EQ.1) ITEM = 15
IF(ITEMU.EQ.15.AND.ENDPT.EQ.2) ITEM = 15

;Mapping (U->M): 0->0, 1->1, 2->1|2, 3->3|4, 4->4

IF(ITEM.EQ.15) THEN
MODEL=GR4
DIS=THETA(81)                   ;I15DISGR4
DIF1=THETA(82)                  ;I15DIF1GR4
DIF2=THETA(83)                  ;I15DIF2GR4
DIF3=THETA(84)                  ;I15DIF3GR4
DIF4=THETA(85)                  ;I15DIF4GR4
FR1=THETA(86)                   ;Fraction based on mapping
```

```
FR4=THETA(87)                 ;Fraction based on mapping
ENDIF
;*******************************************************************************

; Item 16: Tremor (ADL) in M (#23, 2.10) and Tremor (ADL) in U (#16) respectively

IF(ITEMM.EQ.23.AND.ENDPT.EQ.1) ITEM = 16
IF(ITEMU.EQ.16.AND.ENDPT.EQ.2) ITEM = 16

; Mapping (U->M): 0->0, 1->1, 2->2, 3->3, 4->4
IF(ITEM.EQ.16) THEN
MODEL=GR4
DIS=THETA(88)                 ;I16DISGR4
DIF1=THETA(89)                ;I16DIF1GR4
DIF2=THETA(90)                ;I16DIF2GR4
DIF3=THETA(91)                ;I16DIF3GR4
DIF4=THETA(92)                ;I16DIF4GR4
ENDIF
;*******************************************************************************

; Item 17: Pain and other sensations in M (#9, 1.9) and Sensory complaints related to PD in U (#17) respectively

IF(ITEMM.EQ.9.AND.ENDPT.EQ.1) ITEM = 17
IF(ITEMU.EQ.17.AND.ENDPT.EQ.2) ITEM = 17

; Mapping (U->M): 0->0, 1->1, 2->2, 3->3, 4->4

IF(ITEM.EQ.17) THEN
MODEL=GR4
DIS=THETA(93)                 ;I17DISGR4
DIF1=THETA(94)                ;I17DIF1GR4
DIF2=THETA(95)                ;I17DIF2GR4
DIF3=THETA(96)                ;I17DIF3GR4
DIF4=THETA(97)                ;I17DIF4GR4
ENDIF
;*******************************************************************************

; Item 18: Speech (motor examination) in M (#27, 3.1) and Speech (motor examination) in U (#18) respectively

IF(ITEMM.EQ.27.AND.ENDPT.EQ.1) ITEM = 18
IF(ITEMU.EQ.18.AND.ENDPT.EQ.2) ITEM = 18

;Mapping (U->M): 0->0, 1->1, 2->2, 3->3|4, 4->4

IF(ITEM.EQ.18) THEN
MODEL=GR4
DIS=THETA(98)                 ;I18DISGR4
DIF1=THETA(99)                ;I18DIF1GR4
DIF2=THETA(100)               ;I18DIF2GR4
DIF3=THETA(101)               ;I18DIF3GR4
DIF4=THETA(102)               ;I18DIF4GR4
FR4=THETA(103)                ;Fraction based on mapping
ENDIF
;*******************************************************************************
```

```
; Item 19: Facial expression in M (#28, 3.2) and Facial expression in U (#19) respectively

IF(ITEMM.EQ.28.AND.ENDPT.EQ.1) ITEM = 19
IF(ITEMU.EQ.19.AND.ENDPT.EQ.2) ITEM = 19

; Mapping (U->M): 0->0, 1->1, 2->2, 3->3, 4->4

IF(ITEM.EQ.19) THEN
MODEL=GR4
DIS=THETA(104)              ;I19DISGR4
DIF1=THETA(105)             ;I19DIF1GR4
DIF2=THETA(106)             ;I19DIF2GR4
DIF3=THETA(107)             ;I19DIF3GR4
DIF4=THETA(108)             ;I19DIF4GR4
ENDIF
;*******************************************************************************

; Item 200: Rest tremor amplitude - Face lips, chin in M (#58, 3.17e)
; and Tremor at rest - Face lips, chin in U (#200) - NO PARALLELISM
; MDS part coded as Item 46 (after all the U code)

IF(ITEMU.EQ.200.AND.ENDPT.EQ.2) ITEM = 200
IF(ITEM.EQ.200) THEN

MODEL=GR4
DIS=THETA(109)              ;I200DISGR4
DIF1=THETA(110)             ;I200DIF1GR4
DIF2=THETA(111)             ;I200DIF2GR4
DIF3=THETA(112)             ;I200DIF3GR4
DIF4=THETA(113)             ;I200DIF4GR4
ENDIF
;*******************************************************************************

; Item 201: Rest tremor amplitude - Left UE in M (#55, 3.17b)
; and Tremor at rest - Left hand in U (#201) - NO PARALLELISM
; MDS part coded as Item 47 (after all the U code)

IF(ITEMU.EQ.201.AND.ENDPT.EQ.2) ITEM = 201
IF(ITEM.EQ.201) THEN

MODEL=GR4
DIS=THETA(114)              ;I201DISGR4
DIF1=THETA(115)             ;I201DIF1GR4
DIF2=THETA(116)             ;I201DIF2GR4
DIF3=THETA(117)             ;I201DIF3GR4
DIF4=THETA(118)             ;I201DIF4GR4
ENDIF
;*******************************************************************************

; Item 202: Rest tremor amplitude - Right UE in M (#54, 3.17a)
; and Tremor at rest - Right hand in U (#202) - NO PARALLELISM
; MDS part coded as Item 48 (after all the U code)
```

```
IF(ITEMU.EQ.202.AND.ENDPT.EQ.2) ITEM = 202
IF(ITEM.EQ.202) THEN

MODEL=GR4
DIS=THETA(119)                  ;I202DISGR4
DIF1=THETA(120)                 ;I202DIF1GR4
DIF2=THETA(121)                 ;I202DIF2GR4
DIF3=THETA(122)                 ;I202DIF3GR4
DIF4=THETA(123)                 ;I202DIF4GR4
ENDIF
;******************************************************************************

; Item 203: Rest tremor amplitude - Left LE in M (#57, 3.17d)
; and Tremor at rest - Left foot in U (#203) - NO PARALLELISM
; MDS part coded as Item 49 (after all the U code)

IF(ITEMU.EQ.203.AND.ENDPT.EQ.2) ITEM = 203
IF(ITEM.EQ.203) THEN

MODEL=GR4
DIS=THETA(124)                  ;I203DISGR4
DIF1=THETA(125)                 ;I203DIF1GR4
DIF2=THETA(126)                 ;I203DIF2GR4
DIF3=THETA(127)                 ;I203DIF3GR4
DIF4=THETA(128)                 ;I203DIF4GR4
ENDIF
;******************************************************************************

; Item 204: Rest tremor amplitude - Right LE in M (#56, 3.17c)
; and Tremor at rest - Right foot in U (#204) - NO PARALLELISM
; MDS part coded as Item 50 (after all the U code)

IF(ITEMU.EQ.204.AND.ENDPT.EQ.2) ITEM = 204
IF(ITEM.EQ.204) THEN

MODEL=GR4
DIS=THETA(129)                  ;I204DISGR4
DIF1=THETA(130)                 ;I204DIF1GR4
DIF2=THETA(131)                 ;I204DIF2GR4
DIF3=THETA(132)                 ;I204DIF3GR4
DIF4=THETA(133)                 ;I204DIF4GR4
ENDIF
;******************************************************************************

; Item 205: Action/Postural tremor - Left hand in U (#205) - NO PARALLELISM
; MDS part for Postural (51, 3.15b) and Kinetic tremor (53, 3.16b) for left hand
;  -- each coded as Items 51 and 52 (after U part) respectively

IF(ITEMU.EQ.205.AND.ENDPT.EQ.2) ITEM = 205
IF(ITEM.EQ.205) THEN

MODEL=GR4
DIS=THETA(134)                  ;I205DISGR4
DIF1=THETA(135)                 ;I205DIF1GR4
```

```
DIF2=THETA(136)                 ;I205DIF2GR4
DIF3=THETA(137)                 ;I205DIF3GR4
DIF4=THETA(138)                 ;I205DIF4GR4
ENDIF
;***************************************************************************************

; Item 206: Action/Postural tremor  - Right hand in U (#206) - NO PARALLELISM
; MDS part for Postural (50, 3.15a) and Kinetic tremor (52, 3.16a) for right hand
;  -- each coded as Items 53 and 54 (after U part) respectively

IF(ITEMU.EQ.206.AND.ENDPT.EQ.2) ITEM = 206
IF(ITEM.EQ.206) THEN

MODEL=GR4
DIS=THETA(139)                  ;I206DISGR4
DIF1=THETA(140)                 ;I206DIF1GR4
DIF2=THETA(141)                 ;I206DIF2GR4
DIF3=THETA(142)                 ;I206DIF3GR4
DIF4=THETA(143)                 ;I206DIF4GR4
ENDIF
;***************************************************************************************

; Item 207: Rigidity - Neck in M (#29, 3.3a) and Rigidity - Neck in U (#207) respectively

IF(ITEMM.EQ.29.AND.ENDPT.EQ.1) ITEM = 207
IF(ITEMU.EQ.207.AND.ENDPT.EQ.2) ITEM = 207

;Mapping (U->M): 0->0, 1->1, 2->2, 3->2, 4->3|4

IF(ITEM.EQ.207) THEN
MODEL=GR4
DIS=THETA(144)                  ;I207DISGR4
DIF1=THETA(145)                 ;I207DIF1GR4
DIF2=THETA(146)                 ;I207DIF2GR4
DIF3=THETA(147)                 ;I207DIF3GR4
DIF4=THETA(148)                 ;I207DIF4GR4
FR2=THETA(149)                  ;Fraction based on mapping
FR4=THETA(150)                  ;Fraction based on mapping
ENDIF
;***************************************************************************************

; Item 208: Rigidity - Left UE in M (#31, 3.3c) and Rigidity - Left UE in U (#208) respectively

IF(ITEMM.EQ.31.AND.ENDPT.EQ.1) ITEM = 208
IF(ITEMU.EQ.208.AND.ENDPT.EQ.2) ITEM = 208

;Mapping (U->M): 0->0, 1->1, 2->2, 3->2, 4->3|4

IF(ITEM.EQ.208) THEN
MODEL=GR4
DIS=THETA(151)                  ;I208DISGR4
DIF1=THETA(152)                 ;I208DIF1GR4
DIF2=THETA(153)                 ;I208DIF2GR4
DIF3=THETA(154)                 ;I208DIF3GR4
DIF4=THETA(155)                 ;I208DIF4GR4
```

```
FR2=THETA(156)                  ;Fraction based on mapping
FR4=THETA(157)                  ;Fraction based on mapping
ENDIF
;*******************************************************************************

; Item 209: Rigidity - Right UE in M (#30, 3.3b) and Rigidity - Right UE  in U (#209) respectively

IF(ITEMM.EQ.30.AND.ENDPT.EQ.1) ITEM = 209
IF(ITEMU.EQ.209.AND.ENDPT.EQ.2) ITEM = 209

;Mapping (U->M): 0->0, 1->1, 2->2, 3->2, 4->3|4

IF(ITEM.EQ.209) THEN
MODEL=GR4
DIS=THETA(158)                  ;I209DISGR4
DIF1=THETA(159)                 ;I209DIF1GR4
DIF2=THETA(160)                 ;I209DIF2GR4
DIF3=THETA(161)                 ;I209DIF3GR4
DIF4=THETA(162)                 ;I209DIF4GR4
FR2=THETA(163)                  ;Fraction based on mapping
FR4=THETA(164)                  ;Fraction based on mapping
ENDIF
;*******************************************************************************

; Item 210: Rigidity - Left LE in M (#33, 3.3e) and Rigidity - Left LE in U (#210) respectively

IF(ITEMM.EQ.33.AND.ENDPT.EQ.1) ITEM = 210
IF(ITEMU.EQ.210.AND.ENDPT.EQ.2) ITEM = 210

;Mapping (U->M): 0->0, 1->1, 2->2, 3->2, 4->3|4

IF(ITEM.EQ.210) THEN
MODEL=GR4
DIS=THETA(165)                  ;I210DISGR4
DIF1=THETA(166)                 ;I210DIF1GR4
DIF2=THETA(167)                 ;I210DIF2GR4
DIF3=THETA(168)                 ;I210DIF3GR4
DIF4=THETA(169)                 ;I210DIF4GR4
FR2=THETA(170)                  ;Fraction based on mapping
FR4=THETA(171)                  ;Fraction based on mapping
ENDIF
;*******************************************************************************

; Item 211 : Rigidity - Right LE in M (#32, 3.3d) and  Rigidity - Right LE in U (#211) respectively

IF(ITEMM.EQ.32.AND.ENDPT.EQ.1) ITEM = 211
IF(ITEMU.EQ.211.AND.ENDPT.EQ.2) ITEM = 211

;Mapping (U->M): 0->0, 1->1, 2->2, 3->2, 4->3|4

IF(ITEM.EQ.211) THEN
MODEL=GR4
DIS=THETA(172)                  ;I211DISGR4
DIF1=THETA(173)                 ;I211DIF1GR4
DIF2=THETA(174)                 ;I211DIF2GR4
```

```
DIF3=THETA(175)                 ;I211DIF3GR4
DIF4=THETA(176)                 ;I211DIF4GR4
FR2=THETA(177)                  ;Fraction based on mapping
FR4=THETA(178)                  ;Fraction based on mapping
ENDIF
;*****************************************************************************

; Item 212: Finger taps left in M (#35, 3.4b) and Finger taps left in U (#212) respectively

IF(ITEMM.EQ.35.AND.ENDPT.EQ.1) ITEM = 212
IF(ITEMU.EQ.212.AND.ENDPT.EQ.2) ITEM = 212

;Mapping (U->M): 0->0, 1->1|2, 2->2|3, 3->3, 4->4

IF(ITEM.EQ.212) THEN
MODEL=GR4
DIS=THETA(179)                  ;I212DISGR4
DIF1=THETA(180)                 ;I212DIF1GR4
DIF2=THETA(181)                 ;I212DIF2GR4
DIF3=THETA(182)                 ;I212DIF3GR4
DIF4=THETA(183)                 ;I212DIF4GR4
FR2=THETA(184)                  ;Fraction based on mapping
FR3=THETA(185)                  ;Fraction based on mapping
ENDIF
;*****************************************************************************

; Item 213: Finger taps right in M (#34, 3.4a) and Finger taps left in U (#213) respectively

IF(ITEMM.EQ.34.AND.ENDPT.EQ.1) ITEM = 213
IF(ITEMU.EQ.213.AND.ENDPT.EQ.2) ITEM = 213

;Mapping (U->M): 0->0, 1->1|2, 2->2|3, 3->3, 4->4

IF(ITEM.EQ.213) THEN
MODEL=GR4
DIS=THETA(186)                  ;I213DISGR4
DIF1=THETA(187)                 ;I213DIF1GR4
DIF2=THETA(188)                 ;I213DIF2GR4
DIF3=THETA(189)                 ;I213DIF3GR4
DIF4=THETA(190)                 ;I213DIF4GR4
FR2=THETA(191)                  ;Fraction based on mapping
FR3=THETA(192)                  ;Fraction based on mapping
ENDIF
;*****************************************************************************

; Item 214: Hand movement - left in M (#37, 3.5b) and Hand movement in U (#214) respectively

IF(ITEMM.EQ.37.AND.ENDPT.EQ.1) ITEM = 214
IF(ITEMU.EQ.214.AND.ENDPT.EQ.2) ITEM = 214

;Mapping (U->M): 0->0, 1->1|2, 2->2|3, 3->3, 4->4

IF(ITEM.EQ.214) THEN
MODEL=GR4
DIS=THETA(193)                  ;I214DISGR4
```

```
DIF1=THETA(194)          ;I214DIF1GR4
DIF2=THETA(195)          ;I214DIF2GR4
DIF3=THETA(196)          ;I214DIF3GR4
DIF4=THETA(197)          ;I214DIF4GR4
FR2=THETA(198)           ;Fraction based on mapping
FR3=THETA(199)           ;Fraction based on mapping
ENDIF
;*********************************************************************************

; Item 215: Hand movement - right in M (#36, 3.5a) and Hand movement in U (#215) respectively

IF(ITEMM.EQ.36.AND.ENDPT.EQ.1) ITEM = 215
IF(ITEMU.EQ.215.AND.ENDPT.EQ.2) ITEM = 215

;Mapping (U->M): 0->0, 1->1|2, 2->2|3, 3->3, 4->4

IF(ITEM.EQ.215) THEN
MODEL=GR4
DIS=THETA(200)           ;I215DISGR4
DIF1=THETA(201)          ;I215DIF1GR4
DIF2=THETA(202)          ;I215DIF2GR4
DIF3=THETA(203)          ;I215DIF3GR4
DIF4=THETA(204)          ;I215DIF4GR4
FR2=THETA(205)           ;Fraction based on mapping
FR3=THETA(206)           ;Fraction based on mapping
ENDIF
;*********************************************************************************

; Item 216: Hand pronation and supination - left in M (#39, 3.6b) and Hand pronation and supination - left in U
;(#216) respectively

IF(ITEMM.EQ.39.AND.ENDPT.EQ.1) ITEM = 216
IF(ITEMU.EQ.216.AND.ENDPT.EQ.2) ITEM = 216

;Mapping (U->M): 0->0, 1->1|2, 2->2|3, 3->3, 4->4

IF(ITEM.EQ.216) THEN
MODEL=GR4
DIS=THETA(207)           ;I216DISGR4
DIF1=THETA(208)          ;I216DIF1GR4
DIF2=THETA(209)          ;I216DIF2GR4
DIF3=THETA(210)          ;I216DIF3GR4
DIF4=THETA(211)          ;I216DIF4GR4
FR2=THETA(212)           ;Fraction based on mapping
FR3=THETA(213)           ;Fraction based on mapping
ENDIF
;*********************************************************************************

; Item 217: Hand pronation and supination - right in M (#38, 3.6a) and Hand pronation and supination - left in U
(#217) respectively

IF(ITEMM.EQ.38.AND.ENDPT.EQ.1) ITEM = 217
IF(ITEMU.EQ.217.AND.ENDPT.EQ.2) ITEM = 217
```

```
;Mapping (U->M): 0->0, 1->1|2, 2->2|3, 3->3, 4->4

IF(ITEM.EQ.217) THEN
MODEL=GR4
DIS=THETA(214)                  ;I217DISGR4
DIF1=THETA(215)                 ;I217DIF1GR4
DIF2=THETA(216)                 ;I217DIF2GR4
DIF3=THETA(217)                 ;I217DIF3GR4
DIF4=THETA(218)                 ;I217DIF4GR4
FR2=THETA(219)                  ;Fraction based on mapping
FR3=THETA(220)                  ;Fraction based on mapping
ENDIF
;*******************************************************************************

; Item 218: Leg agility - left in M (#43, 3.8b) and Leg agility - left in U (#218) respectively

IF(ITEMM.EQ.43.AND.ENDPT.EQ.1) ITEM = 218
IF(ITEMU.EQ.218.AND.ENDPT.EQ.2) ITEM = 218

;Mapping (U->M): 0->0, 1->1|2, 2->2|3, 3->3, 4->4

IF(ITEM.EQ.218) THEN
MODEL=GR4
DIS=THETA(221)                  ;I218DISGR4
DIF1=THETA(222)                 ;I218DIF1GR4
DIF2=THETA(223)                 ;I218DIF2GR4
DIF3=THETA(224)                 ;I218DIF3GR4
DIF4=THETA(225)                 ;I218DIF4GR4
FR2=THETA(226)                  ;Fraction based on mapping
FR3=THETA(227)                  ;Fraction based on mapping
ENDIF
;*******************************************************************************

; Item 219: Leg agility - right in M (#42, 3.8a) and Leg agility - right in U (#219) respectively

IF(ITEMM.EQ.42.AND.ENDPT.EQ.1) ITEM = 219
IF(ITEMU.EQ.219.AND.ENDPT.EQ.2) ITEM = 219

;Mapping (U->M): 0->0, 1->1|2, 2->2|3, 3->3, 4->4

IF(ITEM.EQ.219) THEN
MODEL=GR4
DIS=THETA(228)                  ;I219DISGR4
DIF1=THETA(229)                 ;I219DIF1GR4
DIF2=THETA(230)                 ;I219DIF2GR4
DIF3=THETA(231)                 ;I219DIF3GR4
DIF4=THETA(232)                 ;I219DIF4GR4
FR2=THETA(233)                  ;Fraction based on mapping
FR3=THETA(234)                  ;Fraction based on mapping
ENDIF
;*******************************************************************************

; Item 27: Arising from chair in M (#44, 3.9) and Arising from chair in U (#27) respectively
```

```
IF(ITEMM.EQ.44.AND.ENDPT.EQ.1) ITEM = 27
IF(ITEMU.EQ.27.AND.ENDPT.EQ.2) ITEM = 27

;Mapping (U->M): 0->0, 1->1, 2->2, 3->3, 4->4

IF(ITEM.EQ.27) THEN
MODEL=GR4
DIS=THETA(235)                  ;I27DISGR4
DIF1=THETA(236)                 ;I27DIF1GR4
DIF2=THETA(237)                 ;I27DIF2GR4
DIF3=THETA(238)                 ;I27DIF3GR4
DIF4=THETA(239)                 ;I27DIF4GR4
ENDIF
;*****************************************************************************

; Item 28: Posture in M (#48, 3.13) and Posture in U (#28) respectively

IF(ITEMM.EQ.48.AND.ENDPT.EQ.1) ITEM = 28
IF(ITEMU.EQ.28.AND.ENDPT.EQ.2) ITEM = 28

;Mapping (U->M): 0->0, 1->1, 2->2|3, 3->4, 4->4

IF(ITEM.EQ.28) THEN
MODEL=GR4
DIS=THETA(240)                  ;I28DISGR4
DIF1=THETA(241)                 ;I28DIF1GR4
DIF2=THETA(242)                 ;I28DIF2GR4
DIF3=THETA(243)                 ;I28DIF3GR4
DIF4=THETA(244)                 ;I28DIF4GR4
FR4=THETA(245)                  ;Fraction based on mapping
ENDIF
;*****************************************************************************

; Item 29: Gait in M (#45, 3.10) and Gait in U (#29) respectively

IF(ITEMM.EQ.45.AND.ENDPT.EQ.1) ITEM = 29
IF(ITEMU.EQ.29.AND.ENDPT.EQ.2) ITEM = 29

;Mapping (U->M): 0->0, 1->1, 2->2, 3->3|4, 4->4

IF(ITEM.EQ.29) THEN
MODEL=GR4
DIS=THETA(246)                  ;I29DISGR4
DIF1=THETA(247)                 ;I29DIF1GR4
DIF2=THETA(248)                 ;I29DIF2GR4
DIF3=THETA(249)                 ;I29DIF3GR4
DIF4=THETA(250)                 ;I29DIF4GR4
FR4=THETA(251)                  ;Fraction based on mapping
ENDIF
;*****************************************************************************

; Item 30: Postural stability in M (#47, 3.12) and Postural stability in U (#30) respectively
IF(ITEMM.EQ.47.AND.ENDPT.EQ.1) ITEM = 30
IF(ITEMU.EQ.30.AND.ENDPT.EQ.2) ITEM = 30
```

```
;Mapping (U->M): 0->0, 1->1|2, 2->3, 3->4, 4->4

IF(ITEM.EQ.30) THEN
MODEL=GR4
DIS=THETA(252)            ;I30DISGR4
DIF1=THETA(253)           ;I30DIF1GR4
DIF2=THETA(254)           ;I30DIF2GR4
DIF3=THETA(255)           ;I30DIF3GR4
DIF4=THETA(256)           ;I30DIF4GR4
FR4=THETA(257)            ;Fraction based on mapping
ENDIF
;******************************************************************************

; Item 31: Global spontaneity of movement in M (#49, 3.14) and Body bradykinesia and hypokinesia in U (#31)
respectively

IF(ITEMM.EQ.49.AND.ENDPT.EQ.1) ITEM = 31
IF(ITEMU.EQ.31.AND.ENDPT.EQ.2) ITEM = 31

;Mapping (U->M): 0->0, 1->1, 2->2, 3->3, 4->4

IF(ITEM.EQ.31) THEN

MODEL=GR4
DIS=THETA(258)            ;I31DISGR4
DIF1=THETA(259)           ;I31DIF1GR4
DIF2=THETA(260)           ;I31DIF2GR4
DIF3=THETA(261)           ;I31DIF3GR4
DIF4=THETA(262)           ;I31DIF4GR4
ENDIF
;******************************************************************************

;-----------------------------Hidden variable model------------------------------

;---------------------- Model implementation of PSI for different items -----------------

;******************************************************************************
;--------------------For Patient reported items---------------

PATITEMS=0

; PR Latent Variable: Items 1-17
IF(ITEM.LE.17) PATITEMS = 1

; Mean of PR latent variable [THETA(263)] and variance dependent on the study population
IF(PATITEMS.EQ.1.AND.STID.EQ.168) PSI=THETA(263) + ETA(1)

; PR: Shift parameter S2 [THETA(268)] for Advanced PD patients relative to Early PD patients (Reference Population)
IF(PATITEMS.EQ.1.AND.STID.EQ.169) PSI=THETA(263) + ETA(4) + THETA(268)

;******************************************************************************
```

```
;*******************************************************************************
;--------------------For Non-Dextrous reported items---------------

NDEXITEMS = 0

; NSR Latent Variable: Items as listed
IF(ITEM.EQ.18.OR.ITEM.EQ.19.OR.ITEM.EQ.200.OR.ITEM.EQ.207.OR.ITEM.EQ.27.OR.ITEM.EQ.28.OR.ITEM.EQ.29.OR
.ITEM.EQ.30.OR.ITEM.EQ.31) NDEXITEMS = 1

; Mean of NSR latent variable [THETA(264)] and variance dependent on the study population
IF(NDEXITEMS.EQ.1.AND.STID.EQ.168) PSI = THETA(264) + ETA(2)


; NSR: Shift parameter S3 [THETA(269)] for Advanced PD patients relative to Early PD patients (Reference
Population)
IF(NDEXITEMS.EQ.1.AND.STID.EQ.169) PSI = THETA(264) + ETA(5) + THETA(269)

;*******************************************************************************

;--------------------For Dextrous reported items---------------

LEFTBAD = 0               ; Classification based on mixture

IF(MIXNUM.EQ.2) LEFTBAD = 1               ; Based on worst side

LEFTITEMS = -1

; Defining items evaluating the right side
IF(ITEM.EQ.202.OR.ITEM.EQ.204.OR.ITEM.EQ.206.OR.ITEM.EQ.209.OR.ITEM.EQ.211.OR.ITEM.EQ.213.OR.ITEM.EQ.2
15.OR.ITEM.EQ.217.OR.ITEM.EQ.219) LEFTITEMS = 0 ; Right items


; Defining items evaluating the left side
IF(ITEM.EQ.201.OR.ITEM.EQ.203.OR.ITEM.EQ.205.OR.ITEM.EQ.208.OR.ITEM.EQ.210.OR.ITEM.EQ.212.OR.ITEM.EQ.2
14.OR.ITEM.EQ.216.OR.ITEM.EQ.218) LEFTITEMS = 1 ; Left items

; Mean of SR latent variable [THETA(265)] and variance dependent on the study population
;-----------------------------------------------------------------------------------------------------------
;Same set combination of evaluated and affected side
;Right side is more disabled side and items are evaluated on right side

IF(LEFTBAD.EQ.0.AND.LEFTITEMS.EQ.0.AND.STID.EQ.168) PSI = THETA(265) + ETA(3)

; SR: Same set combination of disabled and evaluated: Right
; Shift parameter S4 [THETA(270)] for Advanced PD patients relative to Early PD patients (Reference Population)
IF(LEFTBAD.EQ.0.AND.LEFTITEMS.EQ.0.AND.STID.EQ.169) PSI = THETA(265) + ETA(6) + THETA(270)


;Left side is more disabled side and items are evaluated on Left side
IF(LEFTBAD.EQ.1.AND.LEFTITEMS.EQ.1.AND.STID.EQ.168) PSI = THETA(265) + ETA(3)

; SR: Same set combination of disabled and evaluated: Left
; Shift parameter S4 [THETA(270)] for Advanced PD patients relative to Early PD patients (Reference Population)
IF(LEFTBAD.EQ.1.AND.LEFTITEMS.EQ.1.AND.STID.EQ.169) PSI = THETA(265) + ETA(6) + THETA(270)
```

```
;--------------------------------------------------------------------------------------------------------------------------

;Different set combination of evaluated and affected side:
;Compared to same set combination described above: S1 [THETA(266)] and associated variance [ETA(7)]
;Notes about S1:
;Constrained to be negative: reflecting lower disability for being different set combination
;Right side is more disabled side and items are evaluated on left side
IF(LEFTBAD.EQ.0.AND.LEFTITEMS.EQ.1.AND.STID.EQ.168) PSI = THETA(265) + ETA(3) - THETA(266)*EXP(ETA(7))


; SR: Different set combination of disabled and evaluated side
; Shift parameter S5 [THETA(271)] for Advanced PD patients relative to Early PD patients (Reference Population)
IF(LEFTBAD.EQ.0.AND.LEFTITEMS.EQ.1.AND.STID.EQ.169) PSI = THETA(265) + ETA(6) - THETA(266)*EXP(ETA(7)) +
THETA(271)


;Left side is more disabled side and items are evaluated on right side
IF(LEFTBAD.EQ.1.AND.LEFTITEMS.EQ.0.AND.STID.EQ.168) PSI = THETA(265) + ETA(3) - THETA(266)*EXP(ETA(7))


; SR: Different set combination of disabled and evaluated side
; Shift parameter S5 [THETA(271)] for Advanced PD patients relative to Early PD patients (Reference Population)
IF(LEFTBAD.EQ.1.AND.LEFTITEMS.EQ.0.AND.STID.EQ.169) PSI = THETA(265) + ETA(6) - THETA(266)*EXP(ETA(7)) +
THETA(271)


;----------------------Graded response model implementation GR 0 - 4 ---------------------
IF(MODEL.EQ.GR4) THEN
DIFG1=DIF1
DIFG2=DIFG1+DIF2
DIFG3=DIFG2+DIF3
DIFG4=DIFG3+DIF4

PGE1=EXP(DIS*(PSI-DIFG1))/(1+EXP(DIS*(PSI-DIFG1)))
PGE2=EXP(DIS*(PSI-DIFG2))/(1+EXP(DIS*(PSI-DIFG2)))
PGE3=EXP(DIS*(PSI-DIFG3))/(1+EXP(DIS*(PSI-DIFG3)))
PGE4=EXP(DIS*(PSI-DIFG4))/(1+EXP(DIS*(PSI-DIFG4)))

P0=1-PGE1
P1=PGE1-PGE2
P2=PGE2-PGE3
P3=PGE3-PGE4
P4=PGE4
ENDIF
;--------------------------------------------------------------------------------------------


; For UPDRS - ITEM 1: ; Cognitive impairment in M (#1, 1.1) and Intellectual impairment in U (#1)
IF(MODEL.EQ.GR4.AND.ENDPT.EQ.2.AND.ITEM.EQ.1.AND.DV.EQ.0) P=P0 + P1*FR1        ; mapped 0->0 (U->M)
IF(MODEL.EQ.GR4.AND.ENDPT.EQ.2.AND.ITEM.EQ.1.AND.DV.EQ.1) P=P2 + P1*(1-FR1)    ; mapped 1->2
IF(MODEL.EQ.GR4.AND.ENDPT.EQ.2.AND.ITEM.EQ.1.AND.DV.EQ.2) P=P3                 ; mapped 2->3
IF(MODEL.EQ.GR4.AND.ENDPT.EQ.2.AND.ITEM.EQ.1.AND.DV.EQ.3) P=P4*FR4             ; mapped 3->4
IF(MODEL.EQ.GR4.AND.ENDPT.EQ.2.AND.ITEM.EQ.1.AND.DV.EQ.4) P=P4*(1 - FR4)       ; mapped 4->4
IF(MODEL.EQ.GR4.AND.ENDPT.EQ.2.AND.ITEM.EQ.1) UP0 = P0 + P1*FR1                ; mapped 0->0 (U->M)
```

```
IF(MODEL.EQ.GR4.AND.ENDPT.EQ.2.AND.ITEM.EQ.1) UP1 = P2 + P1*(1-FR1)               ; mapped 1->2
IF(MODEL.EQ.GR4.AND.ENDPT.EQ.2.AND.ITEM.EQ.1) UP2 = P3                            ; mapped 2->3
IF(MODEL.EQ.GR4.AND.ENDPT.EQ.2.AND.ITEM.EQ.1) UP3 = P4*FR4                        ; mapped 3->4
IF(MODEL.EQ.GR4.AND.ENDPT.EQ.2.AND.ITEM.EQ.1) UP4 = P4*(1 - FR4)                  ; mapped 4->4


; For UPDRS - Item 2: Hallucinations and psychosis in M (#2, 1.2) and Thought disorder in U (#2)
IF(MODEL.EQ.GR4.AND.ENDPT.EQ.2.AND.ITEM.EQ.2.AND.DV.EQ.0) P = P0*FR0              ; mapped 0->0
IF(MODEL.EQ.GR4.AND.ENDPT.EQ.2.AND.ITEM.EQ.2.AND.DV.EQ.1) P = P0*(1-FR0)          ; mapped 1->0
IF(MODEL.EQ.GR4.AND.ENDPT.EQ.2.AND.ITEM.EQ.2.AND.DV.EQ.2) P = P1 + P2             ; mapped 2->1|2
IF(MODEL.EQ.GR4.AND.ENDPT.EQ.2.AND.ITEM.EQ.2.AND.DV.EQ.3) P = P3                  ; mapped 3->3
IF(MODEL.EQ.GR4.AND.ENDPT.EQ.2.AND.ITEM.EQ.2.AND.DV.EQ.4) P = P4                  ; mapped 4->4
IF(MODEL.EQ.GR4.AND.ENDPT.EQ.2.AND.ITEM.EQ.2) UP0 = P0*FR0                        ; mapped 0->0
IF(MODEL.EQ.GR4.AND.ENDPT.EQ.2.AND.ITEM.EQ.2) UP1 = P0*(1-FR0)                    ; mapped 1->0
IF(MODEL.EQ.GR4.AND.ENDPT.EQ.2.AND.ITEM.EQ.2) UP2 = P1 + P2                       ; mapped 2->1|2
IF(MODEL.EQ.GR4.AND.ENDPT.EQ.2.AND.ITEM.EQ.2) UP3 = P3                            ; mapped 3->3
IF(MODEL.EQ.GR4.AND.ENDPT.EQ.2.AND.ITEM.EQ.2) UP4 = P4                            ; mapped 4->4


;For UPDRS - Item 3 : Depressed mood in M (#3, 1.3) and Depression in U (#3)
IF(MODEL.EQ.GR4.AND.ENDPT.EQ.2.AND.ITEM.EQ.3.AND.DV.EQ.0) P = P0                  ; mapped 0->0
IF(MODEL.EQ.GR4.AND.ENDPT.EQ.2.AND.ITEM.EQ.3.AND.DV.EQ.1) P = P1                  ; mapped 1->1
IF(MODEL.EQ.GR4.AND.ENDPT.EQ.2.AND.ITEM.EQ.3.AND.DV.EQ.2) P = P2                  ; mapped 2->2
IF(MODEL.EQ.GR4.AND.ENDPT.EQ.2.AND.ITEM.EQ.3.AND.DV.EQ.3) P = P3                  ; mapped 3->3
IF(MODEL.EQ.GR4.AND.ENDPT.EQ.2.AND.ITEM.EQ.3.AND.DV.EQ.4) P = P4                  ; mapped 4->4
IF(MODEL.EQ.GR4.AND.ENDPT.EQ.2.AND.ITEM.EQ.3) UP0 = P0                            ; mapped 0->0
IF(MODEL.EQ.GR4.AND.ENDPT.EQ.2.AND.ITEM.EQ.3) UP1 = P1                            ; mapped 1->1
IF(MODEL.EQ.GR4.AND.ENDPT.EQ.2.AND.ITEM.EQ.3) UP2 = P2                            ; mapped 2->2
IF(MODEL.EQ.GR4.AND.ENDPT.EQ.2.AND.ITEM.EQ.3) UP3 = P3                            ; mapped 3->3
IF(MODEL.EQ.GR4.AND.ENDPT.EQ.2.AND.ITEM.EQ.3) UP4 = P4                            ; mapped 4->4


;For UPDRS - Item 4: Apathy in M (#5, 1.5) and Motivation/Initiative in U (#4)
IF(MODEL.EQ.GR4.AND.ENDPT.EQ.2.AND.ITEM.EQ.4.AND.DV.EQ.0) P = P0                  ; mapped 0->0
IF(MODEL.EQ.GR4.AND.ENDPT.EQ.2.AND.ITEM.EQ.4.AND.DV.EQ.1) P = P1                  ; mapped 1->1
IF(MODEL.EQ.GR4.AND.ENDPT.EQ.2.AND.ITEM.EQ.4.AND.DV.EQ.2) P = P2                  ; mapped 2->2
IF(MODEL.EQ.GR4.AND.ENDPT.EQ.2.AND.ITEM.EQ.4.AND.DV.EQ.3) P = P3                  ; mapped 3->3
IF(MODEL.EQ.GR4.AND.ENDPT.EQ.2.AND.ITEM.EQ.4.AND.DV.EQ.4) P = P4                  ; mapped 4->4
IF(MODEL.EQ.GR4.AND.ENDPT.EQ.2.AND.ITEM.EQ.4) UP0 = P0                            ; mapped 0->0
IF(MODEL.EQ.GR4.AND.ENDPT.EQ.2.AND.ITEM.EQ.4) UP1 = P1                            ; mapped 1->1
IF(MODEL.EQ.GR4.AND.ENDPT.EQ.2.AND.ITEM.EQ.4) UP2 = P2                            ; mapped 2->2
IF(MODEL.EQ.GR4.AND.ENDPT.EQ.2.AND.ITEM.EQ.4) UP3 = P3                            ; mapped 3->3
IF(MODEL.EQ.GR4.AND.ENDPT.EQ.2.AND.ITEM.EQ.4) UP4 = P4                            ; mapped 4->4


;For UPDRS - Item 5: Speech (ADL) in M (#14, 2.1) and Speech (ADL) in U (#5)
IF(MODEL.EQ.GR4.AND.ENDPT.EQ.2.AND.ITEM.EQ.5.AND.DV.EQ.0) P = P0                  ; mapped 0->0
IF(MODEL.EQ.GR4.AND.ENDPT.EQ.2.AND.ITEM.EQ.5.AND.DV.EQ.1) P = P1                  ; mapped 1->1
IF(MODEL.EQ.GR4.AND.ENDPT.EQ.2.AND.ITEM.EQ.5.AND.DV.EQ.2) P = P2                  ; mapped 2->2
IF(MODEL.EQ.GR4.AND.ENDPT.EQ.2.AND.ITEM.EQ.5.AND.DV.EQ.3) P = P3                  ; mapped 3->3
IF(MODEL.EQ.GR4.AND.ENDPT.EQ.2.AND.ITEM.EQ.5.AND.DV.EQ.4) P = P4                  ; mapped 4->4
IF(MODEL.EQ.GR4.AND.ENDPT.EQ.2.AND.ITEM.EQ.5) UP0 = P0                            ; mapped 0->0
IF(MODEL.EQ.GR4.AND.ENDPT.EQ.2.AND.ITEM.EQ.5) UP1 = P1                            ; mapped 1->1
IF(MODEL.EQ.GR4.AND.ENDPT.EQ.2.AND.ITEM.EQ.5) UP2 = P2                            ; mapped 2->2
```

```
IF(MODEL.EQ.GR4.AND.ENDPT.EQ.2.AND.ITEM.EQ.5) UP3 = P3                          ; mapped 3->3
IF(MODEL.EQ.GR4.AND.ENDPT.EQ.2.AND.ITEM.EQ.5) UP4 = P4                          ; mapped 4->4


; For UPDRS - Item 6: Saliva and drooling in M (#15, 2.2) and Salivation in U (#6) respectively
IF(MODEL.EQ.GR4.AND.ENDPT.EQ.2.AND.ITEM.EQ.6.AND.DV.EQ.0) P = P0 + P1*FR1        ; mapped 0->0
IF(MODEL.EQ.GR4.AND.ENDPT.EQ.2.AND.ITEM.EQ.6.AND.DV.EQ.1) P = P1*(1-FR1) + P2   ; mapped 1->2
IF(MODEL.EQ.GR4.AND.ENDPT.EQ.2.AND.ITEM.EQ.6.AND.DV.EQ.2) P = P3*FR3             ; mapped 2->3
IF(MODEL.EQ.GR4.AND.ENDPT.EQ.2.AND.ITEM.EQ.6.AND.DV.EQ.3) P = P3*(1-FR3)         ; mapped 3->3
IF(MODEL.EQ.GR4.AND.ENDPT.EQ.2.AND.ITEM.EQ.6.AND.DV.EQ.4) P = P4                 ; mapped 4->4
IF(MODEL.EQ.GR4.AND.ENDPT.EQ.2.AND.ITEM.EQ.6) UP0 = P0 + P1*FR1                  ; mapped 0->0
IF(MODEL.EQ.GR4.AND.ENDPT.EQ.2.AND.ITEM.EQ.6) UP1 = P1*(1-FR1) + P2             ; mapped 1->2
IF(MODEL.EQ.GR4.AND.ENDPT.EQ.2.AND.ITEM.EQ.6) UP2 = P3*FR3                       ; mapped 2->3
IF(MODEL.EQ.GR4.AND.ENDPT.EQ.2.AND.ITEM.EQ.6) UP3 = P3*(1-FR3)                   ; mapped 3->3
IF(MODEL.EQ.GR4.AND.ENDPT.EQ.2.AND.ITEM.EQ.6) UP4 = P4                           ; mapped 4->4


;For UPDRS - Item 7 : Chewing and Swallowing in M (#16, 2.3) and Swallowing in U (#7)
IF(MODEL.EQ.GR4.AND.ENDPT.EQ.2.AND.ITEM.EQ.7.AND.DV.EQ.0) P = P0 + P1*FR1                ; mapped 0->0
IF(MODEL.EQ.GR4.AND.ENDPT.EQ.2.AND.ITEM.EQ.7.AND.DV.EQ.1) P = P1*(1-FR1) + P3*FR3       ; mapped 1->3
IF(MODEL.EQ.GR4.AND.ENDPT.EQ.2.AND.ITEM.EQ.7.AND.DV.EQ.2) P = P3*(1-FR3)                 ; mapped 2->3
IF(MODEL.EQ.GR4.AND.ENDPT.EQ.2.AND.ITEM.EQ.7.AND.DV.EQ.3) P = P2                         ; mapped 3->2
IF(MODEL.EQ.GR4.AND.ENDPT.EQ.2.AND.ITEM.EQ.7.AND.DV.EQ.4) P = P4                         ; mapped 4->4
IF(MODEL.EQ.GR4.AND.ENDPT.EQ.2.AND.ITEM.EQ.7) UP0 = P0 + P1*FR1                          ; mapped 0->0
IF(MODEL.EQ.GR4.AND.ENDPT.EQ.2.AND.ITEM.EQ.7) UP1 = P1*(1-FR1) + P3*FR3                 ; mapped 1->3
IF(MODEL.EQ.GR4.AND.ENDPT.EQ.2.AND.ITEM.EQ.7) UP2 = P3*(1-FR3)                           ; mapped 2->3
IF(MODEL.EQ.GR4.AND.ENDPT.EQ.2.AND.ITEM.EQ.7) UP3 = P2                                   ; mapped 3->2
IF(MODEL.EQ.GR4.AND.ENDPT.EQ.2.AND.ITEM.EQ.7) UP4 = P4                                   ; mapped 4->4


;For UPDRS - Item 8: Handwriting in M (#20, 2.7) and Handwriting in U (#8)
IF(MODEL.EQ.GR4.AND.ENDPT.EQ.2.AND.ITEM.EQ.8.AND.DV.EQ.0) P = P0                 ; mapped 0->0
IF(MODEL.EQ.GR4.AND.ENDPT.EQ.2.AND.ITEM.EQ.8.AND.DV.EQ.1) P = P1*FR1             ; mapped 1->1
IF(MODEL.EQ.GR4.AND.ENDPT.EQ.2.AND.ITEM.EQ.8.AND.DV.EQ.2) P = P1*(1-FR1)         ; mapped 2->1
IF(MODEL.EQ.GR4.AND.ENDPT.EQ.2.AND.ITEM.EQ.8.AND.DV.EQ.3) P = P2 + P3            ; mapped 3->2|3
IF(MODEL.EQ.GR4.AND.ENDPT.EQ.2.AND.ITEM.EQ.8.AND.DV.EQ.4) P = P4                 ; mapped 4->4
IF(MODEL.EQ.GR4.AND.ENDPT.EQ.2.AND.ITEM.EQ.8) UP0 = P0                           ; mapped 0->0
IF(MODEL.EQ.GR4.AND.ENDPT.EQ.2.AND.ITEM.EQ.8) UP1 = P1*FR1                       ; mapped 1->1
IF(MODEL.EQ.GR4.AND.ENDPT.EQ.2.AND.ITEM.EQ.8) UP2 = P1*(1-FR1)                   ; mapped 2->1
IF(MODEL.EQ.GR4.AND.ENDPT.EQ.2.AND.ITEM.EQ.8) UP3 = P2 + P3                      ; mapped 3->2|3
IF(MODEL.EQ.GR4.AND.ENDPT.EQ.2.AND.ITEM.EQ.8) UP4 = P4                           ; mapped 4->4


;For UPDRS - Eating tasks in M (#17, 2.4) and Cutting food and handling utensils in U (#9)
IF(MODEL.EQ.GR4.AND.ENDPT.EQ.2.AND.ITEM.EQ.9.AND.DV.EQ.0) P = P0                 ; mapped 0->0
IF(MODEL.EQ.GR4.AND.ENDPT.EQ.2.AND.ITEM.EQ.9.AND.DV.EQ.1) P = P1                 ; mapped 1->1
IF(MODEL.EQ.GR4.AND.ENDPT.EQ.2.AND.ITEM.EQ.9.AND.DV.EQ.2) P = P2                 ; mapped 2->2
IF(MODEL.EQ.GR4.AND.ENDPT.EQ.2.AND.ITEM.EQ.9.AND.DV.EQ.3) P = P3                 ; mapped 3->3
IF(MODEL.EQ.GR4.AND.ENDPT.EQ.2.AND.ITEM.EQ.9.AND.DV.EQ.4) P = P4                 ; mapped 4->4
IF(MODEL.EQ.GR4.AND.ENDPT.EQ.2.AND.ITEM.EQ.9) UP0 = P0                           ; mapped 0->0
IF(MODEL.EQ.GR4.AND.ENDPT.EQ.2.AND.ITEM.EQ.9) UP1 = P1                           ; mapped 1->1
IF(MODEL.EQ.GR4.AND.ENDPT.EQ.2.AND.ITEM.EQ.9) UP2 = P2                           ; mapped 2->2
IF(MODEL.EQ.GR4.AND.ENDPT.EQ.2.AND.ITEM.EQ.9) UP3 = P3                           ; mapped 3->3
IF(MODEL.EQ.GR4.AND.ENDPT.EQ.2.AND.ITEM.EQ.9) UP4 = P4                           ; mapped 4->4
```

```
For UPDRS - Item 10: Dressing in M (#18, 2.5) and Dressing in U (#10)
IF(MODEL.EQ.GR4.AND.ENDPT.EQ.2.AND.ITEM.EQ.10.AND.DV.EQ.0) P = P0                 ; mapped 0->0
IF(MODEL.EQ.GR4.AND.ENDPT.EQ.2.AND.ITEM.EQ.10.AND.DV.EQ.1) P = P1                 ; mapped 1->1
IF(MODEL.EQ.GR4.AND.ENDPT.EQ.2.AND.ITEM.EQ.10.AND.DV.EQ.2) P = P2                 ; mapped 2->2
IF(MODEL.EQ.GR4.AND.ENDPT.EQ.2.AND.ITEM.EQ.10.AND.DV.EQ.3) P = P3                 ; mapped 3->3
IF(MODEL.EQ.GR4.AND.ENDPT.EQ.2.AND.ITEM.EQ.10.AND.DV.EQ.4) P = P4                 ; mapped 4->4
IF(MODEL.EQ.GR4.AND.ENDPT.EQ.2.AND.ITEM.EQ.10) UP0 = P0                           ; mapped 0->0
IF(MODEL.EQ.GR4.AND.ENDPT.EQ.2.AND.ITEM.EQ.10) UP1 = P1                           ; mapped 1->1
IF(MODEL.EQ.GR4.AND.ENDPT.EQ.2.AND.ITEM.EQ.10) UP2 = P2                           ; mapped 2->2
IF(MODEL.EQ.GR4.AND.ENDPT.EQ.2.AND.ITEM.EQ.10) UP3 = P3                           ; mapped 3->3
IF(MODEL.EQ.GR4.AND.ENDPT.EQ.2.AND.ITEM.EQ.10) UP4 = P4                           ; mapped 4->4


;For UPDRS - Item 11: Hygiene in M (#19, 2.6) and Hygiene in U (#11)
IF(MODEL.EQ.GR4.AND.ENDPT.EQ.2.AND.ITEM.EQ.11.AND.DV.EQ.0) P = P0                 ; mapped 0->0
IF(MODEL.EQ.GR4.AND.ENDPT.EQ.2.AND.ITEM.EQ.11.AND.DV.EQ.1) P = P1                 ; mapped 1->1
IF(MODEL.EQ.GR4.AND.ENDPT.EQ.2.AND.ITEM.EQ.11.AND.DV.EQ.2) P = P2                 ; mapped 2->2
IF(MODEL.EQ.GR4.AND.ENDPT.EQ.2.AND.ITEM.EQ.11.AND.DV.EQ.3) P = P3                 ; mapped 3->3
IF(MODEL.EQ.GR4.AND.ENDPT.EQ.2.AND.ITEM.EQ.11.AND.DV.EQ.4) P = P4                 ; mapped 4->4
IF(MODEL.EQ.GR4.AND.ENDPT.EQ.2.AND.ITEM.EQ.11) UP0 = P0                           ; mapped 0->0
IF(MODEL.EQ.GR4.AND.ENDPT.EQ.2.AND.ITEM.EQ.11) UP1 = P1                           ; mapped 1->1
IF(MODEL.EQ.GR4.AND.ENDPT.EQ.2.AND.ITEM.EQ.11) UP2 = P2                           ; mapped 2->2
IF(MODEL.EQ.GR4.AND.ENDPT.EQ.2.AND.ITEM.EQ.11) UP3 = P3                           ; mapped 3->3
IF(MODEL.EQ.GR4.AND.ENDPT.EQ.2.AND.ITEM.EQ.11) UP4 = P4                           ; mapped 4->4


;For UPDRS - Item 12: Turning in bed in M (#22, 2.9) and Turning in bed and adjusting clothes in U (#12)
IF(MODEL.EQ.GR4.AND.ENDPT.EQ.2.AND.ITEM.EQ.12.AND.DV.EQ.0) P = P0                           ; mapped 0->0
IF(MODEL.EQ.GR4.AND.ENDPT.EQ.2.AND.ITEM.EQ.12.AND.DV.EQ.1) P = P1                           ; mapped 1->1
IF(MODEL.EQ.GR4.AND.ENDPT.EQ.2.AND.ITEM.EQ.12.AND.DV.EQ.2) P = P2 + P3*FR3                  ; mapped 2->2|3
IF(MODEL.EQ.GR4.AND.ENDPT.EQ.2.AND.ITEM.EQ.12.AND.DV.EQ.3) P = P3*(1-FR3) + P4*FR4          ; mapped 3->3|4
IF(MODEL.EQ.GR4.AND.ENDPT.EQ.2.AND.ITEM.EQ.12.AND.DV.EQ.4) P = P4*(1-FR4)                   ; mapped 4->4
IF(MODEL.EQ.GR4.AND.ENDPT.EQ.2.AND.ITEM.EQ.12) UP0 = P0                                     ; mapped 0->0
IF(MODEL.EQ.GR4.AND.ENDPT.EQ.2.AND.ITEM.EQ.12) UP1 = P1                                     ; mapped 1->1
IF(MODEL.EQ.GR4.AND.ENDPT.EQ.2.AND.ITEM.EQ.12) UP2 = P2 + P3*FR3                            ; mapped 2->2|3
IF(MODEL.EQ.GR4.AND.ENDPT.EQ.2.AND.ITEM.EQ.12) UP3 = P3*(1-FR3) + P4*FR4                    ; mapped 3->3|4
IF(MODEL.EQ.GR4.AND.ENDPT.EQ.2.AND.ITEM.EQ.12) UP4 = P4*(1-FR4)                             ; mapped 4->4


;For UPDRS - Item 13: Falling in U (#13) - ONLY in U
IF(MODEL.EQ.GR4.AND.ENDPT.EQ.2.AND.ITEM.EQ.13.AND.DV.EQ.0) P = P0
IF(MODEL.EQ.GR4.AND.ENDPT.EQ.2.AND.ITEM.EQ.13.AND.DV.EQ.1) P = P1
IF(MODEL.EQ.GR4.AND.ENDPT.EQ.2.AND.ITEM.EQ.13.AND.DV.EQ.2) P = P2
IF(MODEL.EQ.GR4.AND.ENDPT.EQ.2.AND.ITEM.EQ.13.AND.DV.EQ.3) P = P3
IF(MODEL.EQ.GR4.AND.ENDPT.EQ.2.AND.ITEM.EQ.13.AND.DV.EQ.4) P = P4
IF(MODEL.EQ.GR4.AND.ENDPT.EQ.2.AND.ITEM.EQ.13) UP0 = P0
IF(MODEL.EQ.GR4.AND.ENDPT.EQ.2.AND.ITEM.EQ.13) UP1 = P1
IF(MODEL.EQ.GR4.AND.ENDPT.EQ.2.AND.ITEM.EQ.13) UP2 = P2
IF(MODEL.EQ.GR4.AND.ENDPT.EQ.2.AND.ITEM.EQ.13) UP3 = P3
IF(MODEL.EQ.GR4.AND.ENDPT.EQ.2.AND.ITEM.EQ.13) UP4 = P4
```

```
;For UPDRS -  Item 14: Freezing in M (#26, 2.13) and Freezing when walking in U (#14)
IF(MODEL.EQ.GR4.AND.ENDPT.EQ.2.AND.ITEM.EQ.14.AND.DV.EQ.0) P = P0
IF(MODEL.EQ.GR4.AND.ENDPT.EQ.2.AND.ITEM.EQ.14.AND.DV.EQ.1) P = P1
IF(MODEL.EQ.GR4.AND.ENDPT.EQ.2.AND.ITEM.EQ.14.AND.DV.EQ.2) P = P2
IF(MODEL.EQ.GR4.AND.ENDPT.EQ.2.AND.ITEM.EQ.14.AND.DV.EQ.3) P = P3
IF(MODEL.EQ.GR4.AND.ENDPT.EQ.2.AND.ITEM.EQ.14.AND.DV.EQ.4) P = P4
IF(MODEL.EQ.GR4.AND.ENDPT.EQ.2.AND.ITEM.EQ.14) UP0 = P0
IF(MODEL.EQ.GR4.AND.ENDPT.EQ.2.AND.ITEM.EQ.14) UP1 = P1
IF(MODEL.EQ.GR4.AND.ENDPT.EQ.2.AND.ITEM.EQ.14) UP2 = P2
IF(MODEL.EQ.GR4.AND.ENDPT.EQ.2.AND.ITEM.EQ.14) UP3 = P3
IF(MODEL.EQ.GR4.AND.ENDPT.EQ.2.AND.ITEM.EQ.14) UP4 = P4


;For UPDRS - Item 15: Walking and balance in M (#25, 2.12) and Walking in U (#15)
IF(MODEL.EQ.GR4.AND.ENDPT.EQ.2.AND.ITEM.EQ.15.AND.DV.EQ.0) P = P0                       ; mapped 0->0
IF(MODEL.EQ.GR4.AND.ENDPT.EQ.2.AND.ITEM.EQ.15.AND.DV.EQ.1) P = P1*FR1                   ; mapped 1->1
IF(MODEL.EQ.GR4.AND.ENDPT.EQ.2.AND.ITEM.EQ.15.AND.DV.EQ.2) P = P2 + P1*(1-FR1)          ; mapped 2->1|2
IF(MODEL.EQ.GR4.AND.ENDPT.EQ.2.AND.ITEM.EQ.15.AND.DV.EQ.3) P = P3 + P4*FR4              ; mapped 3->3|4
IF(MODEL.EQ.GR4.AND.ENDPT.EQ.2.AND.ITEM.EQ.15.AND.DV.EQ.4) P = P4*(1-FR4)               ; mapped 4->4
IF(MODEL.EQ.GR4.AND.ENDPT.EQ.2.AND.ITEM.EQ.15) UP0 = P0                                 ; mapped 0->0
IF(MODEL.EQ.GR4.AND.ENDPT.EQ.2.AND.ITEM.EQ.15) UP1 = P1*FR1                             ; mapped 1->1
IF(MODEL.EQ.GR4.AND.ENDPT.EQ.2.AND.ITEM.EQ.15) UP2 = P2 + P1*(1-FR1)                    ; mapped 2->1|2
IF(MODEL.EQ.GR4.AND.ENDPT.EQ.2.AND.ITEM.EQ.15) UP3 = P3 + P4*FR4                        ; mapped 3->3|4
IF(MODEL.EQ.GR4.AND.ENDPT.EQ.2.AND.ITEM.EQ.15) UP4 = P4*(1-FR4)                         ; mapped 4->4


;For UPDRS - Item 16: Tremor (ADL) in M (#23, 2.10) and Tremor (ADL) in U (#16)
IF(MODEL.EQ.GR4.AND.ENDPT.EQ.2.AND.ITEM.EQ.16.AND.DV.EQ.0) P = P0                       ; mapped 0->0
IF(MODEL.EQ.GR4.AND.ENDPT.EQ.2.AND.ITEM.EQ.16.AND.DV.EQ.1) P = P1                       ; mapped 1->1
IF(MODEL.EQ.GR4.AND.ENDPT.EQ.2.AND.ITEM.EQ.16.AND.DV.EQ.2) P = P2                       ; mapped 2->2
IF(MODEL.EQ.GR4.AND.ENDPT.EQ.2.AND.ITEM.EQ.16.AND.DV.EQ.3) P = P3                       ; mapped 3->3
IF(MODEL.EQ.GR4.AND.ENDPT.EQ.2.AND.ITEM.EQ.16.AND.DV.EQ.4) P = P4                       ; mapped 4->4
IF(MODEL.EQ.GR4.AND.ENDPT.EQ.2.AND.ITEM.EQ.16) UP0 = P0                                 ; mapped 0->0
IF(MODEL.EQ.GR4.AND.ENDPT.EQ.2.AND.ITEM.EQ.16) UP1 = P1                                 ; mapped 1->1
IF(MODEL.EQ.GR4.AND.ENDPT.EQ.2.AND.ITEM.EQ.16) UP2 = P2                                 ; mapped 2->2
IF(MODEL.EQ.GR4.AND.ENDPT.EQ.2.AND.ITEM.EQ.16) UP3 = P3                                 ; mapped 3->3
IF(MODEL.EQ.GR4.AND.ENDPT.EQ.2.AND.ITEM.EQ.16) UP4 = P4                                 ; mapped 4->4


;For UPDRS - Item 17: Pain and other sensations in M (#9, 1.9) and Sensory complaints related to PD in U (#17)
IF(MODEL.EQ.GR4.AND.ENDPT.EQ.2.AND.ITEM.EQ.17.AND.DV.EQ.0) P = P0                       ; mapped 0->0
IF(MODEL.EQ.GR4.AND.ENDPT.EQ.2.AND.ITEM.EQ.17.AND.DV.EQ.1) P = P1                       ; mapped 1->1
IF(MODEL.EQ.GR4.AND.ENDPT.EQ.2.AND.ITEM.EQ.17.AND.DV.EQ.2) P = P2                       ; mapped 2->2
IF(MODEL.EQ.GR4.AND.ENDPT.EQ.2.AND.ITEM.EQ.17.AND.DV.EQ.3) P = P3                       ; mapped 3->3
IF(MODEL.EQ.GR4.AND.ENDPT.EQ.2.AND.ITEM.EQ.17.AND.DV.EQ.4) P = P4                       ; mapped 4->4
IF(MODEL.EQ.GR4.AND.ENDPT.EQ.2.AND.ITEM.EQ.17) UP0 = P0                                 ; mapped 0->0
IF(MODEL.EQ.GR4.AND.ENDPT.EQ.2.AND.ITEM.EQ.17) UP1 = P1                                 ; mapped 1->1
IF(MODEL.EQ.GR4.AND.ENDPT.EQ.2.AND.ITEM.EQ.17) UP2 = P2                                 ; mapped 2->2
IF(MODEL.EQ.GR4.AND.ENDPT.EQ.2.AND.ITEM.EQ.17) UP3 = P3                                 ; mapped 3->3
IF(MODEL.EQ.GR4.AND.ENDPT.EQ.2.AND.ITEM.EQ.17) UP4 = P4                                 ; mapped 4->4
```

```
;For UPDRS - Item 18: Speech (motor examination) in M (#27, 3.1) and Speech (motor examination) in U (#18)
IF(MODEL.EQ.GR4.AND.ENDPT.EQ.2.AND.ITEM.EQ.18.AND.DV.EQ.0) P = P0                  ; mapped 0->0
IF(MODEL.EQ.GR4.AND.ENDPT.EQ.2.AND.ITEM.EQ.18.AND.DV.EQ.1) P = P1                  ; mapped 1->1
IF(MODEL.EQ.GR4.AND.ENDPT.EQ.2.AND.ITEM.EQ.18.AND.DV.EQ.2) P = P2                  ; mapped 2->2
IF(MODEL.EQ.GR4.AND.ENDPT.EQ.2.AND.ITEM.EQ.18.AND.DV.EQ.3) P = P3 + P4*FR4         ; mapped 3->3|4
IF(MODEL.EQ.GR4.AND.ENDPT.EQ.2.AND.ITEM.EQ.18.AND.DV.EQ.4) P = P4*(1-FR4)          ; mapped 4->4
IF(MODEL.EQ.GR4.AND.ENDPT.EQ.2.AND.ITEM.EQ.18) UP0 = P0                            ; mapped 0->0
IF(MODEL.EQ.GR4.AND.ENDPT.EQ.2.AND.ITEM.EQ.18) UP1 = P1                            ; mapped 1->1
IF(MODEL.EQ.GR4.AND.ENDPT.EQ.2.AND.ITEM.EQ.18) UP2 = P2                            ; mapped 2->2
IF(MODEL.EQ.GR4.AND.ENDPT.EQ.2.AND.ITEM.EQ.18) UP3 = P3 + P4*FR4                   ; mapped 3->3|4
IF(MODEL.EQ.GR4.AND.ENDPT.EQ.2.AND.ITEM.EQ.18) UP4 = P4*(1-FR4)                    ; mapped 4->4


;For UPDRS - Item 19: Facial expression in M (#28, 3.2) and Facial expression in U (#19)
IF(MODEL.EQ.GR4.AND.ENDPT.EQ.2.AND.ITEM.EQ.19.AND.DV.EQ.0) P = P0                  ; mapped 0->0
IF(MODEL.EQ.GR4.AND.ENDPT.EQ.2.AND.ITEM.EQ.19.AND.DV.EQ.1) P = P1                  ; mapped 1->1
IF(MODEL.EQ.GR4.AND.ENDPT.EQ.2.AND.ITEM.EQ.19.AND.DV.EQ.2) P = P2                  ; mapped 2->2
IF(MODEL.EQ.GR4.AND.ENDPT.EQ.2.AND.ITEM.EQ.19.AND.DV.EQ.3) P = P3                  ; mapped 3->3
IF(MODEL.EQ.GR4.AND.ENDPT.EQ.2.AND.ITEM.EQ.19.AND.DV.EQ.4) P = P4                  ; mapped 4->4
IF(MODEL.EQ.GR4.AND.ENDPT.EQ.2.AND.ITEM.EQ.19) UP0 = P0                            ; mapped 0->0
IF(MODEL.EQ.GR4.AND.ENDPT.EQ.2.AND.ITEM.EQ.19) UP1 = P1                            ; mapped 1->1
IF(MODEL.EQ.GR4.AND.ENDPT.EQ.2.AND.ITEM.EQ.19) UP2 = P2                            ; mapped 2->2
IF(MODEL.EQ.GR4.AND.ENDPT.EQ.2.AND.ITEM.EQ.19) UP3 = P3                            ; mapped 3->3
IF(MODEL.EQ.GR4.AND.ENDPT.EQ.2.AND.ITEM.EQ.19) UP4 = P4                            ; mapped 4->4


;For UPDRS - Item 200: Rest tremor amplitude - Face lips, chin in M (#58, 3.17e)
; and Tremor at rest - Face lips, chin in U (#200)
; MDS part coded as Item 46 (after all the U code)
IF(MODEL.EQ.GR4.AND.ENDPT.EQ.2.AND.ITEM.EQ.200.AND.DV.EQ.0) P = P0
IF(MODEL.EQ.GR4.AND.ENDPT.EQ.2.AND.ITEM.EQ.200.AND.DV.EQ.1) P = P1
IF(MODEL.EQ.GR4.AND.ENDPT.EQ.2.AND.ITEM.EQ.200.AND.DV.EQ.2) P = P2
IF(MODEL.EQ.GR4.AND.ENDPT.EQ.2.AND.ITEM.EQ.200.AND.DV.EQ.3) P = P3
IF(MODEL.EQ.GR4.AND.ENDPT.EQ.2.AND.ITEM.EQ.200.AND.DV.EQ.4) P = P4
IF(MODEL.EQ.GR4.AND.ENDPT.EQ.2.AND.ITEM.EQ.200) UP0 = P0
IF(MODEL.EQ.GR4.AND.ENDPT.EQ.2.AND.ITEM.EQ.200) UP1 = P1
IF(MODEL.EQ.GR4.AND.ENDPT.EQ.2.AND.ITEM.EQ.200) UP2 = P2
IF(MODEL.EQ.GR4.AND.ENDPT.EQ.2.AND.ITEM.EQ.200) UP3 = P3
IF(MODEL.EQ.GR4.AND.ENDPT.EQ.2.AND.ITEM.EQ.200) UP4 = P4


;For UPDRS - ; Item 201: Rest tremor amplitude - Left UE in M (#55, 3.17b)
; and Tremor at rest - Left hand in U (#201)
; MDS part coded as Item 47 (after all the U code)
IF(MODEL.EQ.GR4.AND.ENDPT.EQ.2.AND.ITEM.EQ.201.AND.DV.EQ.0) P = P0
IF(MODEL.EQ.GR4.AND.ENDPT.EQ.2.AND.ITEM.EQ.201.AND.DV.EQ.1) P = P1
IF(MODEL.EQ.GR4.AND.ENDPT.EQ.2.AND.ITEM.EQ.201.AND.DV.EQ.2) P = P2
IF(MODEL.EQ.GR4.AND.ENDPT.EQ.2.AND.ITEM.EQ.201.AND.DV.EQ.3) P = P3
IF(MODEL.EQ.GR4.AND.ENDPT.EQ.2.AND.ITEM.EQ.201.AND.DV.EQ.4) P = P4
IF(MODEL.EQ.GR4.AND.ENDPT.EQ.2.AND.ITEM.EQ.201) UP0 = P0
IF(MODEL.EQ.GR4.AND.ENDPT.EQ.2.AND.ITEM.EQ.201) UP1 = P1
IF(MODEL.EQ.GR4.AND.ENDPT.EQ.2.AND.ITEM.EQ.201) UP2 = P2
IF(MODEL.EQ.GR4.AND.ENDPT.EQ.2.AND.ITEM.EQ.201) UP3 = P3
IF(MODEL.EQ.GR4.AND.ENDPT.EQ.2.AND.ITEM.EQ.201) UP4 = P4
```

```
;For UPDRS -   Item 202: Rest tremor amplitude - Right UE in M (#54, 3.17a)
; and Tremor at rest - Right hand in U (#202) - NO PARALLELISM
; MDS part coded as Item 48 (after all the U code)
IF(MODEL.EQ.GR4.AND.ENDPT.EQ.2.AND.ITEM.EQ.202.AND.DV.EQ.0) P = P0
IF(MODEL.EQ.GR4.AND.ENDPT.EQ.2.AND.ITEM.EQ.202.AND.DV.EQ.1) P = P1
IF(MODEL.EQ.GR4.AND.ENDPT.EQ.2.AND.ITEM.EQ.202.AND.DV.EQ.2) P = P2
IF(MODEL.EQ.GR4.AND.ENDPT.EQ.2.AND.ITEM.EQ.202.AND.DV.EQ.3) P = P3
IF(MODEL.EQ.GR4.AND.ENDPT.EQ.2.AND.ITEM.EQ.202.AND.DV.EQ.4) P = P4
IF(MODEL.EQ.GR4.AND.ENDPT.EQ.2.AND.ITEM.EQ.202) UP0 = P0
IF(MODEL.EQ.GR4.AND.ENDPT.EQ.2.AND.ITEM.EQ.202) UP1 = P1
IF(MODEL.EQ.GR4.AND.ENDPT.EQ.2.AND.ITEM.EQ.202) UP2 = P2
IF(MODEL.EQ.GR4.AND.ENDPT.EQ.2.AND.ITEM.EQ.202) UP3 = P3
IF(MODEL.EQ.GR4.AND.ENDPT.EQ.2.AND.ITEM.EQ.202) UP4 = P4


;For UPDRS - Item 203: Rest tremor amplitude - Left LE in M (#57, 3.17d)
; and Tremor at rest - Left foot in U (#203) - NO PARALLELISM
; MDS part coded as Item 49 (after all the U code)
IF(MODEL.EQ.GR4.AND.ENDPT.EQ.2.AND.ITEM.EQ.203.AND.DV.EQ.0) P = P0
IF(MODEL.EQ.GR4.AND.ENDPT.EQ.2.AND.ITEM.EQ.203.AND.DV.EQ.1) P = P1
IF(MODEL.EQ.GR4.AND.ENDPT.EQ.2.AND.ITEM.EQ.203.AND.DV.EQ.2) P = P2
IF(MODEL.EQ.GR4.AND.ENDPT.EQ.2.AND.ITEM.EQ.203.AND.DV.EQ.3) P = P3
IF(MODEL.EQ.GR4.AND.ENDPT.EQ.2.AND.ITEM.EQ.203.AND.DV.EQ.4) P = P4
IF(MODEL.EQ.GR4.AND.ENDPT.EQ.2.AND.ITEM.EQ.203) UP0 = P0
IF(MODEL.EQ.GR4.AND.ENDPT.EQ.2.AND.ITEM.EQ.203) UP1 = P1
IF(MODEL.EQ.GR4.AND.ENDPT.EQ.2.AND.ITEM.EQ.203) UP2 = P2
IF(MODEL.EQ.GR4.AND.ENDPT.EQ.2.AND.ITEM.EQ.203) UP3 = P3
IF(MODEL.EQ.GR4.AND.ENDPT.EQ.2.AND.ITEM.EQ.203) UP4 = P4


;For UPDRS - Item 204: Rest tremor amplitude - Right LE in M (#56, 3.17c)
; and Tremor at rest - Right foot  in U (#204) - NO PARALLELISM
; MDS part coded as Item 50 (after all the U code)
IF(MODEL.EQ.GR4.AND.ENDPT.EQ.2.AND.ITEM.EQ.204.AND.DV.EQ.0) P = P0
IF(MODEL.EQ.GR4.AND.ENDPT.EQ.2.AND.ITEM.EQ.204.AND.DV.EQ.1) P = P1
IF(MODEL.EQ.GR4.AND.ENDPT.EQ.2.AND.ITEM.EQ.204.AND.DV.EQ.2) P = P2
IF(MODEL.EQ.GR4.AND.ENDPT.EQ.2.AND.ITEM.EQ.204.AND.DV.EQ.3) P = P3
IF(MODEL.EQ.GR4.AND.ENDPT.EQ.2.AND.ITEM.EQ.204.AND.DV.EQ.4) P = P4
IF(MODEL.EQ.GR4.AND.ENDPT.EQ.2.AND.ITEM.EQ.204) UP0 = P0
IF(MODEL.EQ.GR4.AND.ENDPT.EQ.2.AND.ITEM.EQ.204) UP1 = P1
IF(MODEL.EQ.GR4.AND.ENDPT.EQ.2.AND.ITEM.EQ.204) UP2 = P2
IF(MODEL.EQ.GR4.AND.ENDPT.EQ.2.AND.ITEM.EQ.204) UP3 = P3
IF(MODEL.EQ.GR4.AND.ENDPT.EQ.2.AND.ITEM.EQ.204) UP4 = P4


;For UPDRS - Item 205: Action/Postural tremor  - Left hand in U (#205) - NO PARALLELISM
; MDS part for Postural (51, 3.15b) and Kinetic tremor (53, 3.16b) for left hand
;  -- each coded as Items 51 and 52 (after U part) respectively
IF(MODEL.EQ.GR4.AND.ENDPT.EQ.2.AND.ITEM.EQ.205.AND.DV.EQ.0) P = P0
IF(MODEL.EQ.GR4.AND.ENDPT.EQ.2.AND.ITEM.EQ.205.AND.DV.EQ.1) P = P1
IF(MODEL.EQ.GR4.AND.ENDPT.EQ.2.AND.ITEM.EQ.205.AND.DV.EQ.2) P =  P2
IF(MODEL.EQ.GR4.AND.ENDPT.EQ.2.AND.ITEM.EQ.205.AND.DV.EQ.3) P = P3
IF(MODEL.EQ.GR4.AND.ENDPT.EQ.2.AND.ITEM.EQ.205.AND.DV.EQ.4) P = P4
IF(MODEL.EQ.GR4.AND.ENDPT.EQ.2.AND.ITEM.EQ.205) UP0 = P0
```

```
IF(MODEL.EQ.GR4.AND.ENDPT.EQ.2.AND.ITEM.EQ.205) UP1 = P1
IF(MODEL.EQ.GR4.AND.ENDPT.EQ.2.AND.ITEM.EQ.205) UP2 = P2
IF(MODEL.EQ.GR4.AND.ENDPT.EQ.2.AND.ITEM.EQ.205) UP3 = P3
IF(MODEL.EQ.GR4.AND.ENDPT.EQ.2.AND.ITEM.EQ.205) UP4 = P4


; Item 206: Action/Postural tremor  - Right hand in U (#206) - NO PARALLELISM
; MDS part for Postural (50, 3.15a) and Kinetic tremor (52, 3.16a) for right hand
;  -- each coded as Items 53 and 54 (after U part) respectively
IF(MODEL.EQ.GR4.AND.ENDPT.EQ.2.AND.ITEM.EQ.206.AND.DV.EQ.0) P = P0
IF(MODEL.EQ.GR4.AND.ENDPT.EQ.2.AND.ITEM.EQ.206.AND.DV.EQ.1) P = P1
IF(MODEL.EQ.GR4.AND.ENDPT.EQ.2.AND.ITEM.EQ.206.AND.DV.EQ.2) P = P2
IF(MODEL.EQ.GR4.AND.ENDPT.EQ.2.AND.ITEM.EQ.206.AND.DV.EQ.3) P = P3
IF(MODEL.EQ.GR4.AND.ENDPT.EQ.2.AND.ITEM.EQ.206.AND.DV.EQ.4) P = P4
IF(MODEL.EQ.GR4.AND.ENDPT.EQ.2.AND.ITEM.EQ.206) UP0 = P0
IF(MODEL.EQ.GR4.AND.ENDPT.EQ.2.AND.ITEM.EQ.206) UP1 = P1
IF(MODEL.EQ.GR4.AND.ENDPT.EQ.2.AND.ITEM.EQ.206) UP2 = P2
IF(MODEL.EQ.GR4.AND.ENDPT.EQ.2.AND.ITEM.EQ.206) UP3 = P3
IF(MODEL.EQ.GR4.AND.ENDPT.EQ.2.AND.ITEM.EQ.206) UP4 = P4


;For UPDRS - Item 207: Rigidity - Neck in M (#29, 3.3a) and Rigidity - Neck in U (#207)
IF(MODEL.EQ.GR4.AND.ENDPT.EQ.2.AND.ITEM.EQ.207.AND.DV.EQ.0) P = P0                          ; mapped 0->0
IF(MODEL.EQ.GR4.AND.ENDPT.EQ.2.AND.ITEM.EQ.207.AND.DV.EQ.1) P = P1                          ; mapped 1->1
IF(MODEL.EQ.GR4.AND.ENDPT.EQ.2.AND.ITEM.EQ.207.AND.DV.EQ.2) P = P2*FR2                      ; mapped 2->2
IF(MODEL.EQ.GR4.AND.ENDPT.EQ.2.AND.ITEM.EQ.207.AND.DV.EQ.3) P = P2*(1-FR2) + P4*FR4  ; mapped 3->2
IF(MODEL.EQ.GR4.AND.ENDPT.EQ.2.AND.ITEM.EQ.207.AND.DV.EQ.4) P = P3 + P4*(1-FR4)         ; mapped 4->3|4
IF(MODEL.EQ.GR4.AND.ENDPT.EQ.2.AND.ITEM.EQ.207) UP0 = P0                                    ; mapped 0->0
IF(MODEL.EQ.GR4.AND.ENDPT.EQ.2.AND.ITEM.EQ.207) UP1 = P1                                    ; mapped 1->1
IF(MODEL.EQ.GR4.AND.ENDPT.EQ.2.AND.ITEM.EQ.207) UP2 = P2*FR2                                ; mapped 2->2
IF(MODEL.EQ.GR4.AND.ENDPT.EQ.2.AND.ITEM.EQ.207) UP3 = P2*(1-FR2) + P4*FR4                   ; mapped 3->2
IF(MODEL.EQ.GR4.AND.ENDPT.EQ.2.AND.ITEM.EQ.207) UP4 = P3 + P4*(1-FR4)                       ; mapped 4->3|4


;For UPDRS - Item 208: Rigidity - Left UE in M (#31, 3.3c) and Rigidity - Left UE in U (#208)
IF(MODEL.EQ.GR4.AND.ENDPT.EQ.2.AND.ITEM.EQ.208.AND.DV.EQ.0) P = P0                          ; mapped 0->0
IF(MODEL.EQ.GR4.AND.ENDPT.EQ.2.AND.ITEM.EQ.208.AND.DV.EQ.1) P = P1                          ; mapped 1->1
IF(MODEL.EQ.GR4.AND.ENDPT.EQ.2.AND.ITEM.EQ.208.AND.DV.EQ.2) P = P2*FR2                      ; mapped 2->2
IF(MODEL.EQ.GR4.AND.ENDPT.EQ.2.AND.ITEM.EQ.208.AND.DV.EQ.3) P = P2*(1-FR2) + P4*FR4  ; mapped 3->2
IF(MODEL.EQ.GR4.AND.ENDPT.EQ.2.AND.ITEM.EQ.208.AND.DV.EQ.4) P = P3 + P4*(1-FR4)         ; mapped 4->3|4
IF(MODEL.EQ.GR4.AND.ENDPT.EQ.2.AND.ITEM.EQ.208) UP0 = P0                                    ; mapped 0->0
IF(MODEL.EQ.GR4.AND.ENDPT.EQ.2.AND.ITEM.EQ.208) UP1 = P1                                    ; mapped 1->1
IF(MODEL.EQ.GR4.AND.ENDPT.EQ.2.AND.ITEM.EQ.208) UP2 = P2*FR2                                ; mapped 2->2
IF(MODEL.EQ.GR4.AND.ENDPT.EQ.2.AND.ITEM.EQ.208) UP3 = P2*(1-FR2) + P4*FR4                   ; mapped 3->2
IF(MODEL.EQ.GR4.AND.ENDPT.EQ.2.AND.ITEM.EQ.208) UP4 = P3 + P4*(1-FR4)                       ; mapped 4->3|4


;For UPDRS - Item 209: Rigidity - Right UE in M (#30, 3.3b) and Rigidity - Right UE  in U (#209)
IF(MODEL.EQ.GR4.AND.ENDPT.EQ.2.AND.ITEM.EQ.209.AND.DV.EQ.0) P = P0                          ; mapped 0->0
IF(MODEL.EQ.GR4.AND.ENDPT.EQ.2.AND.ITEM.EQ.209.AND.DV.EQ.1) P = P1                          ; mapped 1->1
IF(MODEL.EQ.GR4.AND.ENDPT.EQ.2.AND.ITEM.EQ.209.AND.DV.EQ.2) P = P2*FR2                      ; mapped 2->2
IF(MODEL.EQ.GR4.AND.ENDPT.EQ.2.AND.ITEM.EQ.209.AND.DV.EQ.3) P = P2*(1-FR2) + P4*FR4  ; mapped 3->2
IF(MODEL.EQ.GR4.AND.ENDPT.EQ.2.AND.ITEM.EQ.209.AND.DV.EQ.4) P = P3 + P4*(1-FR4)         ; mapped 4->3|4
IF(MODEL.EQ.GR4.AND.ENDPT.EQ.2.AND.ITEM.EQ.209) UP0 = P0                                    ; mapped 0->0
```

```
IF(MODEL.EQ.GR4.AND.ENDPT.EQ.2.AND.ITEM.EQ.209) UP1 = P1                                    ; mapped 1->1
IF(MODEL.EQ.GR4.AND.ENDPT.EQ.2.AND.ITEM.EQ.209) UP2 = P2*FR2                                ; mapped 2->2
IF(MODEL.EQ.GR4.AND.ENDPT.EQ.2.AND.ITEM.EQ.209) UP3 = P2*(1-FR2) + P4*FR4                   ; mapped 3->2
IF(MODEL.EQ.GR4.AND.ENDPT.EQ.2.AND.ITEM.EQ.209) UP4 = P3 + P4*(1-FR4)                       ; mapped 4->3|4


;For UPDRS - Item 210: Rigidity - Left LE in M (#33, 3.3e) and Rigidity - Left LE in U (#210)
IF(MODEL.EQ.GR4.AND.ENDPT.EQ.2.AND.ITEM.EQ.210.AND.DV.EQ.0) P = P0                          ; mapped 0->0
IF(MODEL.EQ.GR4.AND.ENDPT.EQ.2.AND.ITEM.EQ.210.AND.DV.EQ.1) P = P1                          ; mapped 1->1
IF(MODEL.EQ.GR4.AND.ENDPT.EQ.2.AND.ITEM.EQ.210.AND.DV.EQ.2) P = P2*FR2                      ; mapped 2->2
IF(MODEL.EQ.GR4.AND.ENDPT.EQ.2.AND.ITEM.EQ.210.AND.DV.EQ.3) P = P2*(1-FR2) + P4*FR4         ; mapped 3->2
IF(MODEL.EQ.GR4.AND.ENDPT.EQ.2.AND.ITEM.EQ.210.AND.DV.EQ.4) P = P3 + P4*(1-FR4)             ; mapped 4->3|4
IF(MODEL.EQ.GR4.AND.ENDPT.EQ.2.AND.ITEM.EQ.210) UP0 = P0                                    ; mapped 0->0
IF(MODEL.EQ.GR4.AND.ENDPT.EQ.2.AND.ITEM.EQ.210) UP1 = P1                                    ; mapped 1->1
IF(MODEL.EQ.GR4.AND.ENDPT.EQ.2.AND.ITEM.EQ.210) UP2 = P2*FR2                                ; mapped 2->2
IF(MODEL.EQ.GR4.AND.ENDPT.EQ.2.AND.ITEM.EQ.210) UP3 = P2*(1-FR2) + P4*FR4                   ; mapped 3->2
IF(MODEL.EQ.GR4.AND.ENDPT.EQ.2.AND.ITEM.EQ.210) UP4 = P3 + P4*(1-FR4)                       ; mapped 4->3|4


;For UPDRS - Item 211: Rigidity - Right LE in M (#32, 3.3d) and  Rigidity - Right LE in U (#211)
IF(MODEL.EQ.GR4.AND.ENDPT.EQ.2.AND.ITEM.EQ.211.AND.DV.EQ.0) P = P0                          ; mapped 0->0
IF(MODEL.EQ.GR4.AND.ENDPT.EQ.2.AND.ITEM.EQ.211.AND.DV.EQ.1) P = P1                          ; mapped 1->1
IF(MODEL.EQ.GR4.AND.ENDPT.EQ.2.AND.ITEM.EQ.211.AND.DV.EQ.2) P = P2*FR2                      ; mapped 2->2
IF(MODEL.EQ.GR4.AND.ENDPT.EQ.2.AND.ITEM.EQ.211.AND.DV.EQ.3) P = P2*(1-FR2) + P4*FR4         ; mapped 3->2
IF(MODEL.EQ.GR4.AND.ENDPT.EQ.2.AND.ITEM.EQ.211.AND.DV.EQ.4) P = P3 + P4*(1-FR4)             ; mapped 4->3|4
IF(MODEL.EQ.GR4.AND.ENDPT.EQ.2.AND.ITEM.EQ.211) UP0 = P0                                    ; mapped 0->0
IF(MODEL.EQ.GR4.AND.ENDPT.EQ.2.AND.ITEM.EQ.211) UP1 = P1                                    ; mapped 1->1
IF(MODEL.EQ.GR4.AND.ENDPT.EQ.2.AND.ITEM.EQ.211) UP2 = P2*FR2                                ; mapped 2->2
IF(MODEL.EQ.GR4.AND.ENDPT.EQ.2.AND.ITEM.EQ.211) UP3 = P2*(1-FR2) + P4*FR4                   ; mapped 3->2
IF(MODEL.EQ.GR4.AND.ENDPT.EQ.2.AND.ITEM.EQ.211) UP4 = P3 + P4*(1-FR4)                       ; mapped 4->3|4


;For UPDRS - Item 212: Finger taps left in M (#35, 3.4b) and Finger taps left in U (#212)
IF(MODEL.EQ.GR4.AND.ENDPT.EQ.2.AND.ITEM.EQ.212.AND.DV.EQ.0) P = P0                          ; mapped 0->0
IF(MODEL.EQ.GR4.AND.ENDPT.EQ.2.AND.ITEM.EQ.212.AND.DV.EQ.1) P = P1 + P2*FR2                 ; mapped 1->1|2
IF(MODEL.EQ.GR4.AND.ENDPT.EQ.2.AND.ITEM.EQ.212.AND.DV.EQ.2) P = P2*(1-FR2) + P3*FR3         ; mapped 2->2|3
IF(MODEL.EQ.GR4.AND.ENDPT.EQ.2.AND.ITEM.EQ.212.AND.DV.EQ.3) P = P3*(1-FR3)                  ; mapped 3->3
IF(MODEL.EQ.GR4.AND.ENDPT.EQ.2.AND.ITEM.EQ.212.AND.DV.EQ.4) P = P4                          ; mapped 4->4
IF(MODEL.EQ.GR4.AND.ENDPT.EQ.2.AND.ITEM.EQ.212) UP0 = P0                                    ; mapped 0->0
IF(MODEL.EQ.GR4.AND.ENDPT.EQ.2.AND.ITEM.EQ.212) UP1 = P1 + P2*FR2                           ; mapped 1->1|2
IF(MODEL.EQ.GR4.AND.ENDPT.EQ.2.AND.ITEM.EQ.212) UP2 = P2*(1-FR2) + P3*FR3                   ; mapped 2->2|3
IF(MODEL.EQ.GR4.AND.ENDPT.EQ.2.AND.ITEM.EQ.212) UP3 = P3*(1-FR3)                            ; mapped 3->3
IF(MODEL.EQ.GR4.AND.ENDPT.EQ.2.AND.ITEM.EQ.212) UP4 = P4                                    ; mapped 4->4


;For UPDRS - Item 213: Finger taps right in M (#34, 3.4a) and Finger taps left in U (#213)
IF(MODEL.EQ.GR4.AND.ENDPT.EQ.2.AND.ITEM.EQ.213.AND.DV.EQ.0) P = P0                          ; mapped 0->0
IF(MODEL.EQ.GR4.AND.ENDPT.EQ.2.AND.ITEM.EQ.213.AND.DV.EQ.1) P = P1 + P2*FR2                 ; mapped 1->1|2
IF(MODEL.EQ.GR4.AND.ENDPT.EQ.2.AND.ITEM.EQ.213.AND.DV.EQ.2) P = P2*(1-FR2) + P3*FR3         ; mapped 2->2|3
IF(MODEL.EQ.GR4.AND.ENDPT.EQ.2.AND.ITEM.EQ.213.AND.DV.EQ.3) P = P3*(1-FR3)                  ; mapped 3->3
IF(MODEL.EQ.GR4.AND.ENDPT.EQ.2.AND.ITEM.EQ.213.AND.DV.EQ.4) P = P4                          ; mapped 4->4
IF(MODEL.EQ.GR4.AND.ENDPT.EQ.2.AND.ITEM.EQ.213) UP0 = P0                                    ; mapped 0->0
IF(MODEL.EQ.GR4.AND.ENDPT.EQ.2.AND.ITEM.EQ.213) UP1 = P1 + P2*FR2                           ; mapped 1->1|2
IF(MODEL.EQ.GR4.AND.ENDPT.EQ.2.AND.ITEM.EQ.213) UP2 = P2*(1-FR2) + P3*FR3                   ; mapped 2->2|3
IF(MODEL.EQ.GR4.AND.ENDPT.EQ.2.AND.ITEM.EQ.213) UP3 = P3*(1-FR3)                            ; mapped 3->3
```

```
IF(MODEL.EQ.GR4.AND.ENDPT.EQ.2.AND.ITEM.EQ.213) UP4 = P4                                ; mapped 4->4


;For UPDRS - Item 214: Hand movement  - left in M (#37, 3.5b) and Hand movement  in U (#214)
IF(MODEL.EQ.GR4.AND.ENDPT.EQ.2.AND.ITEM.EQ.214.AND.DV.EQ.0) P = P0                      ; mapped 0->0
IF(MODEL.EQ.GR4.AND.ENDPT.EQ.2.AND.ITEM.EQ.214.AND.DV.EQ.1) P = P1 + P2*FR2             ; mapped 1->1|2
IF(MODEL.EQ.GR4.AND.ENDPT.EQ.2.AND.ITEM.EQ.214.AND.DV.EQ.2) P = P2*(1-FR2) + P3*FR3     ; mapped 2->2|3
IF(MODEL.EQ.GR4.AND.ENDPT.EQ.2.AND.ITEM.EQ.214.AND.DV.EQ.3) P = P3*(1-FR3)              ; mapped 3->3
IF(MODEL.EQ.GR4.AND.ENDPT.EQ.2.AND.ITEM.EQ.214.AND.DV.EQ.4) P = P4                      ; mapped 4->4
IF(MODEL.EQ.GR4.AND.ENDPT.EQ.2.AND.ITEM.EQ.214) UP0 = P0                                ; mapped 0->0
IF(MODEL.EQ.GR4.AND.ENDPT.EQ.2.AND.ITEM.EQ.214) UP1 = P1 + P2*FR2                       ; mapped 1->1|2
IF(MODEL.EQ.GR4.AND.ENDPT.EQ.2.AND.ITEM.EQ.214) UP2 = P2*(1-FR2) + P3*FR3               ; mapped 2->2|3
IF(MODEL.EQ.GR4.AND.ENDPT.EQ.2.AND.ITEM.EQ.214) UP3 = P3*(1-FR3)                        ; mapped 3->3
IF(MODEL.EQ.GR4.AND.ENDPT.EQ.2.AND.ITEM.EQ.214) UP4 = P4                                ; mapped 4->4


;For UPDRS -  Item 215: Hand movement  - right in M (#36, 3.5a) and Hand movement  in U (#215)
IF(MODEL.EQ.GR4.AND.ENDPT.EQ.2.AND.ITEM.EQ.215.AND.DV.EQ.0) P = P0                      ; mapped 0->0
IF(MODEL.EQ.GR4.AND.ENDPT.EQ.2.AND.ITEM.EQ.215.AND.DV.EQ.1) P = P1 + P2*FR2             ; mapped 1->1|2
IF(MODEL.EQ.GR4.AND.ENDPT.EQ.2.AND.ITEM.EQ.215.AND.DV.EQ.2) P = P2*(1-FR2) + P3*FR3     ; mapped 2->2|3
IF(MODEL.EQ.GR4.AND.ENDPT.EQ.2.AND.ITEM.EQ.215.AND.DV.EQ.3) P = P3*(1-FR3)              ; mapped 3->3
IF(MODEL.EQ.GR4.AND.ENDPT.EQ.2.AND.ITEM.EQ.215.AND.DV.EQ.4) P = P4                      ; mapped 4->4
IF(MODEL.EQ.GR4.AND.ENDPT.EQ.2.AND.ITEM.EQ.215) UP0 = P0                                ; mapped 0->0
IF(MODEL.EQ.GR4.AND.ENDPT.EQ.2.AND.ITEM.EQ.215) UP1 = P1 + P2*FR2                       ; mapped 1->1|2
IF(MODEL.EQ.GR4.AND.ENDPT.EQ.2.AND.ITEM.EQ.215) UP2 = P2*(1-FR2) + P3*FR3               ; mapped 2->2|3
IF(MODEL.EQ.GR4.AND.ENDPT.EQ.2.AND.ITEM.EQ.215) UP3 = P3*(1-FR3)                        ; mapped 3->3
IF(MODEL.EQ.GR4.AND.ENDPT.EQ.2.AND.ITEM.EQ.215) UP4 = P4                                ; mapped 4->4


;For UPDRS - Item 216: Hand pronation  and supination  - left in M (#39, 3.6b) and Hand pronation  and supination  -
left in U (#216)
IF(MODEL.EQ.GR4.AND.ENDPT.EQ.2.AND.ITEM.EQ.216.AND.DV.EQ.0) P = P0                      ; mapped 0->0
IF(MODEL.EQ.GR4.AND.ENDPT.EQ.2.AND.ITEM.EQ.216.AND.DV.EQ.1) P = P1 + P2*FR2             ; mapped 1->1|2
IF(MODEL.EQ.GR4.AND.ENDPT.EQ.2.AND.ITEM.EQ.216.AND.DV.EQ.2) P = P2*(1-FR2) + P3*FR3     ; mapped 2->2|3
IF(MODEL.EQ.GR4.AND.ENDPT.EQ.2.AND.ITEM.EQ.216.AND.DV.EQ.3) P = P3*(1-FR3)              ; mapped 3->3
IF(MODEL.EQ.GR4.AND.ENDPT.EQ.2.AND.ITEM.EQ.216.AND.DV.EQ.4) P = P4                      ; mapped 4->4
IF(MODEL.EQ.GR4.AND.ENDPT.EQ.2.AND.ITEM.EQ.216) UP0 = P0                                ; mapped 0->0
IF(MODEL.EQ.GR4.AND.ENDPT.EQ.2.AND.ITEM.EQ.216) UP1 = P1 + P2*FR2                       ; mapped 1->1|2
IF(MODEL.EQ.GR4.AND.ENDPT.EQ.2.AND.ITEM.EQ.216) UP2 = P2*(1-FR2) + P3*FR3               ; mapped 2->2|3
IF(MODEL.EQ.GR4.AND.ENDPT.EQ.2.AND.ITEM.EQ.216) UP3 = P3*(1-FR3)                        ; mapped 3->3
IF(MODEL.EQ.GR4.AND.ENDPT.EQ.2.AND.ITEM.EQ.216) UP4 = P4                                ; mapped 4->4


;For UPDRS - Item 217: Hand pronation  and supination  - right in M (#38, 3.6a) and Hand pronation  and supination -
left in U (#217)
IF(MODEL.EQ.GR4.AND.ENDPT.EQ.2.AND.ITEM.EQ.217.AND.DV.EQ.0) P = P0                      ; mapped 0->0
IF(MODEL.EQ.GR4.AND.ENDPT.EQ.2.AND.ITEM.EQ.217.AND.DV.EQ.1) P = P1 + P2*FR2             ; mapped 1->1|2
IF(MODEL.EQ.GR4.AND.ENDPT.EQ.2.AND.ITEM.EQ.217.AND.DV.EQ.2) P = P2*(1-FR2) + P3*FR3     ; mapped 2->2|3
IF(MODEL.EQ.GR4.AND.ENDPT.EQ.2.AND.ITEM.EQ.217.AND.DV.EQ.3) P = P3*(1-FR3)              ; mapped 3->3
IF(MODEL.EQ.GR4.AND.ENDPT.EQ.2.AND.ITEM.EQ.217.AND.DV.EQ.4) P = P4                      ; mapped 4->4
IF(MODEL.EQ.GR4.AND.ENDPT.EQ.2.AND.ITEM.EQ.217) UP0 = P0                                ; mapped 0->0
IF(MODEL.EQ.GR4.AND.ENDPT.EQ.2.AND.ITEM.EQ.217) UP1 = P1 + P2*FR2                       ; mapped 1->1|2
IF(MODEL.EQ.GR4.AND.ENDPT.EQ.2.AND.ITEM.EQ.217) UP2 = P2*(1-FR2) + P3*FR3               ; mapped 2->2|3
IF(MODEL.EQ.GR4.AND.ENDPT.EQ.2.AND.ITEM.EQ.217) UP3 = P3*(1-FR3)                        ; mapped 3->3
IF(MODEL.EQ.GR4.AND.ENDPT.EQ.2.AND.ITEM.EQ.217) UP4 = P4                                ; mapped 4->4
```

```
;For UPDRS - Item 218: Leg agility - left in M (#43, 3.8b) and Leg agility - left in U (#218)
IF(MODEL.EQ.GR4.AND.ENDPT.EQ.2.AND.ITEM.EQ.218.AND.DV.EQ.0) P = P0                     ; mapped 0->0
IF(MODEL.EQ.GR4.AND.ENDPT.EQ.2.AND.ITEM.EQ.218.AND.DV.EQ.1) P = P1 + P2*FR2            ; mapped 1->1|2
IF(MODEL.EQ.GR4.AND.ENDPT.EQ.2.AND.ITEM.EQ.218.AND.DV.EQ.2) P = P2*(1-FR2) + P3*FR3    ; mapped 2->2|3
IF(MODEL.EQ.GR4.AND.ENDPT.EQ.2.AND.ITEM.EQ.218.AND.DV.EQ.3) P = P3*(1-FR3)             ; mapped 3->3
IF(MODEL.EQ.GR4.AND.ENDPT.EQ.2.AND.ITEM.EQ.218.AND.DV.EQ.4) P = P4                     ; mapped 4->4
IF(MODEL.EQ.GR4.AND.ENDPT.EQ.2.AND.ITEM.EQ.218) UP0 = P0                               ; mapped 0->0
IF(MODEL.EQ.GR4.AND.ENDPT.EQ.2.AND.ITEM.EQ.218) UP1 = P1 + P2*FR2                      ; mapped 1->1|2
IF(MODEL.EQ.GR4.AND.ENDPT.EQ.2.AND.ITEM.EQ.218) UP2 = P2*(1-FR2) + P3*FR3              ; mapped 2->2|3
IF(MODEL.EQ.GR4.AND.ENDPT.EQ.2.AND.ITEM.EQ.218) UP3 = P3*(1-FR3)                       ; mapped 3->3
IF(MODEL.EQ.GR4.AND.ENDPT.EQ.2.AND.ITEM.EQ.218) UP4 = P4                               ; mapped 4->4


;For UPDRS - Item 219: Leg agility - right in M (#42, 3.8a) and Leg agility - right in U (#219)
IF(MODEL.EQ.GR4.AND.ENDPT.EQ.2.AND.ITEM.EQ.219.AND.DV.EQ.0) P = P0                     ; mapped 0->0
IF(MODEL.EQ.GR4.AND.ENDPT.EQ.2.AND.ITEM.EQ.219.AND.DV.EQ.1) P = P1 + P2*FR2            ; mapped 1->1|2
IF(MODEL.EQ.GR4.AND.ENDPT.EQ.2.AND.ITEM.EQ.219.AND.DV.EQ.2) P = P2*(1-FR2) + P3*FR3    ; mapped 2->2|3
IF(MODEL.EQ.GR4.AND.ENDPT.EQ.2.AND.ITEM.EQ.219.AND.DV.EQ.3) P = P3*(1-FR3)             ; mapped 3->3
IF(MODEL.EQ.GR4.AND.ENDPT.EQ.2.AND.ITEM.EQ.219.AND.DV.EQ.4) P = P4                     ; mapped 4->4
IF(MODEL.EQ.GR4.AND.ENDPT.EQ.2.AND.ITEM.EQ.219) UP0 = P0                               ; mapped 0->0
IF(MODEL.EQ.GR4.AND.ENDPT.EQ.2.AND.ITEM.EQ.219) UP1 = P1 + P2*FR2                      ; mapped 1->1|2
IF(MODEL.EQ.GR4.AND.ENDPT.EQ.2.AND.ITEM.EQ.219) UP2 = P2*(1-FR2) + P3*FR3              ; mapped 2->2|3
IF(MODEL.EQ.GR4.AND.ENDPT.EQ.2.AND.ITEM.EQ.219) UP3 = P3*(1-FR3)                       ; mapped 3->3
IF(MODEL.EQ.GR4.AND.ENDPT.EQ.2.AND.ITEM.EQ.219) UP4 = P4                               ; mapped 4->4


; Item 27: Arising from chair in M (#44, 3.9) and Arising from chair in U (#27) respectively
IF(MODEL.EQ.GR4.AND.ENDPT.EQ.2.AND.ITEM.EQ.27.AND.DV.EQ.0) P = P0                      ; mapped 0->0
IF(MODEL.EQ.GR4.AND.ENDPT.EQ.2.AND.ITEM.EQ.27.AND.DV.EQ.1) P = P1                      ; mapped 1->1
IF(MODEL.EQ.GR4.AND.ENDPT.EQ.2.AND.ITEM.EQ.27.AND.DV.EQ.2) P = P2                      ; mapped 2->2
IF(MODEL.EQ.GR4.AND.ENDPT.EQ.2.AND.ITEM.EQ.27.AND.DV.EQ.3) P = P3                      ; mapped 3->3
IF(MODEL.EQ.GR4.AND.ENDPT.EQ.2.AND.ITEM.EQ.27.AND.DV.EQ.4) P = P4                      ; mapped 4->4
IF(MODEL.EQ.GR4.AND.ENDPT.EQ.2.AND.ITEM.EQ.27) UP0 = P0                                ; mapped 0->0
IF(MODEL.EQ.GR4.AND.ENDPT.EQ.2.AND.ITEM.EQ.27) UP1 = P1                                ; mapped 1->1
IF(MODEL.EQ.GR4.AND.ENDPT.EQ.2.AND.ITEM.EQ.27) UP2 = P2                                ; mapped 2->2
IF(MODEL.EQ.GR4.AND.ENDPT.EQ.2.AND.ITEM.EQ.27) UP3 = P3                                ; mapped 3->3
IF(MODEL.EQ.GR4.AND.ENDPT.EQ.2.AND.ITEM.EQ.27) UP4 = P4                                ; mapped 4->4


;For UPDRS -  Item 28: Posture in M (#48, 3.13) and Posture in U (#28)
IF(MODEL.EQ.GR4.AND.ENDPT.EQ.2.AND.ITEM.EQ.28.AND.DV.EQ.0) P = P0                      ; mapped 0->0
IF(MODEL.EQ.GR4.AND.ENDPT.EQ.2.AND.ITEM.EQ.28.AND.DV.EQ.1) P = P1                      ; mapped 1->1
IF(MODEL.EQ.GR4.AND.ENDPT.EQ.2.AND.ITEM.EQ.28.AND.DV.EQ.2) P = P2 + P3                 ; mapped 2->2|3
IF(MODEL.EQ.GR4.AND.ENDPT.EQ.2.AND.ITEM.EQ.28.AND.DV.EQ.3) P = P4*FR4                  ; mapped 3->4
IF(MODEL.EQ.GR4.AND.ENDPT.EQ.2.AND.ITEM.EQ.28.AND.DV.EQ.4) P = P4*(1-FR4)              ; mapped 4->4
IF(MODEL.EQ.GR4.AND.ENDPT.EQ.2.AND.ITEM.EQ.28) UP0 = P0                                ; mapped 0->0
IF(MODEL.EQ.GR4.AND.ENDPT.EQ.2.AND.ITEM.EQ.28) UP1 = P1                                ; mapped 1->1
IF(MODEL.EQ.GR4.AND.ENDPT.EQ.2.AND.ITEM.EQ.28) UP2 = P2 + P3                           ; mapped 2->2|3
IF(MODEL.EQ.GR4.AND.ENDPT.EQ.2.AND.ITEM.EQ.28) UP3 = P4*FR4                            ; mapped 3->4
IF(MODEL.EQ.GR4.AND.ENDPT.EQ.2.AND.ITEM.EQ.28) UP4 = P4*(1-FR4)                        ; mapped 4->4
```

```
;For UPDRS - Item 29: Gait in M (#45, 3.10) and Gait in U (#29)
IF(MODEL.EQ.GR4.AND.ENDPT.EQ.2.AND.ITEM.EQ.29.AND.DV.EQ.0) P = P0                    ; mapped 0->0
IF(MODEL.EQ.GR4.AND.ENDPT.EQ.2.AND.ITEM.EQ.29.AND.DV.EQ.1) P = P1                    ; mapped 1->1
IF(MODEL.EQ.GR4.AND.ENDPT.EQ.2.AND.ITEM.EQ.29.AND.DV.EQ.2) P = P2                    ; mapped 2->2
IF(MODEL.EQ.GR4.AND.ENDPT.EQ.2.AND.ITEM.EQ.29.AND.DV.EQ.3) P = P3 + P4*FR4           ; mapped 3->3|4
IF(MODEL.EQ.GR4.AND.ENDPT.EQ.2.AND.ITEM.EQ.29.AND.DV.EQ.4) P = P4*(1-FR4)            ; mapped 4->4
IF(MODEL.EQ.GR4.AND.ENDPT.EQ.2.AND.ITEM.EQ.29) UP0 = P0                              ; mapped 0->0
IF(MODEL.EQ.GR4.AND.ENDPT.EQ.2.AND.ITEM.EQ.29) UP1 = P1                              ; mapped 1->1
IF(MODEL.EQ.GR4.AND.ENDPT.EQ.2.AND.ITEM.EQ.29) UP2 = P2                              ; mapped 2->2
IF(MODEL.EQ.GR4.AND.ENDPT.EQ.2.AND.ITEM.EQ.29) UP3 = P3 + P4*FR4                     ; mapped 3->3|4
IF(MODEL.EQ.GR4.AND.ENDPT.EQ.2.AND.ITEM.EQ.29) UP4 = P4*(1-FR4)                      ; mapped 4->4


;For UPDRS - Item 30: Postural stability in M (#47, 3.12) and Postural stability in U (#30)
IF(MODEL.EQ.GR4.AND.ENDPT.EQ.2.AND.ITEM.EQ.30.AND.DV.EQ.0) P = P0                    ; mapped 0->0
IF(MODEL.EQ.GR4.AND.ENDPT.EQ.2.AND.ITEM.EQ.30.AND.DV.EQ.1) P = P1 + P2               ; mapped 1->1|2
IF(MODEL.EQ.GR4.AND.ENDPT.EQ.2.AND.ITEM.EQ.30.AND.DV.EQ.2) P = P3                    ; mapped 2->3
IF(MODEL.EQ.GR4.AND.ENDPT.EQ.2.AND.ITEM.EQ.30.AND.DV.EQ.3) P = P4*FR4                ; mapped 3->4
IF(MODEL.EQ.GR4.AND.ENDPT.EQ.2.AND.ITEM.EQ.30.AND.DV.EQ.4) P = P4*(1-FR4)            ; mapped 4->4
IF(MODEL.EQ.GR4.AND.ENDPT.EQ.2.AND.ITEM.EQ.30) UP0 = P0                              ; mapped 0->0
IF(MODEL.EQ.GR4.AND.ENDPT.EQ.2.AND.ITEM.EQ.30) UP1 = P1 + P2                         ; mapped 1->1|2
IF(MODEL.EQ.GR4.AND.ENDPT.EQ.2.AND.ITEM.EQ.30) UP2 = P3                              ; mapped 2->3
IF(MODEL.EQ.GR4.AND.ENDPT.EQ.2.AND.ITEM.EQ.30) UP3 = P4*FR4                          ; mapped 3->4
IF(MODEL.EQ.GR4.AND.ENDPT.EQ.2.AND.ITEM.EQ.30) UP4 = P4*(1-FR4)                      ; mapped 4->4


;For UPDRS - Item 31: Global spontaneity of movement  in M (#49, 3.14) and Body bradykinesia and hypokinesia in U
(#31)
IF(MODEL.EQ.GR4.AND.ENDPT.EQ.2.AND.ITEM.EQ.31.AND.DV.EQ.0) P = P0                    ; mapped 0->0
IF(MODEL.EQ.GR4.AND.ENDPT.EQ.2.AND.ITEM.EQ.31.AND.DV.EQ.1) P = P1                    ; mapped 1->1
IF(MODEL.EQ.GR4.AND.ENDPT.EQ.2.AND.ITEM.EQ.31.AND.DV.EQ.2) P = P2                    ; mapped 2->2
IF(MODEL.EQ.GR4.AND.ENDPT.EQ.2.AND.ITEM.EQ.31.AND.DV.EQ.3) P = P3                    ; mapped 3->3
IF(MODEL.EQ.GR4.AND.ENDPT.EQ.2.AND.ITEM.EQ.31.AND.DV.EQ.4) P = P4                    ; mapped 4->4
IF(MODEL.EQ.GR4.AND.ENDPT.EQ.2.AND.ITEM.EQ.31) UP0 = P0                              ; mapped 0->0
IF(MODEL.EQ.GR4.AND.ENDPT.EQ.2.AND.ITEM.EQ.31) UP1 = P1                              ; mapped 1->1
IF(MODEL.EQ.GR4.AND.ENDPT.EQ.2.AND.ITEM.EQ.31) UP2 = P2                              ; mapped 2->2
IF(MODEL.EQ.GR4.AND.ENDPT.EQ.2.AND.ITEM.EQ.31) UP3 = P3                              ; mapped 3->3
IF(MODEL.EQ.GR4.AND.ENDPT.EQ.2.AND.ITEM.EQ.31) UP4 = P4                              ; mapped 4->4

;-----------------------------------------------------------------------------
IPRED = (UP1*1)+(UP2*2)+(UP3*3)+(UP4*4)
RES = DV - IPRED

;------------------------Response probability prediction-------------------------
IF(P.LT.1E-16)     P = 1E-16     ; protection for P->0
IF(P.GT.(1-1E-16)) P = 1-1E-16   ; protection for P->1

Y = -2*LOG(P)
```

```
;--------------------------------Simulation code--------------------------------
UPGE1=1-UP0
UPGE2=UPGE1 - UP1
UPGE3=UPGE2 - UP2
UPGE4=UPGE3 - UP3

IF(ICALL.EQ.4) THEN

IF(MODEL.EQ.GR4) THEN
CALL RANDOM (2,R)

SDV=0
IF(R.LT.UPGE1) SDV=1
IF(R.LT.UPGE2) SDV=2
IF(R.LT.UPGE3) SDV=3
IF(R.LT.UPGE4) SDV=4
ENDIF

DV=SDV

ENDIF
;---------------------------------------------------------------------------------

NID = IREP    ; Required for simulation

$MIX
NSPOP=2

P(1)=THETA(267)        ;basic assumption that left & right side
P(2)=1-P(1)            ;problems are equally common.  P(1)=0.5 FIX

;Sim_start for VPC
$ESTIMATION MAXEVAL=999999  METHOD=COND LAPLACE -2LL PRINT=1 NOABORTMSFO=msf_run140

;$SIMULATION (21398012) (32131288 UNI) ONLYSIMULATION NOPREDICTION NSUB=200 ; for VPC
;Sim_end for VPC


$TABLE     ID ITEM DV PSI ETA1 ETA2 ETA3 ETA4 TIME IPRED RESFILE=psi_estimates_tab140 NOAPPEND
ONEHEADER NOPRINT
$TABLE     ITEM DIS DIF1 DIF2 DIF3 DIF4 FILE=item_parameters_tab140NOAPPEND  ONEHEADER NOPRINT

$THETA  0.803713 FIX ; 1 I1DISGR4
$THETA  1.40615 FIX ; 2         I1DIF1GR4
$THETA  2.83611 FIX ; 3 I1DIF2GR4
$THETA  2.43466 FIX ; 4         I1DIF3GR4
$THETA  50 FIX ; 5       I1DIF4GR4
$THETA  (0,0.489059,1) FIX ; 6 I1 - FR1
$THETA  1 FIX ; 7 I1 - FR4
$THETA  0.71797 FIX ; 8         I2DISGR4
$THETA  4.44303 FIX ; 9         I2DIF1GR4
$THETA  3.88909 FIX ; 10 I2DIF2GR4
$THETA  50 FIX ; 11 I2DIF3GR4
$THETA  50 FIX ; 12 I2DIF4GR4
```

```
$THETA  (0,0.839851,1) FIX ; 13 I2 - FR0
$THETA  0.761187 FIX ; 14 I3DISGR4
$THETA  1.60376 FIX ; 15 I3DIF1GR4
$THETA  2.44851 FIX ; 16 I3DIF2GR4
$THETA  2.18606 FIX ; 17 I3DIF3GR4
$THETA  50 FIX ; 18 I3DIF4GR4
$THETA  1.06393 FIX ; 19 I4DISGR4
$THETA  1.65482 FIX ; 20 I4DIF1GR4
$THETA  1.63278 FIX ; 21 I4DIF2GR4
$THETA  2.2184 FIX ; 22 I4DIF3GR4
$THETA  50 FIX ; 23 I4DIF4GR4
$THETA  1.10263 FIX ; 24 I5DISGR4
$THETA  0.654278 FIX ; 25 I5DIF1GR4
$THETA  1.39602 FIX ; 26 I5DIF2GR4
$THETA  2.07094 FIX ; 27 I5DIF3GR4
$THETA  50 FIX ; 28 I5DIF4GR4
$THETA  0.996294 FIX ; 29 I6DISGR4
$THETA  0.704732 FIX ; 30 I6DIF1GR4
$THETA  0.972972 FIX ; 31 I6DIF2GR4
$THETA  1.29208 FIX ; 32 I6DIF3GR4
$THETA  2.27295 FIX ; 33 I6DIF4GR4
$THETA  1 FIX ; 34 I6 - FR1
$THETA  (0,0.763157,1) FIX ; 35 I6 - FR3
$THETA  1.03404 FIX ; 36 I7DISGR4
$THETA  1.87628 FIX ; 37 I7DIF1GR4
$THETA  2.90417 FIX ; 38 I7DIF2GR4
$THETA  0.791996 FIX ; 39 I7DIF3GR4
$THETA  50 FIX ; 40 I7DIF4GR4
$THETA  (0,0.570707,1) FIX ; 41 I7 - FR1
$THETA  0 FIX ; 42 I7 - FR3
$THETA  0.897659 FIX ; 43 I8DISGR4
$THETA  -0.653523 FIX ; 44 I8DIF1GR4
$THETA  2.11213 FIX ; 45 I8DIF2GR4
$THETA  1.93243 FIX ; 46 I8DIF3GR4
$THETA  2.26932 FIX ; 47 I8DIF4GR4
$THETA  (0,0.637975,1) FIX ; 48 I8 - FR1
$THETA  1.29642 FIX ; 49 I9DISGR4
$THETA  0.627478 FIX ; 50 I9DIF1GR4
$THETA  2.1697 FIX ; 51          I9DIF2GR4
$THETA  3.68103 FIX ; 52 I9DIF3GR4
$THETA  (0,2.05871,50) FIX ; 53          I9DIF4GR4
$THETA  1.6966 FIX ; 54 I10DISGR4
$THETA  0.248416 FIX ; 55 I10DIF1GR4
$THETA  1.9276 FIX ; 56          I10DIF2GR4
$THETA  2.55871 FIX ; 57 I10DIF3GR4
$THETA  (0,1.82693,50) FIX ; 58 I10DIF4GR4
$THETA  1.32065 FIX ; 59 I11DISGR4
$THETA  0.883551 FIX ; 60 I11DIF1GR4
$THETA  3.61457 FIX ; 61 I11DIF2GR4
$THETA  (0,1.40174,50) FIX ; 62          I11DIF3GR4
$THETA  50 FIX ; 63     I11DIF4GR4
$THETA  1.49996 FIX ; 64 I12DISGR4
$THETA  0.782463 FIX ; 65 I12DIF1GR4
$THETA  2.5981 FIX ; 66          I12DIF2GR4
$THETA  2.2005 FIX ; 67          I12DIF3GR4
```

$THETA (0,1.89617,50) FIX ; 68   I12DIF4GR4
$THETA 0 FIX ; 69 I12 - FR3
$THETA (0,4.32206E-05,1) FIX ; 70 I12 - FR4
$THETA (0,1.2563) FIX ; 71   I13DISGR4
$THETA 2.53524 FIX ; 72 I13DIF1GR4
$THETA (0,1.61318,50) FIX ; 73 I13DIF2GR4
THETA (0,1.72808,50) FIX ; 74 I13DIF3GR4
$THETA 50 FIX ; 75 I13DIF4GR4
$THETA 1.53813 FIX ; 76 I14DISGR4
$THETA 2.39558 FIX ; 77 I14DIF1GR4
$THETA 1.40572 FIX ; 78 I14DIF2GR4
$THETA 1.52182 FIX ; 79 I14DIF3GR4
$THETA (0,1.8087,50) FIX ; 80 I14DIF4GR4
$THETA 1.44877 FIX ; 81 I15DISGR4
$THETA 0.38194 FIX ; 82 I15DIF1GR4
$THETA 2.60576 FIX ; 83 I15DIF2GR4
$THETA 0.879622 FIX ; 84 I15DIF3GR4
$THETA 50 FIX ; 85 I15DIF4GR4
$THETA (0,0.93929,1) FIX ; 86 I15 - FR1
$THETA 1 FIX ; 87 I15 - FR4
$THETA 0.288752 FIX ; 88 I16DISGR4
$THETA -6.21885 FIX ; 89 I16DIF1GR4
$THETA 9.909 FIX ; 90 I16DIF2GR4
$THETA 7.69428 FIX ; 91 I16DIF3GR4
$THETA 7.86785 FIX ; 92 I16DIF4GR4
$THETA 0.846703 FIX ; 93 I17DISGR4
$THETA -0.11323 FIX ; 94 I17DIF1GR4
$THETA 2.55438 FIX ; 95 I17DIF2GR4
$THETA 1.46382 FIX ; 96 I17DIF3GR4
$THETA 50 FIX ; 97 I17DIF4GR4
$THETA 1.13648 FIX ; 98 I18DISGR4
$THETA 0.0399426 FIX ; 99 I18DIF1GR4
$THETA 2.93027 FIX ; 100 I18DIF2GR4
$THETA 3.0166 FIX ; 101 I18DIF3GR4
$THETA (0,2.90237,50) FIX ; 102 I18DIF4GR4
$THETA 0 FIX ; 103 I18 - FR4
$THETA 1.56729 FIX ; 104 I19DISGR4
$THETA -1.57629 FIX ; 105 I19DIF1GR4
$THETA 2.41614 FIX ; 106 I19DIF2GR4
$THETA 2.16117 FIX ; 107 I19DIF3GR4
$THETA 1.92412 FIX ; 108 I19DIF4GR4
$THETA (0,0.41994) FIX ; 109 I200DISGR4
$THETA 5.23962 FIX ; 110 I200DIF1GR4
$THETA (0,4.51487,50) FIX ; 111 I200DIF2GR4
$THETA (0,7.00492,50) FIX ; 112 I200DIF3GR4
$THETA 50 FIX ; 113 I200DIF4GR4
$THETA (0,0.722511) FIX ; 114 I201DISGR4
$THETA -0.615796 FIX ; 115 I201DIF1GR4
$THETA (0,2.08185,50) FIX ; 116 I201DIF2GR4
$THETA (0,2.56527,50) FIX ; 117 I201DIF3GR4
$THETA (0,5.21145,50) FIX ; 118 I201DIF4GR4
$THETA (0,0.76232) FIX ; 119 I202DISGR4
$THETA -0.490957 FIX ; 120 I202DIF1GR4
$THETA (0,1.86388,50) FIX ; 121 I202DIF2GR4
$THETA (0,2.3566,50) FIX ; 122 I202DIF3GR4

$THETA (0,3.81822,50) FIX ; 123 I202DIF4GR4
$THETA (0,0.801371) FIX ; 124 I203DISGR4
$THETA 1.24697 FIX ; 125 I203DIF1GR4
$THETA (0,2.13524,50) FIX ; 126 I203DIF2GR4
$THETA (0,2.80687,50) FIX ; 127 I203DIF3GR4
$THETA 50 FIX ; 128 I203DIF4GR4
$THETA (0,0.90363) FIX ; 129 I204DISGR4
$THETA 1.14212 FIX ; 130 I204DIF1GR4
$THETA (0,2.00227,50) FIX ; 131 I204DIF2GR4
$THETA (0,2.65596,50) FIX ; 132 I204DIF3GR4
$THETA 50 FIX ; 133 I204DIF4GR4
$THETA (0,0.768156) FIX ; 134 I205DISGR4
$THETA 0.122414 FIX ; 135 I205DIF1GR4
$THETA (0,2.74,50) FIX ; 136 I205DIF2GR4
$THETA (0,2.55797,50) FIX ; 137 I205DIF3GR4
$THETA 50 FIX ; 138 I205DIF4GR4
$THETA (0,0.838694) FIX ; 139 I206DISGR4
$THETA 0.313319 FIX ; 140 I206DIF1GR4
$THETA (0,2.50512,50) FIX ; 141 I206DIF2GR4
$THETA (0,2.07038,50) FIX ; 142 I206DIF3GR4
$THETA (0,2.62577,50) FIX ; 143 I206DIF4GR4
$THETA 1.0188 FIX ; 144 I207DISGR4
$THETA -0.023059 FIX ; 145 I207DIF1GR4
$THETA 1.6141 FIX ; 146 I207DIF2GR4
$THETA 3.01527 FIX ; 147 I207DIF3GR4
$THETA 3.27526 FIX ; 148 I207DIF4GR4
$THETA (0,0.839793,1) FIX ; 149 I207 - FR2
$THETA 1 FIX ; 150 I207 - FR4
$THETA 1.22586 FIX ; 151 I208DISGR4
$THETA -1.96464 FIX ; 152 I208DIF1GR4
$THETA 1.82279 FIX ; 153 I208DIF2GR4
$THETA 2.74521 FIX ; 154 I208DIF3GR4
$THETA (0,8.80468E-06,50) FIX ; 155 I208DIF4GR4
$THETA 1 FIX ; 156 I208 - FR2
$THETA (0,0.953493,1) FIX ; 157 I208 - FR4
$THETA 1.20822 FIX ; 158 I209DISGR4
$THETA -2.12428 FIX ; 159 I209DIF1GR4
$THETA 1.90608 FIX ; 160 I209DIF2GR4
$THETA 3.05783 FIX ; 161 I209DIF3GR4
$THETA 3.23746 FIX ; 162 I209DIF4GR4
$THETA (0,0.815199,1) FIX ; 163 I209 - FR2
$THETA 1 FIX ; 164 I209 - FR4
$THETA 0.928848 FIX ; 165 I210DISGR4
$THETA -0.728983 FIX ; 166 I210DIF1GR4
$THETA 1.65765 FIX ; 167 I210DIF2GR4
$THETA 2.712 FIX ; 168 I210DIF3GR4
$THETA (0,4.51252E-07,50) FIX ; 169 I210DIF4GR4
$THETA 1 FIX ; 170 I210 - FR2
$THETA (0,0.870967,1) FIX ; 171 I210 - FR4
$THETA 0.847223 FIX ; 172 I211DISGR4
$THETA -0.700967 FIX ; 173 I211DIF1GR4
$THETA 1.93385 FIX ; 174 I211DIF2GR4
$THETA 3.13601 FIX ; 175 I211DIF3GR4
$THETA 5.01593 FIX ; 176 I211DIF4GR4
$THETA (0,0.789817,1) FIX ; 177 I211 - FR2

$THETA 1 FIX ; 178 I211 - FR4
$THETA 1.77344 FIX ; 179 I212DISGR4
$THETA -2.11306 FIX ; 180 I212DIF1GR4
$THETA 1.72031 FIX ; 181 I212DIF2GR4
$THETA 1.64843 FIX ; 182 I212DIF3GR4
$THETA 2.03188 FIX ; 183 I212DIF4GR4
$THETA (0,0.204069,1) FIX ; 184 I212 - FR2
$THETA (0,0.45494,1) FIX ; 185 I212 - FR3
$THETA 1.78511 FIX ; 186 I213DISGR4
$THETA -1.82053 FIX ; 187 I213DIF1GR4
$THETA 1.83348 FIX ; 188 I213DIF2GR4
$THETA 1.55998 FIX ; 189 I213DIF3GR4
$THETA 2.06541 FIX ; 190 I213DIF4GR4
$THETA (0,0.0996191,1) FIX ; 191 I213 - FR2
$THETA (0,0.412768,1) FIX ; 192 I213 - FR3
$THETA 1.74286 FIX ; 193 I214DISGR4
$THETA -1.74416 FIX ; 194 I214DIF1GR4
$THETA 1.71019 FIX ; 195 I214DIF2GR4
$THETA 1.69005 FIX ; 196 I214DIF3GR4
$THETA 50 FIX ; 197 I214DIF4GR4
$THETA (0,0.232096,1) FIX ; 198 I214 - FR2
$THETA (0,0.523686,1) FIX ; 199 I214 - FR3
$THETA 1.8687 FIX ; 200 I215DISGR4
$THETA -1.27143 FIX ; 201 I215DIF1GR4
$THETA 1.71903 FIX ; 202 I215DIF2GR4
$THETA 1.50844 FIX ; 203 I215DIF3GR4
$THETA 50 FIX ; 204 I215DIF4GR4
$THETA (0,0.0660135,1) FIX ; 205 I215 - FR2
$THETA (0,0.554531,1) FIX ; 206 I215 - FR3
$THETA 1.74941 FIX ; 207 I216DISGR4
$THETA -1.55253 FIX ; 208 I216DIF1GR4
$THETA 1.60473 FIX ; 209 I216DIF2GR4
$THETA 1.5204 FIX ; 210 I216DIF3GR4
$THETA 1.94249 FIX ; 211 I216DIF4GR4
$THETA (0,0.228738,1) FIX ; 212 I216 - FR2
$THETA (0,0.440191,1) FIX ; 213 I216 - FR3
$THETA 1.56146 FIX ; 214 I217DISGR4
$THETA -1.30857 FIX ; 215 I217DIF1GR4
$THETA 1.84053 FIX ; 216 I217DIF2GR4
$THETA 1.83978 FIX ; 217 I217DIF3GR4
$THETA 2.09752 FIX ; 218 I217DIF4GR4
$THETA (0,0.085318,1) FIX ; 219 I217 - FR2
$THETA (0,0.281307,1) FIX ; 220 I217 - FR3
$THETA 1.2669 FIX ; 221 I218DISGR4
$THETA -0.965298 FIX ; 222 I218DIF1GR4
$THETA 1.88115 FIX ; 223 I218DIF2GR4
$THETA 2.07974 FIX ; 224 I218DIF3GR4
$THETA 3.37931 FIX ; 225 I218DIF4GR4
$THETA (0,3.40241E-05,1) FIX ; 226 I218 - FR2
$THETA (0,4.56409E-05,1) FIX ; 227 I218 - FR3
$THETA 1.01247 FIX ; 228 I219DISGR4
$THETA -0.432787 FIX ; 229 I219DIF1GR4
$THETA 2.50412 FIX ; 230 I219DIF2GR4
$THETA 2.41378 FIX ; 231 I219DIF3GR4
$THETA (0,2.09224,50) FIX ; 232 I219DIF4GR4

```
$THETA  0 FIX ; 233 I219 - FR2
$THETA  0 FIX ; 234 I219 - FR3
$THETA  1.12653 FIX ; 235 I27DISGR4
$THETA  1.87872 FIX ; 236 I27DIF1GR4
$THETA  2.01436 FIX ; 237 I27DIF2GR4
$THETA  1.39345 FIX ; 238 I27DIF3GR4
$THETA  (0,1.45273,50) FIX ; 239 I27DIF4GR4
$THETA  1.2741 FIX ; 240        I28DISGR4
$THETA  -0.267655 FIX ; 241  I28DIF1GR4
$THETA  2.3296 FIX ; 242        I28DIF2GR4
$THETA  2.183 FIX ; 243          I28DIF3GR4
$THETA  2.38394 FIX ; 244      I28DIF4GR4
$THETA  (0,0.916666,1) FIX ; 245 I28 - FR4
$THETA  0.921066 FIX ; 246 I29DISGR4
$THETA  -0.530405 FIX ; 247  I29DIF1GR4
$THETA  3.9975 FIX ; 248 I29DIF2GR4
$THETA  2.53708 FIX ; 249 I29DIF3GR4
$THETA  (0,2.35565,50) FIX ; 250 I29DIF4GR4
$THETA  0 FIX ; 251 I29 - FR4
$THETA  0.796367 FIX ; 252 I30DISGR4
$THETA  3.21117 FIX ; 253 I30DIF1GR4
$THETA  1.6323 FIX ; 254 I30DIF2GR4
$THETA  1.00485 FIX ; 255 I30DIF3GR4
$THETA  (0,3.00596,50) FIX ; 256 I30DIF4GR4
$THETA  (0,0.928572,1) FIX ; 257 I30 - FR4
$THETA  1.81744 FIX ; 258 I31DISGR4
$THETA  -1.47475 FIX ; 259 I31DIF1GR4
$THETA  1.88148 FIX ; 260 I31DIF2GR4
$THETA  1.81987 FIX ; 261 I31DIF3GR4
$THETA  3.58311 FIX ; 262 I31DIF4GR4
$THETA  0.528897 ; 263 PRS - PSI mean
$THETA  0.212961 ; 264 NDRS - PSI mean
$THETA  0.348838 ; 265 DRS - PSI mean
$THETA  (0,1.92962) ; 266 decrease in psi for being the unaffected side
$THETA  (0,0.549149,1) ; 267 Mixture prop
$THETA  0.694272 ; 268 PRS - shift in PRS for 169
$THETA  1.36502 ; 269 NDRS - shift in NDRS for 169
$THETA  0.313683 ; 270 DRS - shift in DRS for 169: for the same combination of affected and evaluated sides
$THETA  1.17354 ; 271 DRS - shift in DRS for 169: for the other combination of affected and evaluated sides
;$OMEGA  0.807078  ; 1 - Variance in PRS PSI - 168 (263)
;$OMEGA  1.86032  ; 2 - Variance in PRS PSI - 169
;$OMEGA  1.26155  ; 3 - Variance in NDRS PSI - 168 (264)
;$OMEGA  2.59959  ; 4 - Variance in NDRS 169
;$OMEGA  0.811566  ; 5 - Variance in DRS PSI - 168 (265)
;$OMEGA  1.48326  ; 6 - Variance in DRS PSI - 169
;$OMEGA  0.133559  ; 7 - Variance in decrease in psi for being the unaffected side (266)

$OMEGA  BLOCK(3)
 0.81  ; PRS for 168
 0.5 1.26  ; NDRS for 168
 0.62 0.5 0.81  ; DRS for 168

$OMEGA  BLOCK(3)
 1.86  ; PRS for 169
 0.23 2.6  ; NDRS for 169
```

```
 0.3 0.26 1.48  ; DRS for 169

$OMEGA  0.133559  ; 7 - Variance in decrease in psi for being the unaffected side (266)
```

STID	SUBJ	ITEM	DV	TIMEW	PRD	PDOS	AGE	WT	HT	ETHNICITY	MALE	CMSE	CMAM	CMAC	CMDP	FORM	TSDW	EXCL	ENDPTCAT	SEQ	TMT	DROPOUT	RDO	VISITW	VISITD	TDOW	ITEMM	PBO
169	4656	1	-99	0	1	0	49	102.5	188	1	1	0	0	0	1	0	140	0	4	0	0	0	0	0	0	26	0	1
169	4656	1	0	0	1	0	49	102.5	188	1	1	0	0	0	1	0	140	0	1	0	0	0	0	0	0	26	0	1
169	4656	1	1	0	1	0	49	102.5	188	1	1	0	0	0	1	0	140	0	3	0	0	0	0	0	0	26	0	1
169	4656	2	3	0	1	0	49	102.5	188	1	1	0	0	0	1	0	140	0	4	0	0	0	0	0	0	26	0	1
169	4656	2	0	0	1	0	49	102.5	188	1	1	0	0	0	1	0	140	0	3	0	0	0	0	0	0	26	0	1
169	4656	2	0	0	1	0	49	102.5	188	1	1	0	0	0	1	0	140	0	1	0	0	0	0	0	0	26	0	1
169	4656	3	0	0	1	0	49	102.5	188	1	1	0	0	0	1	0	140	0	3	0	0	0	0	0	0	26	0	1
169	4656	3	0	0	1	0	49	102.5	188	1	1	0	0	0	1	0	140	0	1	0	0	0	0	0	0	26	0	1
169	4656	4	0	0	1	0	49	102.5	188	1	1	0	0	0	1	0	140	0	3	0	0	0	0	0	0	26	0	1
169	4656	4	2	0	1	0	49	102.5	188	1	1	0	0	0	1	0	140	0	1	0	0	0	0	0	0	26	0	1
169	4656	5	0	0	1	0	49	102.5	188	1	1	0	0	0	1	0	140	0	3	0	0	0	0	0	0	26	0	1
169	4656	5	0	0	1	0	49	102.5	188	1	1	0	0	0	1	0	140	0	1	0	0	0	0	0	0	26	0	1
169	4656	6	0	0	1	0	49	102.5	188	1	1	0	0	0	1	0	140	0	3	0	0	0	0	0	0	26	0	1
169	4656	6	0	0	1	0	49	102.5	188	1	1	0	0	0	1	0	140	0	1	0	0	0	0	0	0	26	0	1
169	4656	7	0	0	1	0	49	102.5	188	1	1	0	0	0	1	0	140	0	3	0	0	0	0	0	0	26	0	1
169	4656	7	0	0	1	0	49	102.5	188	1	1	0	0	0	1	0	140	0	1	0	0	0	0	0	0	26	0	1
169	4656	8	0	0	1	0	49	102.5	188	1	1	0	0	0	1	0	140	0	3	0	0	0	0	0	0	26	0	1
169	4656	8	0	0	1	0	49	102.5	188	1	1	0	0	0	1	0	140	0	1	0	0	0	0	0	0	26	0	1
169	4656	9	0	0	1	0	49	102.5	188	1	1	0	0	0	1	0	140	0	3	0	0	0	0	0	0	26	0	1
169	4656	9	0	0	1	0	49	102.5	188	1	1	0	0	0	1	0	140	0	1	0	0	0	0	0	0	26	0	1
169	4656	10	0	0	1	0	49	102.5	188	1	1	0	0	0	1	0	140	0	1	0	0	0	0	0	0	26	0	1
169	4656	10	0	0	1	0	49	102.5	188	1	1	0	0	0	1	0	140	0	3	0	0	0	0	0	0	26	0	1
169	4656	11	0	0	1	0	49	102.5	188	1	1	0	0	0	1	0	140	0	1	0	0	0	0	0	0	26	0	1
169	4656	11	0	0	1	0	49	102.5	188	1	1	0	0	0	1	0	140	0	3	0	0	0	0	0	0	26	0	1
169	4656	12	0	0	1	0	49	102.5	188	1	1	0	0	0	1	0	140	0	1	0	0	0	0	0	0	26	0	1
169	4656	12	0	0	1	0	49	102.5	188	1	1	0	0	0	1	0	140	0	3	0	0	0	0	0	0	26	0	1
169	4656	13	0	0	1	0	49	102.5	188	1	1	0	0	0	1	0	140	0	1	0	0	0	0	0	0	26	0	1
169	4656	13	1	0	1	0	49	102.5	188	1	1	0	0	0	1	0	140	0	3	0	0	0	0	0	0	26	0	1
169	4656	14	0	0	1	0	49	102.5	188	1	1	0	0	0	1	0	140	0	1	0	0	0	0	0	0	26	0	1
169	4656	14	0	0	1	0	49	102.5	188	1	1	0	0	0	1	0	140	0	3	0	0	0	0	0	0	26	0	1
169	4656	15	0	0	1	0	49	102.5	188	1	1	0	0	0	1	0	140	0	1	0	0	0	0	0	0	26	0	1
169	4656	15	0	0	1	0	49	102.5	188	1	1	0	0	0	1	0	140	0	3	0	0	0	0	0	0	26	0	1
169	4656	16	1	0	1	0	49	102.5	188	1	1	0	0	0	1	0	140	0	3	0	0	0	0	0	0	26	0	1
169	4656	16	0	0	1	0	49	102.5	188	1	1	0	0	0	1	0	140	0	1	0	0	0	0	0	0	26	0	1
169	4656	17	0	0	1	0	49	102.5	188	1	1	0	0	0	1	0	140	0	3	0	0	0	0	0	0	26	0	1
169	4656	17	0	0	1	0	49	102.5	188	1	1	0	0	0	1	0	140	0	1	0	0	0	0	0	0	26	0	1

169	4656	18	0	0	1	0	49	102.5	188	1	1	0	0	0	1	0	140	0	3	0	0	0	0	0	0	26	0	1
169	4656	18	1	0	1	0	49	102.5	188	1	1	0	0	0	1	0	140	0	1	0	0	0	0	0	0	26	0	1
169	4656	19	0	0	1	0	49	102.5	188	1	1	0	0	0	1	0	140	0	3	0	0	0	0	0	0	26	0	1
169	4656	19	1	0	1	0	49	102.5	188	1	1	0	0	0	1	0	140	0	1	0	0	0	0	0	0	26	0	1
169	4656	20	1	0	1	0	49	102.5	188	1	1	0	0	0	1	0	140	0	3	0	0	0	0	0	0	26	0	1
169	4656	21	0	0	1	0	49	102.5	188	1	1	0	0	0	1	0	140	0	3	0	0	0	0	0	0	26	0	1
169	4656	22	2	0	1	0	49	102.5	188	1	1	0	0	0	1	0	140	0	3	0	0	0	0	0	0	26	0	1
169	4656	23	3	0	1	0	49	102.5	188	1	1	0	0	0	1	0	140	0	3	0	0	0	0	0	0	26	0	1
169	4656	24	2	0	1	0	49	102.5	188	1	1	0	0	0	1	0	140	0	3	0	0	0	0	0	0	26	0	1
169	4656	25	3	0	1	0	49	102.5	188	1	1	0	0	0	1	0	140	0	3	0	0	0	0	0	0	26	0	1
169	4656	26	2	0	1	0	49	102.5	188	1	1	0	0	0	1	0	140	0	3	0	0	0	0	0	0	26	0	1
169	4656	27	1	0	1	0	49	102.5	188	1	1	0	0	0	1	0	140	0	3	0	0	0	0	0	0	26	0	1
169	4656	27	0	0	1	0	49	102.5	188	1	1	0	0	0	1	0	140	0	1	0	0	0	0	0	0	26	0	1
169	4656	28	3	0	1	0	49	102.5	188	1	1	0	0	0	1	0	140	0	3	0	0	0	0	0	0	26	0	1
169	4656	28	1	0	1	0	49	102.5	188	1	1	0	0	0	1	0	140	0	1	0	0	0	0	0	0	26	0	1
169	4656	29	1	0	1	0	49	102.5	188	1	1	0	0	0	1	0	140	0	3	0	0	0	0	0	0	26	0	1
169	4656	29	0	0	1	0	49	102.5	188	1	1	0	0	0	1	0	140	0	1	0	0	0	0	0	0	26	0	1
169	4656	30	0	0	1	0	49	102.5	188	1	1	0	0	0	1	0	140	0	3	0	0	0	0	0	0	26	0	1
169	4656	30	1	0	1	0	49	102.5	188	1	1	0	0	0	1	0	140	0	1	0	0	0	0	0	0	26	0	1
169	4656	31	0	0	1	0	49	102.5	188	1	1	0	0	0	1	0	140	0	3	0	0	0	0	0	0	26	0	1
169	4656	31	1	0	1	0	49	102.5	188	1	1	0	0	0	1	0	140	0	1	0	0	0	0	0	0	26	0	1
169	4656	32	0	0	1	0	49	102.5	188	1	1	0	0	0	1	0	140	0	3	0	0	0	0	0	0	26	0	1
169	4656	32	0	0	1	0	49	102.5	188	1	1	0	0	0	1	0	140	0	1	0	0	0	0	0	0	26	0	1
169	4656	33	1	0	1	0	49	102.5	188	1	1	0	0	0	1	0	140	0	3	0	0	0	0	0	0	26	0	1
169	4656	33	0	0	1	0	49	102.5	188	1	1	0	0	0	1	0	140	0	1	0	0	0	0	0	0	26	0	1
169	4656	34	0	0	1	0	49	102.5	188	1	1	0	0	0	1	0	140	0	3	0	0	0	0	0	0	26	0	1
169	4656	34	0	0	1	0	49	102.5	188	1	1	0	0	0	1	0	140	0	1	0	0	0	0	0	0	26	0	1
169	4656	35	0	0	1	0	49	102.5	188	1	1	0	0	0	1	0	140	0	3	0	0	0	0	0	0	26	0	1
169	4656	35	0	0	1	0	49	102.5	188	1	1	0	0	0	1	0	140	0	1	0	0	0	0	0	0	26	0	1
169	4656	36	0	0	1	0	49	102.5	188	1	1	0	0	0	1	0	140	0	3	0	0	0	0	0	0	26	0	1
169	4656	36	1	0	1	0	49	102.5	188	1	1	0	0	0	1	0	140	0	1	0	0	0	0	0	0	26	0	1
169	4656	37	1	0	1	0	49	102.5	188	1	1	0	0	0	1	0	140	0	3	0	0	0	0	0	0	26	0	1
169	4656	37	0	0	1	0	49	102.5	188	1	1	0	0	0	1	0	140	0	1	0	0	0	0	0	0	26	0	1
169	4656	38	1	0	1	0	49	102.5	188	1	1	0	0	0	1	0	140	0	3	0	0	0	0	0	0	26	0	1
169	4656	38	0	0	1	0	49	102.5	188	1	1	0	0	0	1	0	140	0	1	0	0	0	0	0	0	26	0	1
169	4656	39	2	0	1	0	49	102.5	188	1	1	0	0	0	1	0	140	0	1	0	0	0	0	0	0	26	0	1
169	4656	40	0	0	1	0	49	102.5	188	1	1	0	0	0	1	0	140	0	1	0	0	0	0	0	0	26	0	1

169	4656	41	0	0	1	0	49	102.5	188	1	1	0	0	0	1	0	140	0	1	0	0	0	0	0	0	26	0	1
169	4656	42	0	0	1	0	49	102.5	188	1	1	0	0	0	1	0	140	0	1	0	0	0	0	0	0	26	0	1
169	4656	43	1.5	0	1	0	49	102.5	188	1	1	0	0	0	1	0	140	0	1	0	0	0	0	0	0	26	0	1
169	4656	44	100	0	1	0	49	102.5	188	1	1	0	0	0	1	0	140	0	1	0	0	0	0	0	0	26	0	1
169	4656	50	20	0	1	0	49	102.5	188	1	1	0	0	0	1	0	140	0	1	0	0	0	0	0	0	26	0	1
169	4656	200	0	0	1	0	49	102.5	188	1	1	0	0	0	1	0	140	0	1	0	0	0	0	0	0	26	0	1
169	4656	201	3	0	1	0	49	102.5	188	1	1	0	0	0	1	0	140	0	1	0	0	0	0	0	0	26	0	1
169	4656	202	0	0	1	0	49	102.5	188	1	1	0	0	0	1	0	140	0	1	0	0	0	0	0	0	26	0	1
169	4656	203	0	0	1	0	49	102.5	188	1	1	0	0	0	1	0	140	0	1	0	0	0	0	0	0	26	0	1
169	4656	204	0	0	1	0	49	102.5	188	1	1	0	0	0	1	0	140	0	1	0	0	0	0	0	0	26	0	1
169	4656	205	1	0	1	0	49	102.5	188	1	1	0	0	0	1	0	140	0	1	0	0	0	0	0	0	26	0	1
169	4656	206	0	0	1	0	49	102.5	188	1	1	0	0	0	1	0	140	0	1	0	0	0	0	0	0	26	0	1
169	4656	207	0	0	1	0	49	102.5	188	1	1	0	0	0	1	0	140	0	1	0	0	0	0	0	0	26	0	1
169	4656	208	2	0	1	0	49	102.5	188	1	1	0	0	0	1	0	140	0	1	0	0	0	0	0	0	26	0	1
169	4656	209	0	0	1	0	49	102.5	188	1	1	0	0	0	1	0	140	0	1	0	0	0	0	0	0	26	0	1
169	4656	210	0	0	1	0	49	102.5	188	1	1	0	0	0	1	0	140	0	1	0	0	0	0	0	0	26	0	1
169	4656	211	0	0	1	0	49	102.5	188	1	1	0	0	0	1	0	140	0	1	0	0	0	0	0	0	26	0	1
169	4656	212	2	0	1	0	49	102.5	188	1	1	0	0	0	1	0	140	0	1	0	0	0	0	0	0	26	0	1
169	4656	213	1	0	1	0	49	102.5	188	1	1	0	0	0	1	0	140	0	1	0	0	0	0	0	0	26	0	1
169	4656	214	1	0	1	0	49	102.5	188	1	1	0	0	0	1	0	140	0	1	0	0	0	0	0	0	26	0	1
169	4656	215	0	0	1	0	49	102.5	188	1	1	0	0	0	1	0	140	0	1	0	0	0	0	0	0	26	0	1
169	4656	216	1	0	1	0	49	102.5	188	1	1	0	0	0	1	0	140	0	1	0	0	0	0	0	0	26	0	1
169	4656	217	0	0	1	0	49	102.5	188	1	1	0	0	0	1	0	140	0	1	0	0	0	0	0	0	26	0	1
169	4656	218	0	0	1	0	49	102.5	188	1	1	0	0	0	1	0	140	0	1	0	0	0	0	0	0	26	0	1
169	4656	219	0	0	1	0	49	102.5	188	1	1	0	0	0	1	0	140	0	1	0	0	0	0	0	0	26	0	1
